# High-throughput sequencing of small RNA transcriptomes reveals critical biological features targeted by microRNAs in cell models used for squamous cell cancer research

**DOI:** 10.1186/1471-2164-14-735

**Published:** 2013-10-26

**Authors:** Patricia Severino, Liliane Santana Oliveira, Natalia Torres, Flavia Maziero Andreghetto, Maria de Fatima Guarizo Klingbeil, Raquel Moyses, Victor Wünsch-Filho, Fabio Daumas Nunes, Monica Beatriz Mathor, Alexandre Rossi Paschoal, Alan Mitchell Durham

**Affiliations:** 1 Albert Einstein Research and Education Institute, Hospital Israelita Albert Einstein, Sao Paulo, SP, Brazil; 2 Instituto de Matemática e Estatística, University of Sao Paulo, Sao Paulo, SP, Brazil; 3 Nuclear and Energy Research Institute IPEN/CNEN, Sao Paulo, SP, Brazil; 4 Division of Head and Neck Surgery, Department of Surgery, School of Medicine, University of Sao Paulo, Sao Paulo, SP, Brazil; 5 Department of Epidemiology, Faculty of Public Health, University of Sao Paulo, Sao Paulo, SP, Brazil; 6 Department of Stomatology, Faculty of Dentistry, University of Sao Paulo, Sao Paulo, SP, Brazil; 7 Federal University of Technology, Cornelio Procopio, PR, Brazil

## Abstract

**Background:**

The implication of post-transcriptional regulation by microRNAs in molecular mechanisms underlying cancer disease is well documented. However, their interference at the cellular level is not fully explored. Functional *in vitro* studies are fundamental for the comprehension of their role; nevertheless results are highly dependable on the adopted cellular model. Next generation small RNA transcriptomic sequencing data of a tumor cell line and keratinocytes derived from primary culture was generated in order to characterize the microRNA content of these systems, thus helping in their understanding. Both constitute cell models for functional studies of microRNAs in head and neck squamous cell carcinoma (HNSCC), a smoking-related cancer. Known microRNAs were quantified and analyzed in the context of gene regulation. New microRNAs were investigated using similarity and structural search, *ab initio* classification, and prediction of the location of mature microRNAs within would-be precursor sequences. Results were compared with small RNA transcriptomic sequences from HNSCC samples in order to access the applicability of these cell models for cancer phenotype comprehension and for novel molecule discovery.

**Results:**

Ten miRNAs represented over 70% of the mature molecules present in each of the cell types. The most expressed molecules were miR-21, miR-24 and miR-205, Accordingly; miR-21 and miR-205 have been previously shown to play a role in epithelial cell biology. Although miR-21 has been implicated in cancer development, and evaluated as a biomarker in HNSCC progression, no significant expression differences were seen between cell types. We demonstrate that differentially expressed mature miRNAs target cell differentiation and apoptosis related biological processes, indicating that they might represent, with acceptable accuracy, the genetic context from which they derive. Most miRNAs identified in the cancer cell line and in keratinocytes were present in tumor samples and cancer-free samples, respectively, with miR-21, miR-24 and miR-205 still among the most prevalent molecules at all instances. Thirteen miRNA-like structures, containing reads identified by the deep sequencing, were predicted from putative miRNA precursor sequences. Strong evidences suggest that one of them could be a new miRNA. This molecule was mostly expressed in the tumor cell line and HNSCC samples indicating a possible biological function in cancer.

**Conclusions:**

Critical biological features of cells must be fully understood before they can be chosen as models for functional studies. Expression levels of miRNAs relate to cell type and tissue context. This study provides insights on miRNA content of two cell models used for cancer research. Pathways commonly deregulated in HNSCC might be targeted by most expressed and also by differentially expressed miRNAs. Results indicate that the use of cell models for cancer research demands careful assessment of underlying molecular characteristics for proper data interpretation. Additionally, one new miRNA-like molecule with a potential role in cancer was identified in the cell lines and clinical samples.

## Background

MicroRNAs (miRNAs) are small RNA molecules, typically between 19 and 22 nucleotides in length, that regulate protein-coding genes through sequence-specific binding to messenger RNAs (mRNAs). These molecules were first implicated in *Caenorhabditis elegans* development in the early 90s [1], and have been associated with a variety of biological processes since then [2]. They are believed to have important roles in cancer aethiology and progression, and are currently being evaluated for cancer classification and prognosis [3].

In order to identify cell processes that are affected by miRNAs, overexpression and inhibition of miRNA genes are routinely performed for *in vitro* functional studies. Well-established cell lines are generally used for this purpose, since they are readily available from certified sources that guarantee the genetic identity. Primary cultures, on the other hand, have the advantage of not presenting genetic changes associated with the process of obtaining immortalized cell lines. It is clear, however, that experimental data will be biased by the adopted cell model and should be interpreted with caution.

Head and neck squamous cell carcinoma (HNSCC) is a smoking-related cancer for which, despite being one of the most common malignancies worldwide, reliable diagnostic and prognostic markers are not available [4]. Recent studies have addressed deregulation of microRNAs in the context of HNSCC, suggesting that these molecules could be used to improve diagnosis and the outcome of this disease [5].

Cell models are being broadly used in order to comprehend the function of specific miRNAs in this disease, but there is no current information on the miRNA content of these cells. Due to the complexity of miRNA regulatory networks, where one miRNA may target multiple genes, and where a single gene might be targeted by several miRNAs, miRNA expression levels (the miRNome) of a cell line may lead to a significant bias in functional studies results. Aiming to understand the miRNA background of cell models used for functional studies in HNSCC, we report the high-throughput sequencing analysis of the small RNA transcriptome of an oral squamous cell carcinoma cell line (SCC25) and of normal oral keratinocytes obtained from primary cultures. We believe that a deep understanding of the molecular background of cell models should greatly improve knowledge on mechanisms targeted by miRNAs and other gene regulators.

## Results

### MiRNAs expressed in the carcinoma cell line and in normal keratinocytes

We aimed to identify miRNAs that are expressed in a human carcinoma cell line and in a cell type representing its normal counterpart for miRNA functional studies in the context of HNSCC. With this purpose we chose SCC25, a tongue squamous cell carcinoma cell line, and normal oral keratinocytes derived from primary cultures. Three small RNA libraries were constructed for each cell type and sequenced on a SOLiD sequencer. Table 1 shows that, after filtering out rRNA, tRNA and other contaminants, 2.62% and 3.10% of reads accounted for miRNAs previously annotated in miRBase (release 18) for SCC25 and normal keratinocytes, respectively. When we considered only reads matching mature miRNAs, these numbers dropped to about 2% in both cases. Three hundred and thirty one mature miRNAs were identified in keratinocytes and 288 in SCC25, with 202 common miRNAs. The complete set of detected mature miRNAs for both cell types can be found in Additional file 1.

**Table 1:** Mapping of reads to genome and miRBase v18 after rRNA, tRNA, repeated DNA and adaptor sequences filtering

	**SCC25 (million)**	**%**	**Kerat (million)**	**%**
**Total of reads**	3.4	100	4.4	100
**Reads matching miRBase V18**	0.09	2.64	0.14	3.1
**Reads matching mature miRNAs**	0.07	2.00	0.9	1.94
**Reads mapped to genome and not matching v18**	0.13	3.97	0.07	4.53

**Figure 1:**
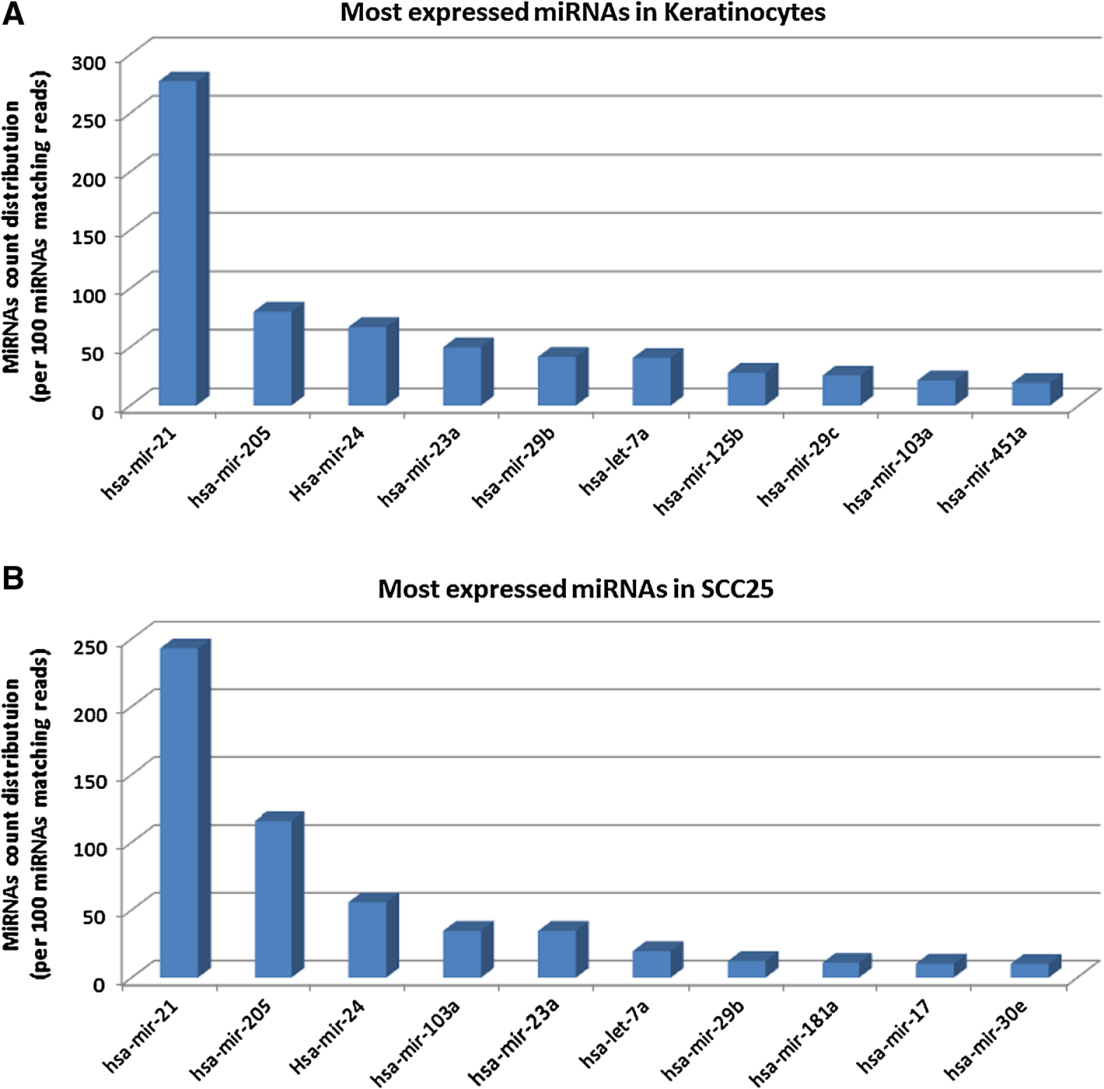
**Histogram indicating the expression levels of 10 most represented miRNAs in both datasets. (A)** 10 most expressed miRNAs in keratinocytes; **(B)** 10 most expressed miRNAs in the cell line (SCC25).

Figure 1 depicts the 10 most represented miRNAs in SCC25 and keratinocytes. These miRNAs represent 72% of reads mapping mature miRNAs in keratinocytes and 80% in SCC25. The three most expressed miRNAs were common to both datasets: miR-21, miR-205 and miR-24. Both miR-21 and miR-205 have been reported as HNSCC biomarkers [6], but here there was no differential expression between the normal cell and the cancer cell line. Noteworthy is the fact that miR-21 and miR-205 are involved in keratinocyte proliferation and migration [2,7], a common characteristic that might justify their ubiquous presence in these epithelium-derived cells. MiR-24, on the other hand, has not been implicated in HNSCC. A few reports mention a tumor suppressor function for miR-24 through the regulation of apoptosis [8]. We assessed the expression levels of miR-21 and miR-24 by real-time PCR, confirming that there was no significant difference in the expression of the two miRNAs between the two cell types (data not shown).

Together miR-21, miR-24 and miR-205 may target 66 genes (Additional file 2A). The task of identifying miRNA gene targets is an essential step for the understanding of their role in a given cellular environment. However, it is also a challenging undertaking due to the fact that miRNAs are usually imperfectly complementary to the 3′UTR region of their mRNA targets. Considering these difficulties, in order to access cellular processes targeted by the three most and commonly expressed miRNAs in the cell line and the normal keratinocyte, we selected only gene targets experimentally verified, as reported by miRecords and TarBase databases. Most of the 66 validated targets are reportedly involved in cell cycle regulation and programmed cell death (Additional file 2B). We emphasize the involvement of these miRNAs in the regulation of cyclin-dependent kinases (CDKs), key components in cell cycle regulation and traditional targets for cancer therapy development, in the regulation of SMAD and of type II receptor of TGFB (TGFBR2), components of the transforming growth factor beta signaling pathway, as well as in the regulation of NOTCH1, PI3K and PTEN. A recent review addresses the relationship between Smad/TGFBR2 and NOTCH1, PTEN and PI3K, considered relatively new players in HNSCC [9].

Only seven miRNAs were detected uniquely in keratinocytes, when a read count of at least ten reads was considered: miR-4500, miR-154, miR-337, miR-493, miR-326, miR-369, miR-381 and miR-627. Only one miRNA was uniquely expressed in SCC25 with this cut-off: miR-615. To our knowledge, no targets have been validated for these molecules up to now.

### Differential regulation of miRNAs between the cancer cell line and normal keratinocytes

**Figure 2:**
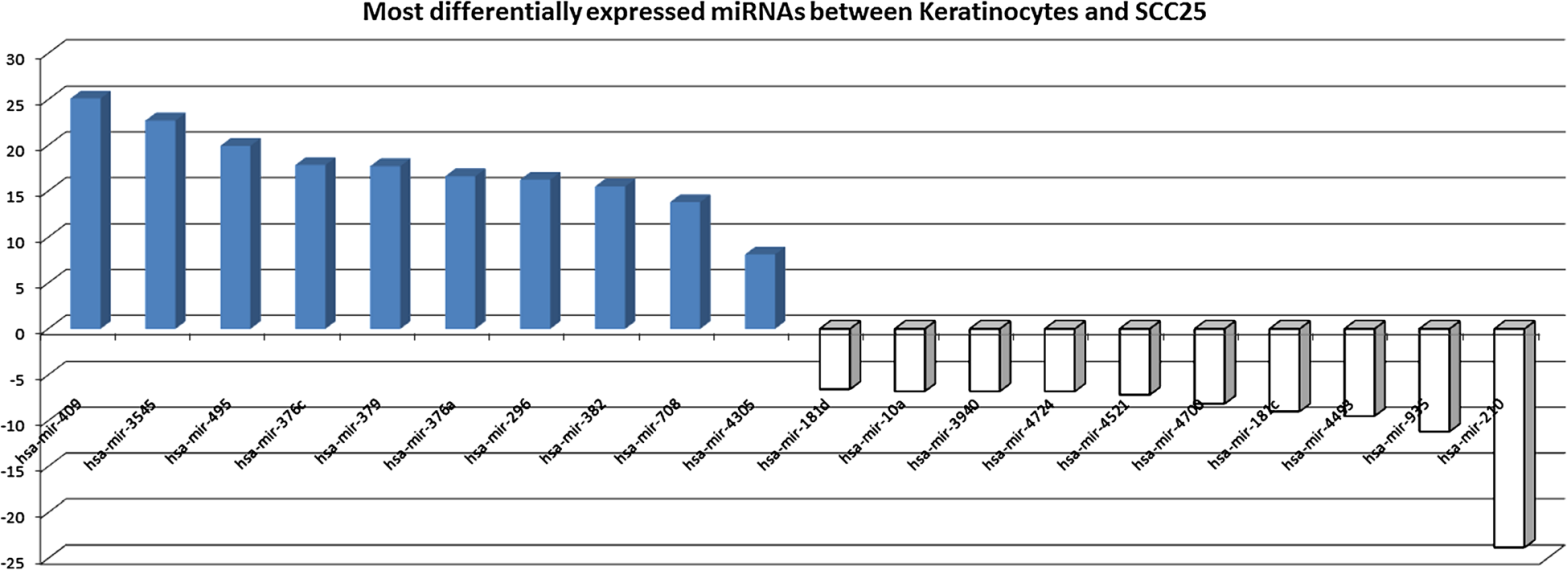
**Twenty most differentially expressed miRNAs between keratinocytes and SCC25.** The expression level of miRNAs mostly expressed in keratinocytes is represented in blue and in white is the expression of those more expressed in the cell line.

The relative abundance of miRNAs was then compared between the carcinoma cell line and normal keratinocytes. Among the 202 miRNAs expressed in both cell types, 65 miRNAs were overexpressed in keratinocytes and 42 in SCC25, when a 2-fold change in expression was considered. This result is in agreement with previous findings reporting overall lower levels of miRNAs in cancer as compared to normal tissues. Twenty miRNAs presenting higher fold-changes are depicted in Figure 2, where blue bars correspond to miRNAs mostly expressed in keratinocytes and white bars correspond to miRNAs mostly expressed in the cell line. Additional file 3 summarizes differences in gene expression for 107 miRNAs expressed with a 2-fold change between keratinocytes and the cell line.

From the 107 miRNAs, we found experimentally validated targets for only 6 molecules – miR-1, miR-125b and miR-133a up-regulated in keratinocytes and miR-196a, miR-511 and miR-7 up-regulated in the cell line. Among these, we chose to confirm by real time PCR the deregulation of the miRNAs presenting the lowest level of deregulation – miR-1 and miR-133a, up-regulated ~ 2-fold in keratinocytes, and of miR-196a, up-regulated ~ 2 fold in the cell line. Results between real time PCR and sequencing were consistent (data not shown). These 6 miRNAs may target 315 gene targets (Additional file 4A). Programmed cell death-related genes were the most represented among targeted genes, mostly grouped under the GO term Regulation of Apoptosis (GO:0042981, FDR corrected p value < 0.05) (Additional file 4B). This result indicates differences in apoptosis regulation between the cancer cell line and the normal counterpart. Illustrating the importance of this scenario for HNSCC, p53, a key tumor suppressor frequently mutated in HNSCC [10] is a validated target of miR-125b [11], and this miRNA has also been recently implicated in the carcinogenesis of squamous cell carcinomas [12].

Cell differentiation-related genes, grouped under GO term Positive Regulation of Cell Differentiation (GO:0045598, FDR corrected p value < 0.05), were also enriched in the dataset (Additional file 4B), an expected result considering intrinsic differences between a cell line and a keratinocyte derived from primary cultures, the later retaining its full capacity for cell differentiation.

Top over-expressed miRNAs in keratinocytes included miR-409, reportedly a tumor suppressor in gastric cancer [13], miR-3545, a recently described miRNA corresponding to the antisense strand of miR-203, which is a miRNA preferentially expressed in the skin, and which has been described as a tumor suppressor molecule silenced in different malignancies [14,15], and miR-376c, shown to induce apoptosis and described as down regulated in HNSCC [16].

MiR-495 and miR-379 have not been previously associated with HNSCC biology or epithelial differentiation, but miR-495 has been reported to be a tumor suppressor [17] and miR-379 has been shown to be down-regulated in HPV positive HNSCC samples [18].

Among top over-expressed miRNAs in SCC25, miR-210 and miR-181c have been previously associated with HNSCC. MiR-210 has been described as a marker for tumor hypoxia and a prognostic factor in HNSCC [19]. MiR-181c, reported as up-regulated in tongue squamous cell carcinoma (TSCC), was the second most up-regulated miRNA in SCC25 [20].

Taken together these results highlight important differences between a cancer cell line derived from the oral cavity, and its normal counterpart, a keratinocyte derived from a primary culture. These differences are consistent with the genetic background they represent and should be taken into consideration when cell models are chosen.

### mRNA expression patterns in the cell line and keratinocytes

DNA microarrays were used in order to evaluate gene expression differences between keratinocytes and the cancer cell line that might support processes targeted by the differential miRNA expression. Even though miRNAs are mostly posttranscriptional regulators and their effect on mRNA abundance would be small in our cell models, we aimed to access differentially regulated biological processes and, when possible, associate these differences with miRNA regulation potential. A total of 978 genes were over-expressed in SCC25 when a 2-fold difference in gene expression was considered, while 523 were down-regulated (FDR adjusted p value of 0.05) (Additional file 5).

Gene Ontology term enrichment analysis of the differentially expressed genes dataset identified processes associated with cell cycle regulation (cell cycle progression, chromosome segregation and DNA replication) enriched when genes up-regulated in SCC25 were considered (Additional file 6A). Genes up-regulated in keratinocytes were mostly involved with epithelium development (Additional file 6B). These results are mostly in agreement with expected differences between the cell types, since up-regulation of proliferation-related processes should be a characteristic of a cancer cell line and epithelium development of a normal keratinocyte.

We then looked for gene targets for the 7 miRNAs that possess experimentally validated targets addressed in the previous section – miR-1, miR-133a and miR-125b up-regulated in keratinocytes and miR-196a, miR-511 and miR-7 up-regulated in the cell line – in the dataset of deregulated mRNAs. A total of 21 targets were found among deregulated genes (Table 2). However, only eight of these targets presented expression levels inversely correlated with the related miRNA, an indication of regulation. Although the correlation of expression levels does not confirm the regulation of targets by a miRNA, nor does the lack of gene expression differences indicate that the targets are not being regulated - since regulation might not be at the gene expression level and we did not check protein levels - we chose to present only targets previously shown to be regulated at the gene expression level.

**Table 2:** Deregulated miRNA targets in dataset comparing mRNA expression between the cell line and keratinocytes

**Gene target**	**Up-regulated in cell line**	**Up-regulated in Keratinocyte**	**Related miRNA**
**ADAR**	**3.6**		miR-1
**ANKRD29**		4.1	miR-1
**ANP32B**	**3.4**		miR-1
**ATP6V1B2**		2	miR-1
**CDCP1**		2.3	miR-1
**EPB41L4B**		4.3	miR-1
**FSTL1**		4.7	miR-1
**KCNQ1**	**8**		miR-1
**POLA2**	**2.9**		miR-1
**PREX1**	**12**		miR-1
**SSNA1**	**2.6**		miR-1
**UST**	**4.6**		miR-1
**CDK9**	**2.3**		miR-1
**B3GALT4**	2.7		miR-125
**BMF**	31		miR-125
**CDKN2A**		**21**	miR-125
**IGFBP3**	700		miR-125
**RHOA**		2.3	miR-133a
**SET**	2.5		miR-196a
**SPRR2C**		**71**	miR-196a
**SNCA**		**5**	miR-7

The table reports 21 validated gene targets presenting deregulation between SCC25 and keratinocytes. Fold-change in bold-type indicates that the fold-change is in agreement with the expression level of the regulator miRNA in the cell.

### Identification of miRNA-like molecules

**Figure 3:**
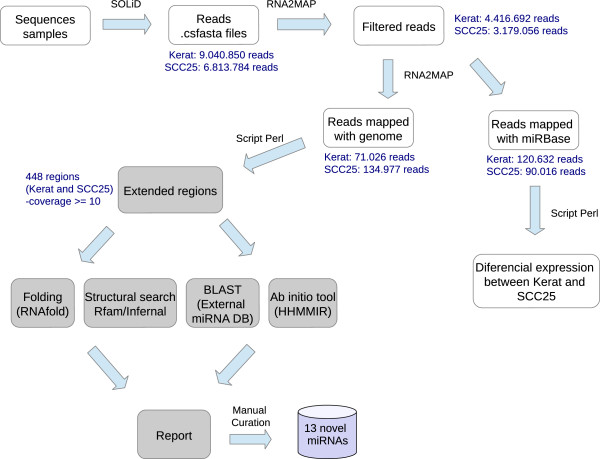
**Workflow for the analysis of small RNA transcriptome focusing on miRNA identification and discovery.** Each library was converted to FASTA format, subjected to RNA2MAP tool and reads that did not match the human genome were discarded. After the filtering protocol, that matched miRBase were submitted to differential expression analysis and those that did not match were mapped to the genome. At each genomic locus the read was extended by100 nt up and downstream. Resulting sequences were subjected to the following pipeline: secondary structure prediction using RNAfold and Infernal against RFAM, blast against a local non-coding RNA database and, ab intio characterization using HHMMiR and RNAFold (HHMMiR in fact is performed using the data produced by RNAfold).

For the identification of novel miRNA-like molecules, all reads shorter that 35 nt, having more than 10 copies in our libraries and no match to known miRNAs were tested. Reads were mapped to the genome and, at each genomic locus of a read to be tested, one longer sequence covering the read was extracted for secondary structure analysis. This sequence extended 100 nt upstream and 100 nt downstream from the read and this length was established following the average length of known human precursor miRNAs (Additional file 7). A set of 448 sequences obtained this way was submitted to a four-step annotation procedure: (i) sequence similarity search with the BLAST program against a locally curated ncRNA database; (ii) structural search against all RFAM families using INFERNAL, (iii) *ab initio* characterization using RNAfold and HHMMiR; (iv) manual curation of the results (Figure 3). Candidates that mapped to known coding regions were discarded. Of the 448 sequences, 13 presented some evidence in at least one of the procedures. The results are shown in Table 3.

**Table 3:** Putative miRNAs identified using similarity search, structural search and ab initio prediction

		**Cell line count(normalized)**		**Blast search against miRNAs in local DB**^**1**^	**HHMMR**		**RNAfold**	**Rfam/INFERNAL**^**2**^						
**Candidate number**	**Genomic location**	**Kerat**	**SCC5**		**score**	**Original read overlaps Helix?**	**Keal/mol**	**Family**	**ID**	**Strand**	**Bit score**	**Start**	**End**	**Original reads overlaps Helix?**
1	Intergenic	3.9	9.6	-	0.67	No	-72,10	-	-	-	-	-	-	-
2	Intergenic	46	82.5	-	0.69	No	-64,49	-	-	-	-	-	-	-
3	Intron	4.4	-		0.69	Partially	-51,30	-	-	-	-	-	-	-
4	Intergenic	3.9	51.6	-	0.66	Partially	-44,30	**MIR245**	**RF00816**	**+**	**29.76**	**97**	**182**	**Total**
5	Intron	-	8	-	0.7	No	53,52	-	-	-	-	-	-	-
6	Intergenic	-	4.8	-	0.66	No	-48,90	-	-	-	-	-	-	-
7	Intergenic	-	78.9	**Original DB**: smiRNAdb; **Type**: unclassified RNA **QueryCov**: 13%; **SbjCov**: 97% **Id**: 100%	0.66	No	-57,34	-	-	-	-	-	-	-
8	Intron	-	5.6	**Origianl DB**: smiRNAdb; **Type**: miRNA; **QueryCov**: 11%; **SbjCov**: 96% **Id**: 100%	-	-	57,10	-	-	-	-	-	-	-
9	Intergenic	-	11.2	**Original DB**: smiRNAdb; **Type**: unclassified RNA; **QueryCov**: 13%; **SubjectCov**: 94%; **Id**: 100%;	0.67	Total	-35,35	**mir-450**	**RF00708**	**+**	**30.4**	**32**	**112**	**Parcial**
								**mir-245**	**RF00816**	**+**	**29.76**	**35**	**120**	**Parcial**
10	Intergenic	-	4.8	-	-	-	-122,82	**mir-689**	**RF00871**	**+**	**66.15**	**17**	**91**	**No**
11	Intron	-	4.8	-	0.66	Total	-83,69	-	-	-	-	-	-	-
12	Intron	-	4.0	**Original DB**: smiRNAdb; **Type**: unclassified RNA; **QueryCov**: 12%; **SubjectCov**: 100%: **Id**: 100%	0.68	Total	-50,30	-	-	-	-	-	-	-
13	Intron	-	15.2	-	0.67	No	-94,14	-	-	-	-	-	-	-

Notation: Original DB: Database from which sequence was downloaded into local database: Type: RNA type (misRNA if not specified); Query Cov: percentage of database sequence covered in the alignment; Subject Cov: percentage of candidate covered by the alignment; Id: alignment identity; 1: local miRNA database with sequences from databases designated by the Non-coding RNA Databases Resource (NRDR).

Four candidates presented good BLAST alignments with sequences in ncRNA databases (Cand7, Cand8, Cand9 and Cand12) and, of these one aligned with a ncRNA classified as miRNA (Cand8). Three candidates (Cand4, Cand9 and Cand10) presented good structural alignments to RFAM families, but of these only two alignments included the corresponding original reads (candidates Cand4 and Cand9) and only two candidates aligned to RFAM families that included mammalian sequences (Cand9 and Cand10). All candidates but for Cand8 and Cand10 were predicted by HHMMiR as including miRNAs, however only candidates Cand3, Cand4, Cand9, Cand11 and Cand12 had the original reads included in predicted stems (Additional file 8).

Five of the thirteen candidates showed evidence of the original read mapping (totally or partially) in a predicted stem in the precursor sequence considered (Additional file 8). These full sequences (reads plus extensions constituting putative precursors) were analyzed with the computational tool MatureBayes for the identification of possible mature sequences. As depicted in the Additional file 9, the algorithm did not find a potential functional part in the proposed precursor sequences (putative mature miRNAs) that contained the original read. This analysis underlines difficulties in novel miRNA discovery, showing that different approaches are needed for complementarity.

### Comparison of results in cells with clinical samples

**Figure 4:**
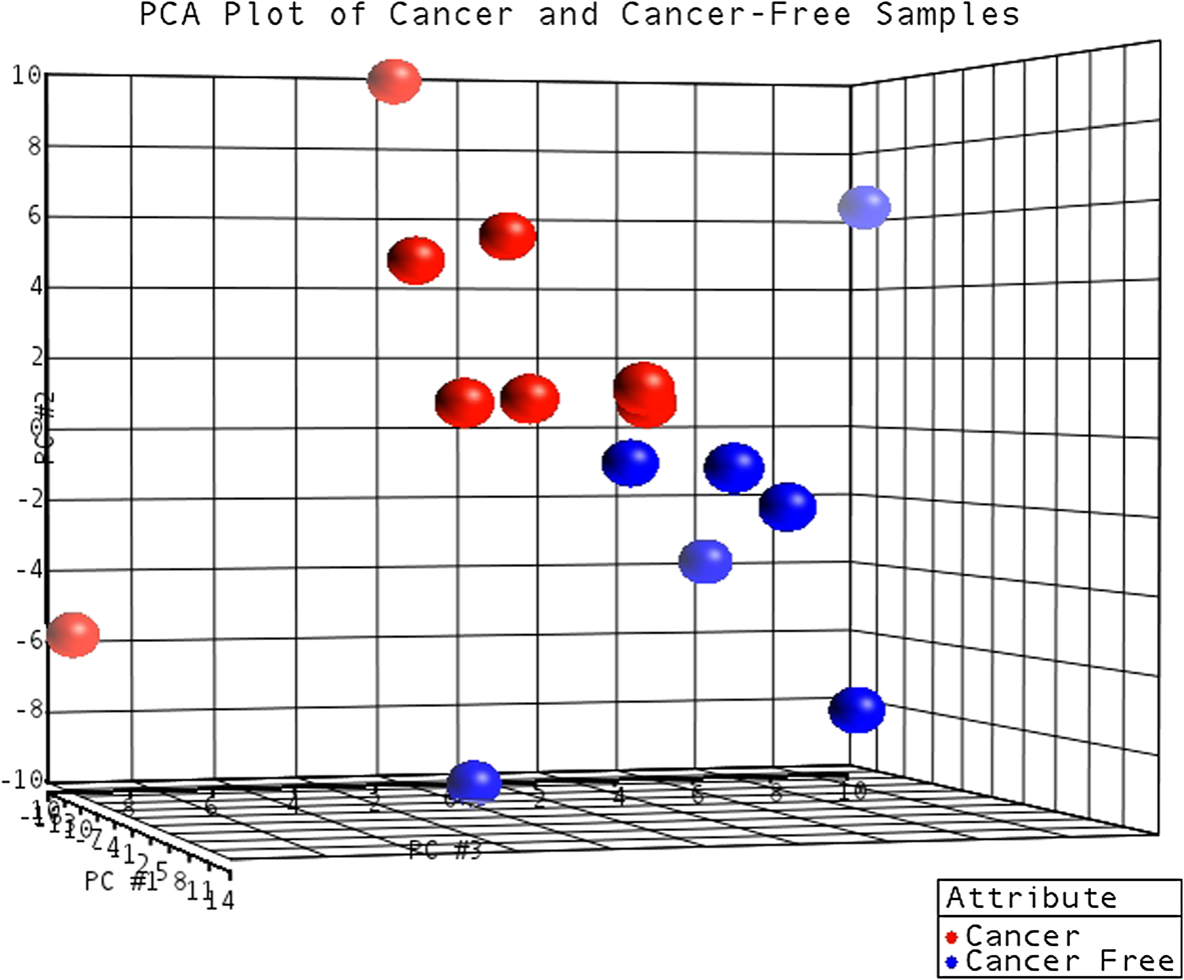
**Unsupervised classification by principal components analysis of cancer and cancer-free samples.** Principal components analysis (PCA) was used to classify 15 samples (8 cancer samples and 7 cancer-free samples) based on the expression profile of 193 miRNAs expressed in all samples. Sample 306 M was not included in this analysis due to its high heterogeneity. The PCA plot depicts 70% of variability in the dataset.

In order to compare miRNAs expressed in cell cultures with clinical samples, small RNA libraries from eight HNSCC patients were constructed and sequenced, following the same procedures described for the cells (results in Additional file 10). In each case we sequenced a tumor sample and surgical margins, the later representing cancer-free tissue. A PCA plot illustrates global characteristics of these samples: despite heterogeneity within the two groups, the expression level of shared molecules is able to differentiate cancer from cancer-free samples (Figure 4). Table 4 shows that similarities in miRNA population, when cancer samples were compared to their cell model counterpart, varied from 46% to about 70%. Since these percentages did not correlate with the sequencing coverage obtained in this study, we concluded that sequencing depth for each clinical sample was adequate.

**Table 4:** Common miRNAs identified in cells and clinical samples.

**Sample**	**Total of miRNAs**	**Common miRNAs**	**% of common miRNAs with related cell type**
**196 M**	313	219	69.97
**196 T**	279	178	63.80
**240 M**	321	223	69.47
**240 T**	298	184	61.74
**277 M**	558	294	52.69
**277 T**	335	187	55.82
**296 M**	642	309	48.13
**296 T**	393	212	53.94
**306 M**	589	286	48.56
**306 T**	641	269	41.97
**321 M**	495	273	55.15
**321 T**	515	243	47.18
**333 M**	350	240	68.57
**333 T**	503	246	48.91
**349 M**	585	292	49.91
**349 T**	480	221	46.04

Tumor samples were compared to the cancer cell line and tumor-free samples with normal keratinocytes.

**Figure 5:**
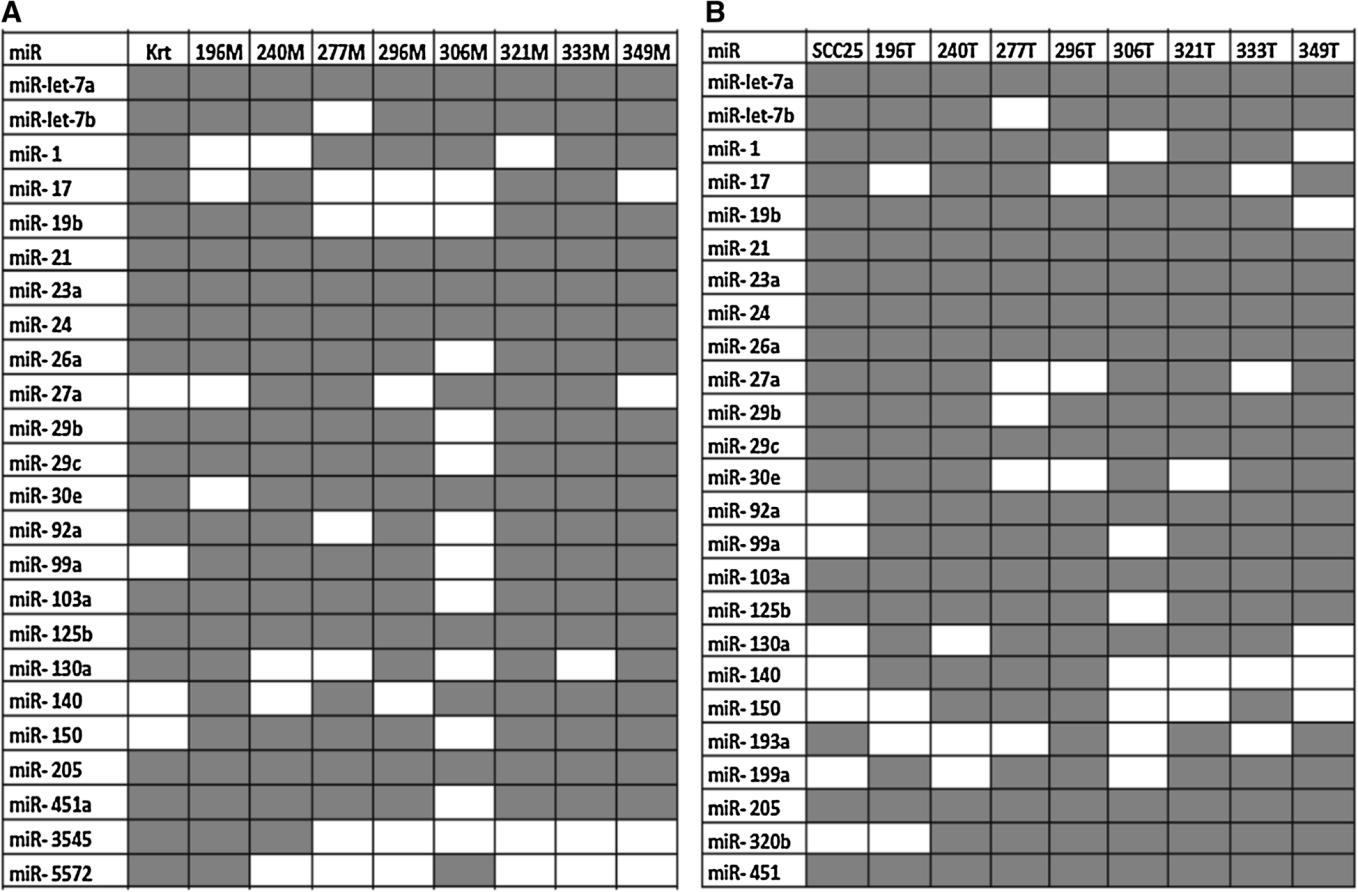
**Most expressed miRNAs in keratinocytes, cell line and in clinical samples. A**: Most expressed miRNAs in keratinocytes (Krt) and in tumor-free samples; **B**: Most expressed miRNAs in the cancer cell line (SCC25) and in tumor samples. MiRNAs are reported in alphabetical and numerical order. The presence of a given miRNA in the dataset is indicated by gray color.

Despite differences in absolute quantities, miRNAs highlighted as most expressed in cells are frequently present among the most expressed miRNAs in clinical samples (Figure 5). Noteworthy is again the widespread expression of miR-21, miR-24 and miR-205, already addressed in cells. This result corroborates the assumption that these abundant molecules are involved in processes common to epithelial cells and/or maintenance, and do not seem to be affected by the cancer phenotype.

On the other hand, the scenario describing the differential expression between cancer samples vs cancer-free tissue is more complex and not directly comparable with results obtained for cell lines. This is expected due to the complexity of the addressed tumor type. For instance, miR-409, presenting the highest fold-change between the keratinocytes and the cancer cell line was over-expressed in cancer-free tissue in only four patients, while miR-210, highly over-expressed in the cell line when compared to keratinocytes, was not differentially expressed in our clinical samples. In Additional file 11 we show the comparisons between common miRNAs in cancer and cancer-free tissue and their cell counterpart.

Finally, we considered the expression levels in the genomic region containing the miRNA candidate proposed in this study. Table 5 depicts the expression of the two reads that constitute the candidate in each clinical sample. Samples 196, 306, 312, and 333 showed similar levels of expression found in cells. Results suggest that the molecule might be functional and have a role in HNSCC.

**Table 5:** Expression of Cand4 and Cand9 (reads from predicted miRNA) in clinical samples

**Sample 196**	**Read count (Raw)**		**Normalized read count**	
	**196 M**	**196 T**	**196 M**	**196 T**
**Cand99**	159	129	41.19	63.66
**Cand209**	4	1	1.04	1.60
**Sample 240**	**Read Count (Raw)**		**Normalized Read Count**	
	**240 M**	**240 T**	**240 M**	**240 T**
**Cand99**	1076	26	278.77	10.41
**Cand209**	40	-	10.36	-
**Sample 277**	**Read Count (Raw)**		**Normalized Read Count**	
	**277 M**	**277 T**	**277 M**	**277 T**
**Cand99**	391	16	101.30	6.41
**Cand209**	8	15	2.07	6.01
**Sample 296**	**Read Count (Raw)**		**Normalized Read Count**	
	**296 M**	**296 T**	**296 M**	**296 T**
**Cand99**	1566	335	405.72	134.13
**Cand209**	9	10	2.33	4.00
**Sample 306**	**Read Count (Raw)**		**Normalized Read Count**	
	**306 M**	**306 T**	**306 M**	**306 T**
**Cand99**	222	166	57.52	66.46
**Cand209**	2	3	0.52	1.20
**Sample 321**	**Read Count (Raw)**		**Normalized Read Count**	
	**321 M**	**321 T**	**321 M**	**321 T**
**Cand99**	86	170	22.28	68.06
**Cand209**	-	1	-	0.40
**Sample 333**	**Read Count (Raw)**		**Normalized Read Count**	
	**333 M**	**333 T**	**333 M**	**333 T**
**Cand99**	164	985	42.49	394.38
**Cand209**	-	3	-	1.20
**Sample 349**	**Read Count (Raw)**		**Normalized Read Count**	
	**349 M**	**349 T**	**349 M**	**349 T**
**Cand99**	177	50	45.86	20.02
**Cand209**	-	1	-	0.40

Samples 196, 306, 321 and 333 presented expression levels similar to findings in cells.

## Discussion

Complex regulatory networks connect genes within cellular processes. MiRNAs have been recently added to this scenario, constituting an additional layer in gene regulation. The involvement of miRNAs in fundamental biological processes such as cell differentiation and programmed cell death, as well as their implication in innumerous human diseases, are currently known. The comprehension of their roles at a systems-level, however, is far from complete.

Once a miRNA is identified as a player in a pathological condition, functional studies using cell models are commonly used to address its involvement in gene regulation and consequent phenotypes. Due to the complex interactions between miRNAs and gene targets, the knowledge regarding the expression of miRNAs in available cell models should be carefully considered before tackling miRNA functional assays. Additionally, the response of cell lines to different stimuli varies due to, among other issues, their particular genetic background. For squamous cell carcinoma cell lines, distinct patterns of proliferation and survival upon treatment with drugs or environmental stress have been reported in the literature [21-23]. Regulation of miRNAs certainly impacts these conclusions, even though it was not addressed in the above-mentioned studies.

In this study we addressed the miRNA constitution of a cancer cell line and normal oral keratinocytes, both used as models for head and neck squamous cell carcinoma and its cancer-free counterpart, respectively. Our results comparing the expression of miRNAs expressed in both cell types corroborate literature findings. For instance, the expression of miR-205 was comparable in both cell types, in agreement with its description as a marker of squamous epithelia [6], and higher expression levels of miR-125b in keratinocytes corroborates recently published data implicating the loss of miR-125 and HNSCC carcinogenesis [12]. On the other hand, unexpected results such as the finding of miR-21 equally expressed between the cell models, a miRNA commonly deregulated in cancer [24], was confirmed by differences in the expression level of this miRNA found between our clinical samples.

Through the relationship between miRNA expression levels and the targets they could be regulating we show that important cancer hallmarks i.e. cell death and cell proliferation regulation, could be intensely affected by the expression of these molecules. On the other hand, we also demonstrate that the expression of certain miRNAs, and, consequently, processes they target, might be independent of the cancer phenotype. For this result we addressed miRNAs that did not show differences in expression between the cell types and their experimentally validated gene targets reported in the literature. As expected, expression levels of miRNAs in clinical samples varied and did not always correlate with findings in the cell models.

**Figure 6:**
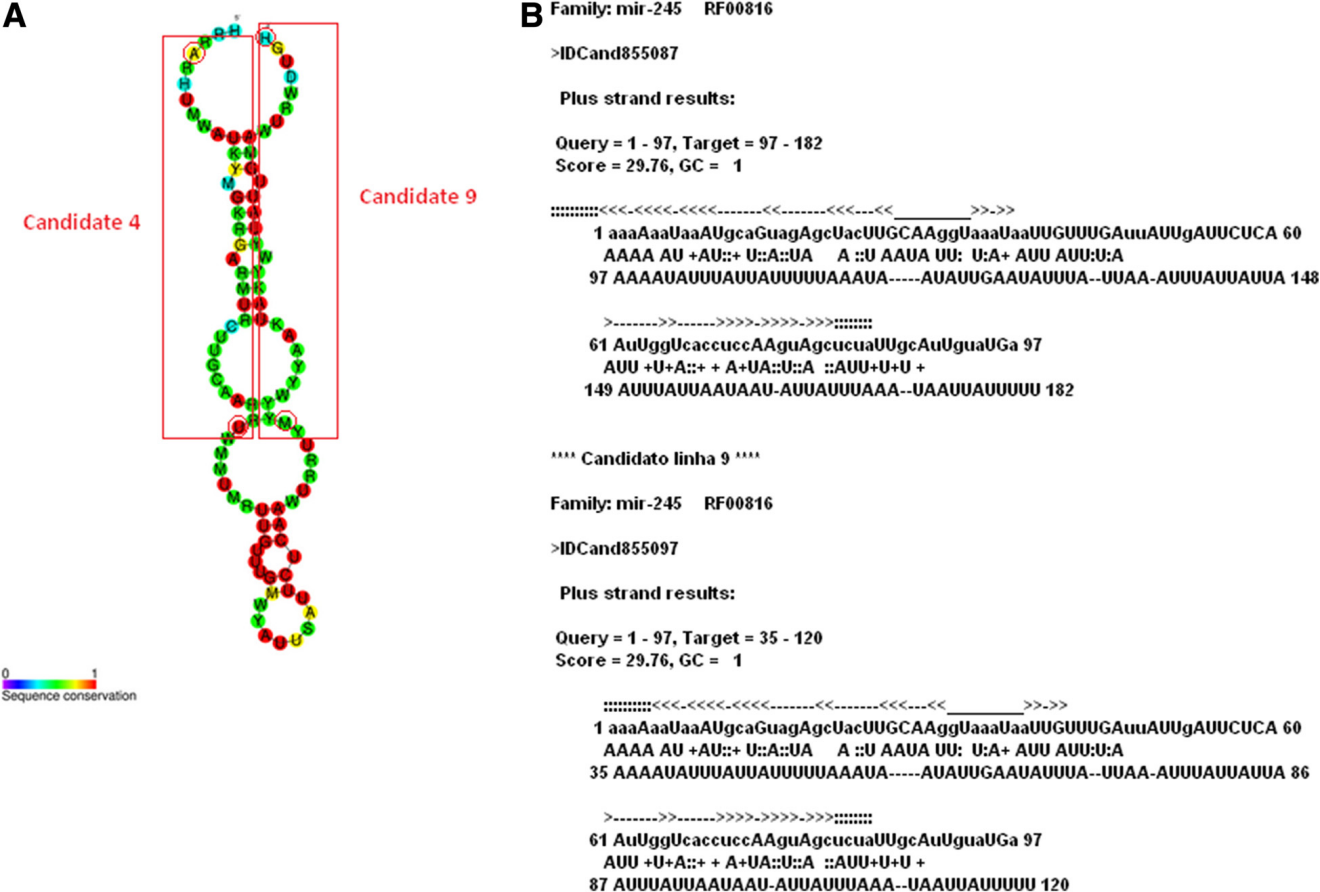
**Structural alignment of candidates Cand4 and Cand9 against RFAM family RF00816. A**: Secondary structure of family RF00816 and regions corresponding to the original reads of candidates Cand4 and Cand9; **B**: INFERNAL alignment of candidates Cand4 and Cand9 against family RF00816 - the original reads correspond to positions 100–130 of each candidate.

We have also performed an *in silico* analysis to search for new miRNA candidates. It is currently known that high throughput sequencing technologies allow for the discovery of novel molecules, due to their inherent sensibility and accuracy. The analysis used structural alignment against known miRNA families, *ab initio* prediction using HHMMiR and RNAfold, and similarity search against a curated miRNA local database. Since we were sequencing small RNAs, we postulated that any miRNA sequenced in the process was a mature miRNA, and therefore had to be part of a stem in the precursor miRNA secondary structure. Of the 13 candidates with any miRNA evidence, in only five there was any evidence that the original read mapped totally or partially in a predicted stem (Cand03, Cand04, Cand09, Cand11, Cand12), these will be discussed in detail. Cand3 had only a HHMMiR prediction, which partially included the original sequence in a stem, and was expressed only in keratinocytes. Cand11 presented a good HHMMiR prediction, and was expressed only in SCC25 cells. Cand12, had evidence coming from a HHMMIR positive prediction and an alignment against an uncharacterized small ncRNA in smiRNAdb. Cand4 had a positive HHMMiR prediction and an alignment to a known miRNA family (RF00816). Cand9 also mapped to the same RFAM family (RF00816), with the original read located in a predicted stem. In fact, the genomic mapping of the candidates showed that they Cand4 and Cand9 correspond to adjacent genomic locations (Figure 6). The original reads of each candidate covered both sides of the same predicted stem in the miRNA family. Additionally both candidates were more expressed in SCC25 cells when compared to keratinocytes (51.6 vs 3.9 for Cand4, 11.2 vs. 0 for Cand9). A close investigation of the RF00816 family in RFAM shows miRNAs have been characterized for 14 different species (6 species of nematodes and 8 species or arthropods). Finally Cand9 had a hit against an undefined small ncRNA deposited in the smiRNAdb database. Cand9 also had a structural alignment to the RFAM family RF00708, but this alignment included only 12 bases of the original read, and in spite of the fact that the family has been characterized in human, the alignment did not cover the whole consensus sequence.

All candidates were also submitted to the software MatureBayes, which identifies mature sequences within putative precursors, but in none of the candidates the original read was considered as a mature miRNA. However, in this software, the definition of the precursor sequence could significantly impact the results. Our putative precursor sequences were based on the average length of precursors in humans. The extension of 100nt on both sides of the read derives from the fact that we did not have enough information to the position the read within its precursor. This may have affected the quality of the software’s predictions.

This conjunction of evidences point to the genomic region comprising candidates Cand4 and Cand9 as a strong candidate for a new miRNA gene. Other two candidates, Cand11 and Cand12, also presented consistent evidence, but based only on HMMIR, which is heavily dependent on the folding provided by the RNAfold algorithm, and suffers from the same dependence of the putative precursor sequence as MatureBayes.

The genomic region comprising the strongest candidate for a new miRNA was evaluated in clinical samples. Expression levels of the sequences that originated Cand4 and Cand9 were up-regulated in several tumor samples, when compared to cancer-free surgical margins, suggesting that this molecule might, indeed, have a role in HNSCC. Further experiments are necessary in order to confirm if the molecule is truly a miRNA.

## Conclusions

MiRNA content of two cell models used for HNSCC cancer research was characterized by deep sequencing. Several miRNAs were equally expressed between a cancer cell line and keratinocytes, suggesting that the regulation of processes targeted by these molecules may be independent from the cancer phenotype. On the other hand, we provide evidences that pathways commonly deregulated in HNSCC, such as apoptosis and cell differentiation, may be targeted by miRNAs differentially expressed between cell types. Our results also imply that the use of cell models for microRNA functional studies in cancer research demands careful assessment of underlying molecular characteristics for proper data interpretation. The characterization of putative novel microRNA molecules carried out here revealed one strong new miRNA gene to be experimentally validated. This candidate was mostly expressed in the cancer cell line and its expression was validated in clinical samples, indicating a possible role in cancer.

## Methods

### Cell culture

We used the HNSCC cell line SCC25, derived from a SCC of the tongue for small RNA transcriptome sequencing. It was obtained from the American Type Culture Collection (ATCC catalog number CRL-1628). For the purpose of this study, the cell line was grown in a Dulbecco’s Modified Eagle’s medium/Nutrient Mixture F-12 Ham (DMEM/F12) supplemented with 10% fetal bovine serum in a humidified atmosphere of 5% CO^2^ and 95% air at 37°C.

Human oral epithelial tissue was obtained from healthy volunteers undergoing dental surgeries, following previously published procedures [6], after approval by the Research Ethics Committee of IPEN under License Number 087/CEP-IPEN/SP and with informed consent signature. Keratinocytes used for small RNA transcriptome sequencing and gain-of-function experiments were grown on a fibroblast feeder-layer in a Dulbecco’s Modified Eagle Medium (DMEM; Gibco, New York, NY, USA) F-12 Nutrient Mixture (HAM, Gibco, New York, NY, USA) (2:1), with 10% Bovine Serum Product Fetal Clone III (Hyclone, Logan, Utah, USA), penicillin (100 U/ml), streptomycin (100 lg/ml), gentamicin (50 lg/ml), and amphotericin B (2.5 lg/ml) glutamine (4 mM), adenine (0.18 mM) (Sigma–Aldrich, St. Louis, MO, USA), insulin (5 lg/ml) (Sigma–Aldrich, St Louis, MO, USA), hydrocortisone (0.4 lg/ml) (Sigma–Aldrich, St Louis, MO, USA), cholera toxin (0.1 nM) (Sigma–Aldrich, St Louis, MO, USA), triiodotyronine (20 pM) (Sigma–Aldrich, St Louis, MO, USA) and epidermal growth factor (10 ng/ml) (R&D Systems, Minneapolis, MN, USA).

### Clinical samples

Eight patients with oral squamous cell carcinoma (tongue and floor of the mouth) were selected for this study. The clinical and pathological profile of patients is shown in Table 6. Tumor and corresponding cancer free surgical margins containing the corresponding epithelium were collected from patients submitted to surgical resection of primary tumor at Hospital das Clinicas, Hospital Heliopolis and Arnaldo Vieira de Carvalho Cancer Institute, in Sao Paulo, Brazil. All patients provided written informed consent, and the research protocol was approved by review boards of all institutions involved and by the National Committee of Ethics in Research (CONEP 1763/05). Samples were snap-frozen in liquid nitrogen immediately after surgery. Analysis of hematoxylin and eosin-stained sections confirmed that >75% tumor cells in all HNSCC samples and that surgical margins were tumor-free.

**Table 6:** Clinical data of patients in this study

**Patient**	**Tumor site**	**Gender**	**Age (yr)**
196	**OC-T**	**Male**	**49**
240	**OC-FOM**	**Male**	**75**
277	**OC-T**	**Male**	**66**
296	**OC-FOM**	**Male**	**53**
306	**OC-FOM**	**Female**	**82**
321	**OC-FOM**	**Male**	**69**
333	**OC-T**	**Male**	**52**
349	**OC-FOM**	**Male**	**59**

OC-T: Oral Cavity – Tongue, OC-FOM: Oral Cavity - Floor of the Mouth.

### Small RNA library construction and sequencing

Total RNA was obtained from confluent cell cultures or clinical samples using the mirVana Isolation Kit (Ambion Inc., USA). The concentration and quality were determined using a Nanovue spectrophotometer (GE Healthcare). Library construction followed, strictly, the SOLiD Total RNA-Seq Kit for Small RNA Libraries protocols (Ambion Inc., USA). Three libraries were constructed for each cell type. Eight hundred ng of total RNA were used as a template to obtain the small RNA library and we used the SOLiD RNA Barcoding System (Ambion Inc., USA) for library multiplexing. The SOLiD3 sequencing system (Life Technologies, CA, USA) was used to generate reads that were 35 bp long. Default parameters were used at all instances during sequencing.

### Sequencing data analysis

Sequence analysis was performed using the Small RNA Analysis Tool (RNA2MAP) [25] using the following parameters: three color-space mismatches within the 'seed sequence’ (first 18 bases of the reads), and six color-space mismatches on the following positions of the 35 bp reads. All sequences that matched tRNA, rRNA, DNA repeats and adaptor molecules were filtered out and then the remaining reads were matched against miRNA precursor sequences in miRBase release 18. Then, using a Perl script we selected only reads containing mature sequences.

The RNA2MAP mapping tool generated two types of alignment: reads uniquely mapped to miRNAs and reads generating multiple hits. In order to select for molecules most likely to represent a mature miRNA we restricted multiple alignments to 5 hits and such hits should be within variations of a single miRNA family. For instance, reads mapping identically to hsa-mir-103a-1 and hsa-mir-103a-2 counted as "hsa-mir-103a". Reads generating multiple hits that did not conform to these parameters were discarded.

Following the identification of miRNAs present in each sample, we also used Perl scripts to compare normal keratinocytes and SCC25, and cancer and cancer-free patients.

To compare the expression data levels, the expression of each mature miRNA was normalized using the highest expression value in the dataset [26] and miRNAs presenting a difference in expression level of at least 2-fold were considered differentially expressed between datasets.

For data visualization of clinical samples, expression patterns of miRNA detected in every sample were clustered by Principal Components Analysis (PCA) using Partek Genomics Suite (v6.6).

The miRNA targets were searched using Ingenuity Pathway Analysis ( http://www.ingenuity.com), through the integration of Tarbase and miRecords databases. For pathway mapping and Gene Ontology term enrichment analysis we used DAVID Bioinformatics Resources ( http://david.abcc.ncifcrf.gov).

### Relative quantitation of gene expression by real-time PCR

For miRNA expression analysis, the cDNA was synthesized from 100 ng of total RNA using sequence-specific stem-loop primers for hsa-miRNA-1, hsa-miRNA-7, hsa-miRNA-21, hsa-miRNA-24, hsa-miRNA-133, hsa-miRNA-196 and for the endogenous control RNU48 (TaqMan miRNA RT kit, Life Technologies, Carlsbad, CA, USA). The relative quantitation of miRNA was carried out using the Taqman Universal PCR Master Mix (Life Technologies, Carlsbad, CA, USA), according to the manufacturer’s instructions. Data was normalized to the expression of RNU48 and analyzed using the delta delta Ct method.

### Microarray analysis

Microarray analysis was performed using Agilent Whole Human Genome Microarray 4 × 44 K arrays and labeled using the One Color Quick Amp Labeling Kit (Agilent Technologies). A total of four samples were analyzed: two biological replicates of SCC25 and two biological replicates of normal keratinocytes. Hybridization and washing followed protocols described by the manufacturer (Agilent Technologies). The one-color arrays were scanned by GenePix 4000B Scanner (Axon), and analyzed using the Agilent Feature extraction software (version 9.5). The quality control of the microarrays was assessed using the standard Agilent controls to verify that the arrays met the expected criteria. The gProcessedSignal from each array was loaded into Partek Genomics Suite (v6.6) and normalized between arrays using quantile normalization. For subsequent statistical analysis we used the ANOVA implementation of Partek. Differences between cell lines and between clinical samples are presented in fold-changes. The raw data can be assessed at Gene Expression Omnibus under the accession number GSE41436.

### Discovery of novel miRNA

All reads that did not match miRBase v18 were subjected to a bioinformatics pipeline represented in Figure 3. Reads were initially clustered based on their genomic positions and extended by 100 nucleotides upstream and downstream from their genomic coordinates. The size of the extension was based on an analysis of the sizes of known precursor human miRNAs deposited in miRBase v 16, which revealed that 98% of these miRNAs had size smaller than 135nt (Additional file 7). Including most of the hypothetical precursor sequence was important for the structural characterization of the candidates (INFERNAL, RNAFold and HHMMiR). The 448 sequences extracted in this manner formed a miRNA candidate set.

All 448 candidate miRNA sequences obtained as described above were then submitted to a 5-step analysis: similarity search against miRNA sequences, false positive detection, structural search against RFAM, *ab initio* classification using HHMMiR, folding using RNAfold (version 1.8) and tabulation of the results.

Similarity search against known miRNA sequences was performed against a local miRNA database with sequences from databases designated by NRDR [27] as containing miRNAs. Sequences from 15 different databases were included [28], miRBase [29], miRNAMap [30], microRNA.org [31], Argonaute, ASRP [32], CSRDB [33], fRNAdb [34], ncRNAdb [35], NONCODE [36], RFAM [37], RNAdb [38], smiRNAdb [39], TarBase [40], UCSC Genome Browser human miRNAs [41]. Similarity search was performed using WU-BLAST v.2.2.6 with the DUST filter turned on, wordsize 6, and minimum e-value of 0.0001. Results were filtered to the following cutoff values: minimum 80% coverage of the query or of the subject sequence, 80% minimum identity. False positive detection adopted the conservative approach of excluding all candidates mapping on known exons, all candidates that matched other ncRNA types on our local database and all candidates postulated as tRNAs by tRNAScan-SE version 1.23 [42].

Structural search against RFAM version 9.1 [37] was performed using INFERNAL version 0.81 with the recommended cutoff value 25. *Ab initio* prediction with HHMMiR version 1.2 was performed using the recommended cutoff value of 0.71 MLE.

### Identification of mature miRNAs within putative precursors

In order to identify a mature miRNA sequence within putative miRNA precursors, following the analysis presented in Figure 3 candidates that showed the original read mapping to a predicted stem (at least partially) were analyzed using the MatureBayes algorithm [43]. This computational tool incorporates a Naive Bayes classifier to identify mature miRNA candidates based on sequence and secondary structure information of their miRNA putative precursors.

## Competing interests

The authors declare there are no competing interests.

## Authors’ contributions

PS: defined the research theme and designed the study, carried out and analyzed microarray experiments, integrated and interpreted data, LS: carried out secondary sequencing data analysis, assembled and selected reads for novel miRNA discovery, performed differential microRNA expression analysis and mature sequence search within precursors; PS and NT: performed small RNA library construction for sequencing; FMA: carried out cell culture and RNA extraction and helped in microarray experiments; FMK and MBM: obtained and cultivated normal keratinocytes; RM, VWF, FDN: performed clinical data analysis and selected clinical samples; ARP and AMD: planned methods for novel miRNA discovery and interpreted the results; PS and AMD: wrote the manuscript. All authors revised and approved the manuscript.

## Supplementary Material

Additional file 1 Complete set of detected mature miRNAs in the cancer cell line (SCC25) and in normal keratinocytes. Click here for file

Additional file 2 **(A) Experimentally validated targets for miR-21, miR-24 and miR-205 and (B) KEGG/Gene Ontology term enrichment analysis for these genes.** Targets were selected using the tool MicroRNA Target Filter from Ingenuity Pathway Analysis. KEGG and Gene Ontology term enrichment analysis were performed using DAVID Bioinformatics Resources ( http://david.abcc.ncifcrf.gov/home.jsp). Click here for file

Additional file 3 Differential microRNA expression between the cell line and keratinocytes. Click here for file

Additional file 4 **(A) Experimentally validated targets for microRNAs differentially expressed between keratinocytes and the cell line and (B) Gene Ontology term enrichment analysis for these genes.** Targets were selected using the tool MicroRNA Target Filter from Ingenuity Pathway Analysis. Gene Ontology term enrichment analysis was performed using DAVID Bioinformatics Resources ( http://david.abcc.ncifcrf.gov/home.jsp). Click here for file

Additional file 5 Differential gene expression (mRNA) between the cell line and keratinocytes. Click here for file

Additional file 6 **Gene Ontology term enrichment analysis for differentially expressed genes between the cell line and keratinocytes.** A: Functional analysis of genes up-regulated in SCC25. B: Functional analysis of genes up-regulated in keratinocytes. Gene Ontology term enrichment analysis was performed using DAVID Bioinformatics Resources ( http://david.abcc.ncifcrf.gov/home.jsp). Click here for file

Additional file 7 **Average lengths of known human precursor miRNAs.** The bar chart shows the length of precursor human miRNAs deposited in miRBase v. 16. Click here for file

Additional file 8 **Secondary structures of miRNA putative precursors as predicted by RNAfold.** The structures were predicted using default parameters of the computational tool. The RNAFold webserver can be found at http://rna.tbi.univie.ac.at/cgi-bin/RNAfold.cgi. Click here for file

Additional file 9 **Identification of functional parts (mature miRNAs) within putative precursor sequences using the computational tool MatureBayes.** On the precursor sequences defined in this paper as possible miRNA candidates we highlight the sequenced read (green), mature miRNA prediction at the 3’ stem (red) and mature miRNA prediction at the 5′ stem (blue). The identification of mature sequences followed the procedures in the site http://mirna.imbb.forth.gr/MatureBayes.html Click here for file

Additional file 10 Sequencing results from clinical samples. Click here for file

Additional file 11 **Fold-change between cells (SCC25 vs keratinocytes) and Clinical Samples (tumor vs tumor-free samples).** Fold-change in blue indicates overexpression in keratinocytes or tumor-free sample. Fold-change in red indicates overexpression in the cell line or in tumor sample. Click here for file
