# Supplementary material for: High-throughput sequencing of small RNA transcriptomes reveals critical biological features targeted by microRNAs in cell models used for squamous cell cancer research
Source: BMC Genomics. 2013 Oct 26;14:735. doi: 10.1186/1471-2164-14-735 (PMC3870990; doi:10.1186/1471-2164-14-735)
Supplement: Additional file 1 — Complete set of detected mature miRNAs in the cancer cell line (SCC25) and in normal keratinocytes. [file 1471-2164-14-735-S1.pdf]

Additional File 1

miRNAs expressed in keratinocytes		miRNAs expressed in SCC25	
miRNA	Read Count	miRNA	Read Count
hsa-mir-21	27710	hsa-mir-21	24303
hsa-mir-205	8003	hsa-mir-205	11564
hsa-mir-24-2	6676	hsa-mir-24-2	5567
hsa-mir-23a	4992	hsa-mir-103a-2	3459
hsa-mir-29b-2	4170	hsa-mir-23a	3449
hsa-let-7a	4085	hsa-let-7a	1962
hsa-mir-125b	2803	hsa-mir-29b-2	1218
hsa-mir-29c	2566	hsa-mir-181a-1	1100
hsa-mir-103a-2	2141	hsa-mir-17	1041
hsa-mir-451a	1941	hsa-mir-30e	1016
hsa-mir-27a	1590	hsa-mir-19b-1	1008
hsa-mir-193b	1390	hsa-mir-26a-2	823
hsa-mir-19b-1	1319	hsa-mir-451a	714
hsa-let-7b	1286	hsa-mir-29c	543
hsa-mir-26a-2	1223	hsa-mir-30c-1	510
hsa-mir-3545	1045	hsa-mir-20a	461
hsa-mir-199a	1029	hsa-mir-193b	433
hsa-mir-1	989	hsa-mir-27a	421
hsa-mir-130a	933	hsa-let-7b	410
hsa-mir-30e	872	hsa-mir-125b	372
hsa-mir-5572	779	hsa-mir-200a	370
hsa-mir-17	687	hsa-mir-181b-1	344
hsa-mir-1260a	563	hsa-mir-1	327
hsa-mir-22	542	hsa-mir-15a	310
hsa-mir-92a-1	480	hsa-mir-125a	290
hsa-mir-99a	455	hsa-mir-99a	252
hsa-mir-4315	448	hsa-mir-92a-1	243
hsa-mir-200a	447	hsa-mir-140	237
hsa-mir-140	390	hsa-mir-1260a	226
hsa-mir-135b	324	hsa-mir-301a	216
hsa-mir-320b	310	hsa-mir-320b	213
hsa-mir-214	307	hsa-mir-130a	201
hsa-mir-125a	303	hsa-mir-19a	189
hsa-mir-138-2	303	hsa-mir-200b	184
hsa-mir-181a-1	299	hsa-mir-7	172
hsa-mir-376c	290	hsa-mir-16-1	167
hsa-mir-141	270	hsa-mir-22	163
hsa-let-7e	260	hsa-let-7e	161

**hsa-mir-30c-1**	248	**hsa-mir-185**	132
**hsa-mir-101-1**	243	**hsa-mir-141**	126
**hsa-mir-19a**	231	**hsa-mir-210**	123
**hsa-mir-370**	223	**hsa-mir-199a**	113
**hsa-mir-200b**	221	**hsa-mir-4521**	111
**hsa-mir-20a**	211	**hsa-mir-181c**	106
**hsa-mir-185**	206	**hsa-mir-151b**	105
**hsa-mir-331**	199	**hsa-mir-484**	100
**hsa-let-7i**	193	**hsa-mir-101-1**	87
**hsa-mir-15a**	186	**hsa-mir-192**	86
**hsa-mir-34a**	185	**hsa-mir-370**	85
**hsa-mir-484**	166	**hsa-mir-4315**	84
**hsa-mir-181b-1**	164	**hsa-mir-5572**	80
**hsa-mir-151b**	153	**hsa-mir-100**	79
**hsa-mir-133a**	127	**hsa-mir-331**	79
**hsa-mir-148b**	114	**hsa-mir-18a**	64
**hsa-mir-429**	100	**hsa-mir-320c**	62
**hsa-mir-16-1**	93	**hsa-mir-138-2**	60
**hsa-mir-192**	92	**hsa-mir-135b**	59
**hsa-mir-376a**	90	**hsa-mir-34a**	57
**hsa-mir-301a**	87	**hsa-mir-107**	56
**hsa-mir-186**	86	**hsa-mir-10a**	55
**hsa-mir-193a**	82	**hsa-mir-155**	53
**hsa-mir-3120**	81	**hsa-mir-130b**	52
**hsa-mir-152**	79	**hsa-mir-148b**	52
**hsa-mir-200c**	75	**hsa-mir-429**	46
**hsa-mir-378i**	73	**hsa-mir-99b**	43
**hsa-mir-150**	71	**hsa-mir-133a**	40
**hsa-let-7c**	70	**hsa-mir-152**	35
**hsa-mir-423**	69	**hsa-let-7i**	34
**hsa-mir-155**	66	**hsa-mir-342**	34
**hsa-mir-195**	65	**hsa-mir-3545**	34
**hsa-mir-130b**	63	**hsa-mir-214**	33
**hsa-mir-744**	61	**hsa-mir-935**	33
**hsa-mir-320c**	58	**hsa-mir-186**	32
**hsa-mir-708**	56	**hsa-mir-324**	31
**hsa-mir-495**	54	**hsa-let-7c**	30
**hsa-mir-342**	50	**hsa-mir-181d**	29
**hsa-mir-379**	48	**hsa-mir-195**	27
**hsa-mir-18a**	46	**hsa-mir-744**	26
**hsa-mir-107**	45	**hsa-mir-150**	25
**hsa-mir-4305**	44	**hsa-mir-200c**	25
**hsa-mir-100**	43	**hsa-mir-4256**	25

**hsa-mir-146b**	42	**hsa-mir-423**	23
**hsa-mir-7**	36	**hsa-mir-132**	22
**hsa-mir-197**	35	**hsa-mir-146b**	20
**hsa-mir-409**	34	**hsa-mir-3529**	20
**hsa-mir-4256**	33	**hsa-mir-378i**	20
**hsa-mir-99b**	33	**hsa-mir-193a**	19
**hsa-mir-1307**	31	**hsa-mir-197**	18
**hsa-mir-4500**	31	**hsa-mir-1307**	17
**hsa-mir-142**	26	**hsa-mir-619**	13
**hsa-mir-34c**	26	**hsa-mir-940**	13
**hsa-mir-34b**	25	**hsa-mir-196a**	12
**hsa-mir-324**	24	**hsa-mir-3591**	12
**hsa-mir-296**	22	**hsa-mir-376c**	12
**hsa-mir-135a-2**	21	**hsa-mir-139**	11
**hsa-mir-382**	21	**hsa-mir-345**	11
**hsa-mir-4302**	21	**hsa-mir-3676**	11
**hsa-mir-4521**	21	**hsa-mir-135a-2**	10
**hsa-mir-92b**	20	**hsa-mir-142**	10
**hsa-mir-154**	19	**hsa-mir-615**	10
**hsa-mir-127**	18	**hsa-mir-625**	10
**hsa-mir-3663**	18	**hsa-mir-1180**	9
**hsa-mir-337**	17	**hsa-mir-3120**	9
**hsa-mir-181c**	16	**hsa-mir-34b**	9
**hsa-mir-3676**	16	**hsa-mir-4301**	9
**hsa-mir-3184**	15	**hsa-mir-497**	8
**hsa-mir-494**	15	**hsa-mir-4493**	7
**hsa-mir-132**	14	**hsa-mir-1301**	6
**hsa-mir-215**	14	**hsa-mir-194**	6
**hsa-mir-328**	14	**hsa-mir-3065**	6
**hsa-mir-3529**	14	**hsa-mir-330**	6
**hsa-mir-3591**	14	**hsa-mir-4700**	6
**hsa-mir-493**	14	**hsa-mir-628**	6
**hsa-mir-326**	13	**hsa-mir-92b**	6
**hsa-mir-497**	12	**hsa-mir-328**	5
**hsa-mir-629**	12	**hsa-mir-3615**	5
**hsa-mir-10a**	11	**hsa-mir-3940**	5
**hsa-mir-136**	11	**hsa-mir-4302**	5
**hsa-mir-139**	11	**hsa-mir-4724**	5
**hsa-mir-194**	11	**hsa-mir-548c**	5
**hsa-mir-345**	11	**hsa-mir-551a**	5
**hsa-mir-369**	11	**hsa-mir-1227**	4
**hsa-mir-381**	11	**hsa-mir-136**	4
**hsa-mir-627**	11	**hsa-mir-1972**	4

hsa-mir-664	10	hsa-mir-215	4
hsa-mir-1249	9	hsa-mir-3184	4
hsa-mir-1306	9	hsa-mir-34c	4
hsa-mir-454	9	hsa-mir-3663	4
hsa-mir-487a	9	hsa-mir-376a	4
hsa-mir-539	9	hsa-mir-4305	4
hsa-mir-543	9	hsa-mir-4727	4
hsa-mir-33a	8	hsa-mir-4732	4
hsa-mir-378f	8	hsa-mir-629	4
hsa-mir-433	8	hsa-mir-641	4
hsa-mir-619	8	hsa-mir-664	4
hsa-mir-642b	8	hsa-mir-127	3
hsa-mir-1180	7	hsa-mir-1287	3
hsa-mir-1260b	7	hsa-mir-129-2	3
hsa-mir-196a	7	hsa-mir-1910	3
hsa-mir-210	7	hsa-mir-2110	3
hsa-mir-330	7	hsa-mir-329	3
hsa-mir-3653	7	hsa-mir-338	3
hsa-mir-625	7	hsa-mir-4419b	3
hsa-mir-769	7	hsa-mir-4530	3
hsa-mir-1301	6	hsa-mir-454	3
hsa-mir-134	6	hsa-mir-4999	3
hsa-mir-144	6	hsa-mir-511	3
hsa-mir-181d	6	hsa-mir-515-2	3
hsa-mir-4301	6	hsa-mir-558	3
hsa-mir-762	6	hsa-mir-708	3
hsa-mir-940	6	hsa-mir-765	3
hsa-mir-187	5	hsa-mir-1249	2
hsa-mir-1910	5	hsa-mir-1254	2
hsa-mir-299	5	hsa-mir-1260b	2
hsa-mir-3065	5	hsa-mir-1278	2
hsa-mir-431	5	hsa-mir-1289-1	2
hsa-mir-4484	5	hsa-mir-1297	2
hsa-mir-4485	5	hsa-mir-1302-1	2
hsa-mir-487b	5	hsa-mir-1909	2
hsa-mir-548ar	5	hsa-mir-212	2
hsa-mir-659	5	hsa-mir-301b	2
hsa-mir-1185	4	hsa-mir-3157	2
hsa-mir-1247	4	hsa-mir-3158-2	2
hsa-mir-1290	4	hsa-mir-3160-2	2
hsa-mir-211	4	hsa-mir-3191	2
hsa-mir-3118	4	hsa-mir-3198	2
hsa-mir-329	4	hsa-mir-320e	2

**hsa-mir-338**	4	**hsa-mir-33a**	2
**hsa-mir-33b**	4	**hsa-mir-3618**	2
**hsa-mir-411**	4	**hsa-mir-3664**	2
**hsa-mir-4323**	4	**hsa-mir-3681**	2
**hsa-mir-548d-2**	4	**hsa-mir-3713**	2
**hsa-mir-935**	4	**hsa-mir-379**	2
**hsa-mir-1268a**	3	**hsa-mir-3913**	2
**hsa-mir-1297**	3	**hsa-mir-4293**	2
**hsa-mir-212**	3	**hsa-mir-4480**	2
**hsa-mir-3646**	3	**hsa-mir-4492**	2
**hsa-mir-3664**	3	**hsa-mir-4528**	2
**hsa-mir-3713**	3	**hsa-mir-4671**	2
**hsa-mir-372**	3	**hsa-mir-483**	2
**hsa-mir-422a**	3	**hsa-mir-487b**	2
**hsa-mir-515-2**	3	**hsa-mir-494**	2
**hsa-mir-548av**	3	**hsa-mir-495**	2
**hsa-mir-5694**	3	**hsa-mir-543**	2
**hsa-mir-616**	3	**hsa-mir-544a**	2
**hsa-mir-641**	3	**hsa-mir-622**	2
**hsa-mir-654**	3	**hsa-mir-762**	2
**hsa-mir-655**	3	**hsa-mir-769**	2
**hsa-mir-1227**	2	**hsa-mir-122**	1
**hsa-mir-1237**	2	**hsa-mir-1225**	1
**hsa-mir-1273g**	2	**hsa-mir-1233**	1
**hsa-mir-129-2**	2	**hsa-mir-1255b-2**	1
**hsa-mir-1304**	2	**hsa-mir-1268b**	1
**hsa-mir-190a**	2	**hsa-mir-1273f**	1
**hsa-mir-1972**	2	**hsa-mir-1273g**	1
**hsa-mir-2116**	2	**hsa-mir-1281**	1
**hsa-mir-3125**	2	**hsa-mir-1291**	1
**hsa-mir-3177**	2	**hsa-mir-1296**	1
**hsa-mir-3180**	2	**hsa-mir-1306**	1
**hsa-mir-3191**	2	**hsa-mir-144**	1
**hsa-mir-323a**	2	**hsa-mir-147b**	1
**hsa-mir-3613**	2	**hsa-mir-1538**	1
**hsa-mir-365a**	2	**hsa-mir-2116**	1
**hsa-mir-3670**	2	**hsa-mir-217**	1
**hsa-mir-3682**	2	**hsa-mir-2392**	1
**hsa-mir-377**	2	**hsa-mir-296**	1
**hsa-mir-380**	2	**hsa-mir-298**	1
**hsa-mir-4306**	2	**hsa-mir-3116-1**	1
**hsa-mir-4417**	2	**hsa-mir-3122**	1
**hsa-mir-4504**	2	**hsa-mir-3125**	1

**hsa-mir-4529**	2	**hsa-mir-3159**	1
**hsa-mir-4710**	2	**hsa-mir-3169**	1
**hsa-mir-4749**	2	**hsa-mir-3187**	1
**hsa-mir-4763**	2	**hsa-mir-3194**	1
**hsa-mir-483**	2	**hsa-mir-3612**	1
**hsa-mir-5047**	2	**hsa-mir-3620**	1
**hsa-mir-518b**	2	**hsa-mir-3646**	1
**hsa-mir-520b**	2	**hsa-mir-3648**	1
**hsa-mir-548c**	2	**hsa-mir-3653**	1
**hsa-mir-549**	2	**hsa-mir-3670**	1
**hsa-mir-551a**	2	**hsa-mir-3675**	1
**hsa-mir-558**	2	**hsa-mir-3677**	1
**hsa-mir-5580**	2	**hsa-mir-371a**	1
**hsa-mir-628**	2	**hsa-mir-378g**	1
**hsa-mir-656**	2	**hsa-mir-382**	1
**hsa-mir-9**	2	**hsa-mir-3917**	1
**hsa-mir-920**	2	**hsa-mir-3928**	1
**hsa-mir-122**	1	**hsa-mir-3973**	1
**hsa-mir-1228**	1	**hsa-mir-409**	1
**hsa-mir-1250**	1	**hsa-mir-422a**	1
**hsa-mir-1262**	1	**hsa-mir-4253**	1
**hsa-mir-1281**	1	**hsa-mir-4261**	1
**hsa-mir-1296**	1	**hsa-mir-4296**	1
**hsa-mir-1302-1**	1	**hsa-mir-4299**	1
**hsa-mir-137**	1	**hsa-mir-4300**	1
**hsa-mir-147b**	1	**hsa-mir-4306**	1
**hsa-mir-184**	1	**hsa-mir-4310**	1
**hsa-mir-1915**	1	**hsa-mir-4311**	1
**hsa-mir-203**	1	**hsa-mir-432**	1
**hsa-mir-302f**	1	**hsa-mir-4323**	1
**hsa-mir-3123**	1	**hsa-mir-4326**	1
**hsa-mir-3155a**	1	**hsa-mir-4484**	1
**hsa-mir-3157**	1	**hsa-mir-4517**	1
**hsa-mir-3160-1**	1	**hsa-mir-4520b**	1
**hsa-mir-3173**	1	**hsa-mir-4532**	1
**hsa-mir-3179**	1	**hsa-mir-4534**	1
**hsa-mir-3182**	1	**hsa-mir-4632**	1
**hsa-mir-3195**	1	**hsa-mir-4654**	1
**hsa-mir-3616**	1	**hsa-mir-4676**	1
**hsa-mir-3619**	1	**hsa-mir-4685**	1
**hsa-mir-3620**	1	**hsa-mir-4687**	1
**hsa-mir-3649**	1	**hsa-mir-4690**	1
**hsa-mir-365b**	1	**hsa-mir-4694**	1

**hsa-mir-3678**	1	**hsa-mir-4697**	1
**hsa-mir-3681**	1	**hsa-mir-4698**	1
**hsa-mir-376b**	1	**hsa-mir-4711**	1
**hsa-mir-3913**	1	**hsa-mir-4713**	1
**hsa-mir-3929**	1	**hsa-mir-4714**	1
**hsa-mir-3940**	1	**hsa-mir-4763**	1
**hsa-mir-3941**	1	**hsa-mir-4781**	1
**hsa-mir-3973**	1	**hsa-mir-5007**	1
**hsa-mir-410**	1	**hsa-mir-5009**	1
**hsa-mir-4255**	1	**hsa-mir-5100**	1
**hsa-mir-4257**	1	**hsa-mir-5190**	1
**hsa-mir-4258**	1	**hsa-mir-5192**	1
**hsa-mir-4261**	1	**hsa-mir-520d**	1
**hsa-mir-4295**	1	**hsa-mir-525**	1
**hsa-mir-4300**	1	**hsa-mir-548ar**	1
**hsa-mir-4303**	1	**hsa-mir-548d-2**	1
**hsa-mir-4309**	1	**hsa-mir-548f-1**	1
**hsa-mir-4312**	1	**hsa-mir-549**	1
**hsa-mir-4314**	1	**hsa-mir-555**	1
**hsa-mir-4319**	1	**hsa-mir-5579**	1
**hsa-mir-4320**	1	**hsa-mir-5583-1**	1
**hsa-mir-4322**	1	**hsa-mir-5583-2**	1
**hsa-mir-4419a**	1	**hsa-mir-5585**	1
**hsa-mir-4419b**	1	**hsa-mir-5695**	1
**hsa-mir-4420**	1	**hsa-mir-5699**	1
**hsa-mir-4480**	1	**hsa-mir-603**	1
**hsa-mir-4483**	1	**hsa-mir-620**	1
**hsa-mir-4488**	1	**hsa-mir-623**	1
**hsa-mir-4493**	1	**hsa-mir-642a**	1
**hsa-mir-4506**	1	**hsa-mir-645**	1
**hsa-mir-4517**	1	**hsa-mir-659**	1
**hsa-mir-4518**	1	**hsa-mir-665**	1
**hsa-mir-4520a**	1	**hsa-mir-675**	1
**hsa-mir-4666a**	1	**hsa-mir-9**	1
**hsa-mir-4687**	1	**hsa-mir-924**	1
**hsa-mir-4690**	1		
**hsa-mir-4692**	1		
**hsa-mir-4700**	1		
**hsa-mir-4701**	1		
**hsa-mir-4709**	1		
**hsa-mir-4724**	1		
**hsa-mir-4746**	1		
**hsa-mir-4765**	1		

**hsa-mir-485**	1
**hsa-mir-496**	1
**hsa-mir-4999**	1
**hsa-mir-5006**	1
**hsa-mir-5095**	1
**hsa-mir-511**	1
**hsa-mir-512**	1
**hsa-mir-516a**	1
**hsa-mir-517c**	1
**hsa-mir-518a**	1
**hsa-mir-5192**	1
**hsa-mir-520d**	1
**hsa-mir-544a**	1
**hsa-mir-548al**	1
**hsa-mir-548at**	1
**hsa-mir-548e**	1
**hsa-mir-548h-1**	1
**hsa-mir-548k**	1
**hsa-mir-548x**	1
**hsa-mir-557**	1
**hsa-mir-5571**	1
**hsa-mir-5582**	1
**hsa-mir-5589**	1
**hsa-mir-5686**	1
**hsa-mir-618**	1
**hsa-mir-620**	1
**hsa-mir-624**	1
**hsa-mir-626**	1
**hsa-mir-634**	1
**hsa-mir-637**	1
**hsa-mir-642a**	1
**hsa-mir-643**	1
**hsa-mir-675**	1
**hsa-mir-760**	1
**hsa-mir-889**	1
