# Supplementary material for: High-throughput sequencing of small RNA transcriptomes reveals critical biological features targeted by microRNAs in cell models used for squamous cell cancer research
Source: BMC Genomics. 2013 Oct 26;14:735. doi: 10.1186/1471-2164-14-735 (PMC3870990; doi:10.1186/1471-2164-14-735)
Supplement: Additional file 2 — (A) Experimentally validated targets for miR-21, miR-24 and miR-205 and (B) KEGG/Gene Ontology term enrichment analysis for these genes. Targets were selected using the tool MicroRNA Target Filter from Ingenuity Pathway Analysis. KEGG and Gene Ontology term enrichment analysis were performed using DAVID Bioinformatics Resources ( http://david.abcc.ncifcrf.gov/home.jsp). [file 1471-2164-14-735-S2.pdf]

Additional File 2A

ID	Gene Target	Source
**hsa-mir-205 (seed CCUUCAU)**	ATP1A1	miRecords
**hsa-mir-205**	DOK4	TargetScan Human,miRecords
**hsa-mir-205**	ERBB3	TargetScan Human,miRecords
**hsa-mir-205**	INPPL1	TargetScan Human,miRecords
**hsa-mir-205**	MED1	TargetScan Human,miRecords
**hsa-mir-205**	PRKCE	TargetScan Human,miRecords
**hsa-mir-205**	PTEN	TargetScan Human,miRecords
**hsa-mir-205**	TRPS1	TargetScan Human
**hsa-mir-205**	VEGFA	TargetScan Human,miRecords
**hsa-mir-205**	ZEB1	TargetScan Human,miRecords
**hsa-mir-205**	ZEB2	miRecords
**hsa-mir-24 (seed GGCUCAG)**	ACVR1B	TarBase,TargetScan Human,miRecords
**hsa-mir-24**	AURKB	miRecords
**hsa-mir-24**	BRCA1	miRecords
**hsa-mir-24**	CCNA2	miRecords
**hsa-mir-24**	CDK1	miRecords
**hsa-mir-24**	CDK4	miRecords
**hsa-mir-24**	CDKN2A	TarBase,miRecords
**hsa-mir-24**	DHFR	TarBase,miRecords
**hsa-mir-24**	E2F2	miRecords
**hsa-mir-24**	FEN1	miRecords
**hsa-mir-24**	FURIN	TargetScan Human
**hsa-mir-24**	MAP2K4	miRecords
**hsa-mir-24**	MAPK14	TarBase,TargetScan Human,miRecords
**hsa-mir-24**	MYC	TargetScan Human,miRecords
**hsa-mir-24**	NOTCH1	TarBase
**hsa-mir-24**	SMAD3	miRecords
**hsa-mir-24**	SMAD4	miRecords
**hsa-mir-24**	SMAD5	miRecords
**hsa-mir-21 (seed AGCUUAU)**	ACTA2	miRecords
**hsa-mir-21**	APAF1	miRecords
**hsa-mir-21**	BMPR2	TargetScan Human,miRecords
**hsa-mir-21**	BTG2	TargetScan Human,miRecords
**hsa-mir-21**	CDC25A	TargetScan Human,miRecords
**hsa-mir-21**	CDK6	TargetScan Human,miRecords
**hsa-mir-21**	CDKN1A	miRecords

**hsa-mir-21**	CFL2	TargetScan Human,miRecords
**hsa-mir-21**	E2F1	miRecords
**hsa-mir-21**	FAM3C	TargetScan Human,miRecords
**hsa-mir-21**	FAS	miRecords
**hsa-mir-21**	FASLG	TargetScan Human
**hsa-mir-21**	GLCCI1	TargetScan Human,miRecords
**hsa-mir-21**	HIPK3	miRecords
**hsa-mir-21**	IL6R	TargetScan Human,miRecords
**hsa-mir-21**	JAG1	TargetScan Human,miRecords
**hsa-mir-21**	LRRFIP1	miRecords
**hsa-mir-21**	MARCKS	miRecords
**hsa-mir-21**	MTAP	TargetScan Human,miRecords
**hsa-mir-21**	NFIB	TargetScan Human,miRecords
**hsa-mir-21**	PDCD4	TarBase,TargetScan Human,miRecords
**hsa-mir-21**	PELI1	TargetScan Human,miRecords
**hsa-mir-21**	PIK3R1	TargetScan Human
**hsa-mir-21**	PRRG4	TargetScan Human,miRecords
**hsa-mir-21**	PTEN	TarBase,miRecords
**hsa-mir-21**	RECK	TargetScan Human,miRecords
**hsa-mir-21**	RP2	TargetScan Human,miRecords
**hsa-mir-21**	SERPINB5	TarBase,TargetScan Human,miRecords
**hsa-mir-21**	SESN1	TargetScan Human,miRecords
**hsa-mir-21**	SLC16A10	TargetScan Human,miRecords
**hsa-mir-21**	SOCS5	miRecords
**hsa-mir-21**	SOX5	TargetScan Human,miRecords
**hsa-mir-21**	SPRY2	TargetScan Human,miRecords
**hsa-mir-21**	TAGLN	miRecords
**hsa-mir-21**	TGFBR2	TargetScan Human,miRecords
**hsa-mir-21**	TIMP3	TargetScan Human,miRecords
**hsa-mir-21**	TPM1	TarBase,miRecords

Additional File 2B

KEGG pathways and Gene Ontology term enrichment analysis of miR-21, miR-24 and miR-205 validated gene targets		
Term	Genes	FDR corrected p value
**hsa04115 p53 signaling pathway**	CDK1, CDKN1A, CDKN2A, SERPINB5, CDK6, APAF1, FAS, CDK4, SESN1, PTEN	2.63E-06
**hsa04110 Cell cycle**	E2F1, E2F2, CDK1, CDKN1A, CDKN2A, SMAD4, SMAD3, CDK6, CDK4, CCNA2, MYC, CDC25A	3.01E-06
**GO:0042127 regulation of cell proliferation**	ERBB3, TGFBR2, SMAD4, BMPR2, SMAD3, CDK6, IL6R, JAG1, ZEB1, CDK4, PTEN, SESN1, BRCA1, NOTCH1, CDKN1A, CDKN2A, BTG2, VEGFA, MYC, CCNA2, NFIB	1.17E-07
**GO:0051726 regulation of cell cycle**	E2F1, E2F2, CDK1, SMAD3, CDK6, CDK4, PTEN, PDCD4, CDC25A, BRCA1, CDKN1A, CDKN2A, MAPK14, MYC, CCNA2	2.16E-07
**GO:0042981 regulation of apoptosis**	CDK1, ERBB3, SMAD3, FASLG, IL6R, PRKCE, FURIN, PTEN, TIMP3, BRCA1, ACVR1B, NOTCH1, CDKN1A, CDKN2A, BTG2, HIPK3, VEGFA, APAF1, FAS, MYC	1.36E-06
**GO:0043067 regulation of programmed cell death**	CDK1, ERBB3, SMAD3, FASLG, IL6R, PRKCE, FURIN, PTEN, TIMP3, BRCA1, ACVR1B, NOTCH1, CDKN1A, CDKN2A, BTG2, HIPK3, VEGFA, APAF1, FAS, MYC	1.61E-06
**GO:0010941 regulation of cell death**	CDK1, ERBB3, SMAD3, FASLG, IL6R, PRKCE, FURIN, PTEN, TIMP3, BRCA1, ACVR1B, NOTCH1, CDKN1A, CDKN2A, BTG2, HIPK3, VEGFA, APAF1, FAS, MYC	1.71E-06
