# Supplementary material for: High-throughput sequencing of small RNA transcriptomes reveals critical biological features targeted by microRNAs in cell models used for squamous cell cancer research
Source: BMC Genomics. 2013 Oct 26;14:735. doi: 10.1186/1471-2164-14-735 (PMC3870990; doi:10.1186/1471-2164-14-735)
Supplement: Additional file 3 — Differential microRNA expression between the cell line and keratinocytes. [file 1471-2164-14-735-S3.pdf]

Additional File 3

miRNAs	Mostly expressed in keratinocytes	miRNAs	Mostly expressed in the cell line
**hsa-mir-409**	25.11	hsa-mir-129-2	2.03
**hsa-mir-3545**	22.70	hsa-mir-558	2.03
**hsa-mir-495**	19.94	hsa-mir-4301	2.03
**hsa-mir-376c**	17.85	hsa-mir-17	2.05
**hsa-mir-379**	17.73	hsa-mir-132	2.13
**hsa-mir-376a**	16.62	hsa-mir-103a-2	2.19
**hsa-mir-296**	16.25	hsa-mir-619	2.20
**hsa-mir-382**	15.51	hsa-mir-15a	2.26
**hsa-mir-708**	13.79	hsa-mir-196a	2.32
**hsa-mir-4305**	8.13	hsa-mir-16-1	2.43
**hsa-mir-5572**	7.19	hsa-mir-100	2.49
**hsa-mir-214**	6.87	hsa-mir-1227	2.71
**hsa-mir-199a**	6.73	hsa-mir-1302-1	2.71
**hsa-mir-1306**	6.65	hsa-mir-1972	2.71
**hsa-mir-3120**	6.65	hsa-mir-3157	2.71
**hsa-mir-125b**	5.57	hsa-mir-3681	2.71
**hsa-mir-494**	5.54	hsa-mir-3913	2.71
**hsa-mir-3653**	5.17	hsa-mir-4480	2.71
**hsa-mir-34c**	4.80	hsa-mir-544a	2.71
**hsa-mir-127**	4.43	hsa-mir-30c-1	2.78
**hsa-mir-144**	4.43	hsa-mir-181b-1	2.84
**hsa-let-7i**	4.19	hsa-mir-940	2.93
**hsa-mir-135b**	4.06	hsa-mir-20a	2.96
**hsa-mir-4315**	3.94	hsa-mir-301a	3.36
**hsa-mir-138-2**	3.73	hsa-mir-548c	3.38
**hsa-mir-4484**	3.69	hsa-mir-551a	3.38
**hsa-mir-548ar**	3.69	hsa-mir-4419b	4.06
**hsa-mir-659**	3.69	hsa-mir-4999	4.06
**hsa-mir-29c**	3.49	hsa-mir-511	4.06
**hsa-mir-130a**	3.43	hsa-mir-628	4.06
**hsa-mir-1249**	3.32	hsa-mir-181a-1	4.98
**hsa-mir-3663**	3.32	hsa-mir-7	6.47
**hsa-mir-543**	3.32	hsa-mir-181d	6.54
**hsa-mir-193a**	3.19	hsa-mir-10a	6.77
**hsa-mir-4302**	3.10	hsa-mir-3940	6.77
**hsa-mir-33a**	2.95	hsa-mir-4724	6.77
**hsa-mir-4323**	2.95	hsa-mir-4521	7.16
**hsa-mir-548d-2**	2.95	hsa-mir-4700	8.12
**hsa-mir-27a**	2.79	hsa-mir-181c	8.97
**hsa-mir-3184**	2.77	hsa-mir-4493	9.48

**hsa-mir-378i**	2.70	hsa-mir-935	11.17
**hsa-mir-1260b**	2.59	hsa-mir-210	23.79
**hsa-mir-215**	2.59		
**hsa-mir-769**	2.59		
**hsa-mir-29b-2**	2.53		
**hsa-mir-92b**	2.46		
**hsa-mir-22**	2.46		
**hsa-mir-34a**	2.40		
**hsa-mir-193b**	2.37		
**hsa-mir-133a**	2.35		
**hsa-let-7b**	2.32		
**hsa-mir-1**	2.23		
**hsa-mir-200c**	2.22		
**hsa-mir-454**	2.22		
**hsa-mir-3646**	2.22		
**hsa-mir-422a**	2.22		
**hsa-mir-423**	2.22		
**hsa-mir-629**	2.22		
**hsa-mir-762**	2.22		
**hsa-mir-150**	2.10		
**hsa-mir-328**	2.07		
**hsa-mir-101-1**	2.06		
**hsa-mir-34b**	2.05		
**hsa-mir-136**	2.03		
**hsa-mir-451a**	2.01		
