# Supplementary material for: High-throughput sequencing of small RNA transcriptomes reveals critical biological features targeted by microRNAs in cell models used for squamous cell cancer research
Source: BMC Genomics. 2013 Oct 26;14:735. doi: 10.1186/1471-2164-14-735 (PMC3870990; doi:10.1186/1471-2164-14-735)
Supplement: Additional file 4 — (A) Experimentally validated targets for microRNAs differentially expressed between keratinocytes and the cell line and (B) Gene Ontology term enrichment analysis for these genes. Targets were selected using the tool MicroRNA Target Filter from Ingenuity Pathway Analysis. Gene Ontology term enrichment analysis was performed using DAVID Bioinformatics Resources ( http://david.abcc.ncifcrf.gov/home.jsp). [file 1471-2164-14-735-S4.pdf]

Additional File 4A

ID	Gene Target	Source
**hsa-mir-1 (seed GGAAUGU)**	ABHD11	TarBase
**hsa-mir-1 (seed GGAAUGU)**	ACPL2	TarBase,TargetScan Human,miRecords
**hsa-mir-1 (seed GGAAUGU)**	ADAR	TarBase,TargetScan Human,miRecords
**hsa-mir-1 (seed GGAAUGU)**	ADPGK	TarBase,TargetScan Human
**hsa-mir-1 (seed GGAAUGU)**	AGMAT	TarBase
**hsa-mir-1 (seed GGAAUGU)**	AGRN	TarBase
**hsa-mir-1 (seed GGAAUGU)**	ANKIB1	TarBase,TargetScan Human,miRecords
**hsa-mir-1 (seed GGAAUGU)**	ANKRD29	TarBase,TargetScan Human,miRecords
**hsa-mir-1 (seed GGAAUGU)**	ANP32B	TarBase,TargetScan Human
**hsa-mir-1 (seed GGAAUGU)**	ANPEP	TarBase
**hsa-mir-1 (seed GGAAUGU)**	ANXA2	TarBase,TargetScan Human
**hsa-mir-1 (seed GGAAUGU)**	AP3B1	TarBase
**hsa-mir-1 (seed GGAAUGU)**	AP3D1	TarBase,TargetScan Human
**hsa-mir-1 (seed GGAAUGU)**	ARCN1	TarBase,TargetScan Human,miRecords
**hsa-mir-1 (seed GGAAUGU)**	ARF3	TarBase,TargetScan Human,miRecords
**hsa-mir-1 (seed GGAAUGU)**	ARF4	TarBase,miRecords
**hsa-mir-1 (seed GGAAUGU)**	ARHGAP29	TarBase,miRecords
**hsa-mir-1 (seed GGAAUGU)**	ARHGEF18	TarBase,TargetScan Human,miRecords
**hsa-mir-1 (seed GGAAUGU)**	ARID1A	TarBase
**hsa-mir-1 (seed GGAAUGU)**	ARID2	TarBase,TargetScan Human
**hsa-mir-1 (seed GGAAUGU)**	ASH2L	TarBase,TargetScan Human
**hsa-mir-1 (seed GGAAUGU)**	ATP6V0A1	TarBase
**hsa-mir-1 (seed GGAAUGU)**	ATP6V1B2	TargetScan Human,miRecords
**hsa-mir-1 (seed GGAAUGU)**	AXL	TarBase,TargetScan Human,miRecords
**hsa-mir-1 (seed GGAAUGU)**	BCKDHB	TarBase
**hsa-mir-1 (seed GGAAUGU)**	BCL2	miRecords
**hsa-mir-1 (seed GGAAUGU)**	BDNF	TarBase,TargetScan Human,miRecords
**hsa-mir-1 (seed GGAAUGU)**	BLCAP	TarBase,TargetScan Human,miRecords
**hsa-mir-1 (seed GGAAUGU)**	BRI3BP	TarBase
**hsa-mir-1 (seed GGAAUGU)**	C12orf49	TarBase,miRecords
**hsa-mir-1 (seed GGAAUGU)**	C1orf56	TarBase,miRecords
**hsa-mir-1 (seed GGAAUGU)**	C1orf96	TarBase,TargetScan

		Human,miRecords
**hsa-mir-1 (seed GGAAUGU)**	Calm1	miRecords
**hsa-mir-1 (seed GGAAUGU)**	CAND1	TarBase,miRecords
**hsa-mir-1 (seed GGAAUGU)**	CAP1	TarBase,TargetScan Human,miRecords
**hsa-mir-1 (seed GGAAUGU)**	CDCP1	TarBase
**hsa-mir-1 (seed GGAAUGU)**	CDK14	TarBase,TargetScan Human,miRecords
**hsa-mir-1 (seed GGAAUGU)**	CDK9	TargetScan Human,miRecords
**hsa-mir-1 (seed GGAAUGU)**	CERS2	TarBase,TargetScan Human,miRecords
**hsa-mir-1 (seed GGAAUGU)**	CHST11	TarBase,TargetScan Human,miRecords
**hsa-mir-1 (seed GGAAUGU)**	CHSY1	TarBase,TargetScan Human,miRecords
**hsa-mir-1 (seed GGAAUGU)**	CLCN3	TarBase,TargetScan Human,miRecords
**hsa-mir-1 (seed GGAAUGU)**	CNN3	TargetScan Human,miRecords
**hsa-mir-1 (seed GGAAUGU)**	CNOT6	TarBase,miRecords
**hsa-mir-1 (seed GGAAUGU)**	COIL	TarBase,TargetScan Human
**hsa-mir-1 (seed GGAAUGU)**	CORO1C	TarBase,TargetScan Human
**hsa-mir-1 (seed GGAAUGU)**	CPOX	TarBase
**hsa-mir-1 (seed GGAAUGU)**	CSRP1	TarBase
**hsa-mir-1 (seed GGAAUGU)**	CTSC	TarBase
**hsa-mir-1 (seed GGAAUGU)**	DDX5	TarBase,TargetScan Human,miRecords
**hsa-mir-1 (seed GGAAUGU)**	DHX15	TarBase,TargetScan Human,miRecords
**hsa-mir-1 (seed GGAAUGU)**	DNAJB1	TarBase
**hsa-mir-1 (seed GGAAUGU)**	EGFR	TarBase
**hsa-mir-1 (seed GGAAUGU)**	EHMT1	TarBase
**hsa-mir-1 (seed GGAAUGU)**	EHMT2 (includes EG:10919)	TarBase,TargetScan Human
**hsa-mir-1 (seed GGAAUGU)**	EML4	TarBase,TargetScan Human,miRecords
**hsa-mir-1 (seed GGAAUGU)**	EPB41L4B	TarBase,TargetScan Human,miRecords
**hsa-mir-1 (seed GGAAUGU)**	ESR1	TarBase,miRecords
**hsa-mir-1 (seed GGAAUGU)**	F2	TarBase
**hsa-mir-1 (seed GGAAUGU)**	FAM57A	TarBase,miRecords
**hsa-mir-1 (seed GGAAUGU)**	FAM81A	TarBase,miRecords
**hsa-mir-1 (seed GGAAUGU)**	FBLN2	TarBase,TargetScan Human,miRecords
**hsa-mir-1 (seed GGAAUGU)**	FERMT2	TarBase

**hsa-mir-1 (seed GGAAUGU)**	FOXP1	TargetScan Human,miRecords
**hsa-mir-1 (seed GGAAUGU)**	FSTL1	TarBase,miRecords
**hsa-mir-1 (seed GGAAUGU)**	G6PD	TarBase,TargetScan Human,miRecords
**hsa-mir-1 (seed GGAAUGU)**	GAK	TarBase,TargetScan Human
**hsa-mir-1 (seed GGAAUGU)**	GCFC2	TarBase,miRecords
**hsa-mir-1 (seed GGAAUGU)**	GCH1	TarBase,TargetScan Human,miRecords
**hsa-mir-1 (seed GGAAUGU)**	GJA1	TarBase,TargetScan Human,miRecords
**hsa-mir-1 (seed GGAAUGU)**	GNPDA2	TarBase,TargetScan Human,miRecords
**hsa-mir-1 (seed GGAAUGU)**	GNPNAT1	TarBase,TargetScan Human
**hsa-mir-1 (seed GGAAUGU)**	GOLGA7	TarBase
**hsa-mir-1 (seed GGAAUGU)**	GPD2	TarBase,TargetScan Human
**hsa-mir-1 (seed GGAAUGU)**	H3F3A/H3F3B	TarBase,TargetScan Human,miRecords
**hsa-mir-1 (seed GGAAUGU)**	HAND2	TarBase,TargetScan Human,miRecords
**hsa-mir-1 (seed GGAAUGU)**	HCN2	TarBase,miRecords
**hsa-mir-1 (seed GGAAUGU)**	HCN4	TarBase,miRecords
**hsa-mir-1 (seed GGAAUGU)**	HDAC4	TarBase,TargetScan Human,miRecords
**hsa-mir-1 (seed GGAAUGU)**	HIST1H3A	TarBase,miRecords
**hsa-mir-1 (seed GGAAUGU)**	HPS4	TarBase,TargetScan Human,miRecords
**hsa-mir-1 (seed GGAAUGU)**	HSPA1A/HSPA1B	TarBase,miRecords
**hsa-mir-1 (seed GGAAUGU)**	HSPD1	TarBase,TargetScan Human,miRecords
**hsa-mir-1 (seed GGAAUGU)**	IFT52	TarBase,TargetScan Human,miRecords
**hsa-mir-1 (seed GGAAUGU)**	IGF1	TargetScan Human,miRecords
**hsa-mir-1 (seed GGAAUGU)**	INPP5F	TarBase,miRecords
**hsa-mir-1 (seed GGAAUGU)**	IP6K2	TarBase,TargetScan Human,miRecords
**hsa-mir-1 (seed GGAAUGU)**	IQGAP3	TarBase
**hsa-mir-1 (seed GGAAUGU)**	IRX5	miRecords
**hsa-mir-1 (seed GGAAUGU)**	ISY1	TarBase,miRecords
**hsa-mir-1 (seed GGAAUGU)**	ITGB4	TarBase
**hsa-mir-1 (seed GGAAUGU)**	KCNE1	miRecords
**hsa-mir-1 (seed GGAAUGU)**	KCNJ2	TarBase,TargetScan Human,miRecords
**hsa-mir-1 (seed GGAAUGU)**	KCNQ1	TarBase
**hsa-mir-1 (seed GGAAUGU)**	KIAA1598	TarBase,miRecords

**hsa-mir-1 (seed GGAAUGU)**	KIF2A	TarBase,TargetScan Human,miRecords
**hsa-mir-1 (seed GGAAUGU)**	KLHDC5	TarBase,TargetScan Human,miRecords
**hsa-mir-1 (seed GGAAUGU)**	LARP4	TargetScan Human,miRecords
**hsa-mir-1 (seed GGAAUGU)**	LASP1	TarBase,TargetScan Human,miRecords
**hsa-mir-1 (seed GGAAUGU)**	LIN7C	TarBase,TargetScan Human,miRecords
**hsa-mir-1 (seed GGAAUGU)**	LRP1	TarBase
**hsa-mir-1 (seed GGAAUGU)**	LRRC8A	TarBase,TargetScan Human,miRecords
**hsa-mir-1 (seed GGAAUGU)**	LZTFL1	TarBase,miRecords
**hsa-mir-1 (seed GGAAUGU)**	MEF2A	miRecords
**hsa-mir-1 (seed GGAAUGU)**	MET	TarBase,TargetScan Human,miRecords
**hsa-mir-1 (seed GGAAUGU)**	MGC27345	TarBase,miRecords
**hsa-mir-1 (seed GGAAUGU)**	MMD	TarBase,TargetScan Human,miRecords
**hsa-mir-1 (seed GGAAUGU)**	MOV10	TarBase
**hsa-mir-1 (seed GGAAUGU)**	MRC2	TarBase
**hsa-mir-1 (seed GGAAUGU)**	MTHFD2	TarBase
**hsa-mir-1 (seed GGAAUGU)**	MTMR12	TarBase,TargetScan Human,miRecords
**hsa-mir-1 (seed GGAAUGU)**	MTX1	TarBase,TargetScan Human,miRecords
**hsa-mir-1 (seed GGAAUGU)**	MXD4	TarBase,TargetScan Human,miRecords
**hsa-mir-1 (seed GGAAUGU)**	NETO2	TarBase,TargetScan Human,miRecords
**hsa-mir-1 (seed GGAAUGU)**	NOTCH2	TarBase,TargetScan Human
**hsa-mir-1 (seed GGAAUGU)**	NOTCH3	TargetScan Human,miRecords
**hsa-mir-1 (seed GGAAUGU)**	NRP1	TarBase,TargetScan Human
**hsa-mir-1 (seed GGAAUGU)**	OAT	TarBase,TargetScan Human,miRecords
**hsa-mir-1 (seed GGAAUGU)**	OSBPL7	TarBase,TargetScan Human,miRecords
**hsa-mir-1 (seed GGAAUGU)**	PDCD4	TarBase,TargetScan Human,miRecords
**hsa-mir-1 (seed GGAAUGU)**	PDLIM7	TarBase
**hsa-mir-1 (seed GGAAUGU)**	PGM2	TarBase,TargetScan Human,miRecords
**hsa-mir-1 (seed GGAAUGU)**	PICALM	TarBase,TargetScan Human
**hsa-mir-1 (seed GGAAUGU)**	PIM1	TargetScan Human,miRecords
**hsa-mir-1 (seed GGAAUGU)**	PLEKHB2	TarBase,miRecords

**hsa-mir-1 (seed GGAAUGU)**	PLEKHG2	TarBase,miRecords
**hsa-mir-1 (seed GGAAUGU)**	PNP	TarBase,TargetScan Human,miRecords
**hsa-mir-1 (seed GGAAUGU)**	POGK	TarBase,TargetScan Human,miRecords
**hsa-mir-1 (seed GGAAUGU)**	POLA1	TarBase,TargetScan Human,miRecords
**hsa-mir-1 (seed GGAAUGU)**	POLA2	TarBase
**hsa-mir-1 (seed GGAAUGU)**	POLR2K	TarBase,TargetScan Human,miRecords
**hsa-mir-1 (seed GGAAUGU)**	POM121	miRecords
**hsa-mir-1 (seed GGAAUGU)**	POM121C	TarBase
**hsa-mir-1 (seed GGAAUGU)**	PPIB	TarBase,TargetScan Human
**hsa-mir-1 (seed GGAAUGU)**	PREX1	TarBase,TargetScan Human,miRecords
**hsa-mir-1 (seed GGAAUGU)**	PRSS21	TarBase,TargetScan Human
**hsa-mir-1 (seed GGAAUGU)**	PTBP1	TarBase,TargetScan Human
**hsa-mir-1 (seed GGAAUGU)**	PTBP2	TarBase
**hsa-mir-1 (seed GGAAUGU)**	PTMA	TarBase,TargetScan Human
**hsa-mir-1 (seed GGAAUGU)**	PTPLAD1	TarBase,TargetScan Human,miRecords
**hsa-mir-1 (seed GGAAUGU)**	PTPLB	TarBase
**hsa-mir-1 (seed GGAAUGU)**	PTPRF	TarBase,TargetScan Human
**hsa-mir-1 (seed GGAAUGU)**	PWP1	TarBase
**hsa-mir-1 (seed GGAAUGU)**	RAB11FIP2	TarBase,miRecords
**hsa-mir-1 (seed GGAAUGU)**	RABGAP1L	TarBase,miRecords
**hsa-mir-1 (seed GGAAUGU)**	RABL2A	TarBase,miRecords
**hsa-mir-1 (seed GGAAUGU)**	RABL2B	TarBase,miRecords
**hsa-mir-1 (seed GGAAUGU)**	RBM47	TarBase,TargetScan Human,miRecords
**hsa-mir-1 (seed GGAAUGU)**	RFT1	TarBase
**hsa-mir-1 (seed GGAAUGU)**	RNF138	TarBase,TargetScan Human,miRecords
**hsa-mir-1 (seed GGAAUGU)**	SAC3D1	TarBase
**hsa-mir-1 (seed GGAAUGU)**	SDC4	TarBase,miRecords
**hsa-mir-1 (seed GGAAUGU)**	SEC23IP	TarBase
**hsa-mir-1 (seed GGAAUGU)**	SERP1	TarBase,TargetScan Human,miRecords
**hsa-mir-1 (seed GGAAUGU)**	SERPINB5	TarBase,miRecords
**hsa-mir-1 (seed GGAAUGU)**	SFXN1	TarBase
**hsa-mir-1 (seed GGAAUGU)**	SH2D4A	TarBase,TargetScan Human,miRecords
**hsa-mir-1 (seed GGAAUGU)**	SH3BGRL3	TarBase,TargetScan Human
**hsa-mir-1 (seed GGAAUGU)**	SH3PXD2B	TarBase,TargetScan

		Human,miRecords
**hsa-mir-1 (seed GGAAUGU)**	SLC16A9	TarBase,miRecords
**hsa-mir-1 (seed GGAAUGU)**	SLC25A1	TarBase,TargetScan Human
**hsa-mir-1 (seed GGAAUGU)**	SLC25A22	TarBase,TargetScan Human
**hsa-mir-1 (seed GGAAUGU)**	SLC25A30	TarBase,TargetScan Human,miRecords
**hsa-mir-1 (seed GGAAUGU)**	SLC44A1	TarBase,TargetScan Human,miRecords
**hsa-mir-1 (seed GGAAUGU)**	SNX6	TarBase
**hsa-mir-1 (seed GGAAUGU)**	SRSF9	TarBase,TargetScan Human,miRecords
**hsa-mir-1 (seed GGAAUGU)**	SRXN1	TarBase,TargetScan Human,miRecords
**hsa-mir-1 (seed GGAAUGU)**	SSNA1	TarBase
**hsa-mir-1 (seed GGAAUGU)**	SYNE1	TarBase
**hsa-mir-1 (seed GGAAUGU)**	TAC1	TarBase,miRecords
**hsa-mir-1 (seed GGAAUGU)**	TAGLN2	TarBase,TargetScan Human,miRecords
**hsa-mir-1 (seed GGAAUGU)**	TDP1 (includes EG:104884)	TarBase,TargetScan Human,miRecords
**hsa-mir-1 (seed GGAAUGU)**	TH1L	TarBase,TargetScan Human,miRecords
**hsa-mir-1 (seed GGAAUGU)**	THBS1	TarBase,TargetScan Human
**hsa-mir-1 (seed GGAAUGU)**	TIMP3	TarBase,TargetScan Human,miRecords
**hsa-mir-1 (seed GGAAUGU)**	TMSB10/TMSB4X	TarBase,TargetScan Human,miRecords
**hsa-mir-1 (seed GGAAUGU)**	TNS4	TarBase,miRecords
**hsa-mir-1 (seed GGAAUGU)**	TPM1 (includes EG:396366)	TarBase
**hsa-mir-1 (seed GGAAUGU)**	TPM2	TarBase
**hsa-mir-1 (seed GGAAUGU)**	TPM3	TarBase,TargetScan Human
**hsa-mir-1 (seed GGAAUGU)**	TPM4	TarBase,TargetScan Human,miRecords
**hsa-mir-1 (seed GGAAUGU)**	TPPP	TargetScan Human,miRecords
**hsa-mir-1 (seed GGAAUGU)**	TRAPPC3	TarBase,TargetScan Human,miRecords
**hsa-mir-1 (seed GGAAUGU)**	TRIM2	TarBase,TargetScan Human,miRecords
**hsa-mir-1 (seed GGAAUGU)**	TSPAN4	TarBase,TargetScan Human,miRecords
**hsa-mir-1 (seed GGAAUGU)**	TWF1 (includes EG:19230)	TarBase,TargetScan Human,miRecords
**hsa-mir-1 (seed GGAAUGU)**	UHMK1	TarBase,TargetScan Human,miRecords

**hsa-mir-1 (seed GGAAUGU)**	UHRF1	TarBase
**hsa-mir-1 (seed GGAAUGU)**	UNC93B1	TarBase
**hsa-mir-1 (seed GGAAUGU)**	UST	TarBase,TargetScan Human,miRecords
**hsa-mir-1 (seed GGAAUGU)**	UTRN	TarBase,TargetScan Human,miRecords
**hsa-mir-1 (seed GGAAUGU)**	WDFY1	TarBase,TargetScan Human
**hsa-mir-1 (seed GGAAUGU)**	WDR11	TarBase
**hsa-mir-1 (seed GGAAUGU)**	XPNPEP3	TarBase,TargetScan Human,miRecords
**hsa-mir-1 (seed GGAAUGU)**	XPO6	TarBase,TargetScan Human,miRecords
**hsa-mir-1 (seed GGAAUGU)**	YWHAQ	TarBase
**hsa-mir-1 (seed GGAAUGU)**	ZNF264	TarBase,TargetScan Human,miRecords
**hsa-mir-125b (seed CCCUGAG)**	ABTB1	TarBase,TargetScan Human
**hsa-mir-125b (seed CCCUGAG)**	ADAMTS1	miRecords
**hsa-mir-125b (seed CCCUGAG)**	AJUBA	TarBase
**hsa-mir-125b (seed CCCUGAG)**	ANAPC16	TargetScan Human,miRecords
**hsa-mir-125b (seed CCCUGAG)**	APLN	TarBase
**hsa-mir-125b (seed CCCUGAG)**	ARID3A	TarBase,TargetScan Human
**hsa-mir-125b (seed CCCUGAG)**	ARID3B	TarBase,TargetScan Human,miRecords
**hsa-mir-125b (seed CCCUGAG)**	ATP6AP1L	miRecords
**hsa-mir-125b (seed CCCUGAG)**	B3GALT4	miRecords
**hsa-mir-125b (seed CCCUGAG)**	BAK1	TargetScan Human,miRecords
**hsa-mir-125b (seed CCCUGAG)**	BMF	TargetScan Human,miRecords
**hsa-mir-125b (seed CCCUGAG)**	BMPR1B	TargetScan Human,miRecords
**hsa-mir-125b (seed CCCUGAG)**	C9orf86	TarBase,TargetScan Human
**hsa-mir-125b (seed CCCUGAG)**	CASP6	miRecords
**hsa-mir-125b (seed CCCUGAG)**	CASP7	miRecords

**hsa-mir-125b (seed CCCUGAG)**	CBLN2	miRecords
**hsa-mir-125b (seed CCCUGAG)**	CBX7	TargetScan Human,miRecords
**hsa-mir-125b (seed CCCUGAG)**	CCR5	TargetScan Human
**hsa-mir-125b (seed CCCUGAG)**	CDC25A	miRecords
**hsa-mir-125b (seed CCCUGAG)**	CDK6	miRecords
**hsa-mir-125b (seed CCCUGAG)**	CDKN2A	miRecords
**hsa-mir-125b (seed CCCUGAG)**	CEBPG	miRecords
**hsa-mir-125b (seed CCCUGAG)**	CYP1A1	miRecords
**hsa-mir-125b (seed CCCUGAG)**	DDX19B	TarBase
**hsa-mir-125b (seed CCCUGAG)**	DICER1	TargetScan Human,miRecords
**hsa-mir-125b (seed CCCUGAG)**	DIO3	miRecords
**hsa-mir-125b (seed CCCUGAG)**	DUS1L	TarBase,TargetScan Human
**hsa-mir-125b (seed CCCUGAG)**	E2F3	TargetScan Human
**hsa-mir-125b (seed CCCUGAG)**	ELAVL1	TargetScan Human,miRecords
**hsa-mir-125b (seed CCCUGAG)**	ENTPD4	TarBase,TargetScan Human
**hsa-mir-125b (seed CCCUGAG)**	ERBB2	TargetScan Human,miRecords
**hsa-mir-125b (seed CCCUGAG)**	ERBB3	TargetScan Human,miRecords
**hsa-mir-125b (seed CCCUGAG)**	FAM19A1	miRecords
**hsa-mir-125b (seed CCCUGAG)**	GPR160	TargetScan Human,miRecords
**hsa-mir-125b (seed CCCUGAG)**	H3F3A/H3F3B	miRecords
**hsa-mir-125b (seed CCCUGAG)**	HIST1H4A (includes others)	TargetScan Human,miRecords
**hsa-mir-125b (seed CCCUGAG)**	ID1	miRecords
**hsa-mir-125b (seed CCCUGAG)**	ID2	miRecords

**hsa-mir-125b (seed CCCUGAG)**	ID3	miRecords
**hsa-mir-125b (seed CCCUGAG)**	IGFBP3	TargetScan Human,miRecords
**hsa-mir-125b (seed CCCUGAG)**	IL1RN	miRecords
**hsa-mir-125b (seed CCCUGAG)**	JARID2	miRecords
**hsa-mir-125b (seed CCCUGAG)**	KRT19 (includes EG:16669)	miRecords
**hsa-mir-125b (seed CCCUGAG)**	LIN28A	TarBase,TargetScan Human,miRecords
**hsa-mir-125b (seed CCCUGAG)**	MAN1A1	miRecords
**hsa-mir-125b (seed CCCUGAG)**	MAP2K7	TarBase,TargetScan Human
**hsa-mir-125b (seed CCCUGAG)**	ODZ2	miRecords
**hsa-mir-125b (seed CCCUGAG)**	PCDHB10	miRecords
**hsa-mir-125b (seed CCCUGAG)**	PERP	miRecords
**hsa-mir-125b (seed CCCUGAG)**	PIGR	miRecords
**hsa-mir-125b (seed CCCUGAG)**	PPT2	TarBase
**hsa-mir-125b (seed CCCUGAG)**	RBM8A	miRecords
**hsa-mir-125b (seed CCCUGAG)**	RHEBL1	TarBase
**hsa-mir-125b (seed CCCUGAG)**	SGPL1	TargetScan Human,miRecords
**hsa-mir-125b (seed CCCUGAG)**	SMO	miRecords
**hsa-mir-125b (seed CCCUGAG)**	ST18	TargetScan Human,miRecords
**hsa-mir-125b (seed CCCUGAG)**	TOR2A	TarBase,TargetScan Human
**hsa-mir-125b (seed CCCUGAG)**	TP53	TargetScan Human,miRecords
**hsa-mir-125b (seed CCCUGAG)**	TSPAN8	miRecords
**hsa-mir-125b (seed CCCUGAG)**	UBE2I	miRecords
**hsa-mir-125b (seed CCCUGAG)**	UGT2B15	miRecords

**hsa-mir-125b (seed CCCUGAG)**	UGT2B17	miRecords
**hsa-mir-125b (seed CCCUGAG)**	UGT2B28	miRecords
**hsa-mir-125b (seed CCCUGAG)**	ZNF385A	TarBase,TargetScan Human
**hsa-mir-133a (seed UUGGUCC)**	BCL2L2	TargetScan Human
**hsa-mir-133a (seed UUGGUCC)**	CASP9	TarBase,miRecords
**hsa-mir-133a (seed UUGGUCC)**	CDC42	miRecords
**hsa-mir-133a (seed UUGGUCC)**	CTGF	TargetScan Human
**hsa-mir-133a (seed UUGGUCC)**	FSCN1	TargetScan Human,miRecords
**hsa-mir-133a (seed UUGGUCC)**	HCN2	TarBase,miRecords
**hsa-mir-133a (seed UUGGUCC)**	HCN4	TarBase
**hsa-mir-133a (seed UUGGUCC)**	IGF1R	TargetScan Human,miRecords
**hsa-mir-133a (seed UUGGUCC)**	KCNE1	TarBase
**hsa-mir-133a (seed UUGGUCC)**	KCNH2	miRecords
**hsa-mir-133a (seed UUGGUCC)**	KCNQ1	miRecords
**hsa-mir-133a (seed UUGGUCC)**	KLF15	miRecords
**hsa-mir-133a (seed UUGGUCC)**	KRT7	miRecords
**hsa-mir-133a (seed UUGGUCC)**	MCL1	TargetScan Human
**hsa-mir-133a (seed UUGGUCC)**	NFATC4	miRecords
**hsa-mir-133a (seed UUGGUCC)**	PITX3	miRecords
**hsa-mir-133a (seed UUGGUCC)**	PKM	TarBase,miRecords
**hsa-mir-133a (seed UUGGUCC)**	PTBP2	TarBase,TargetScan Human,miRecords
**hsa-mir-133a (seed UUGGUCC)**	RHOA	miRecords
**hsa-mir-133a (seed UUGGUCC)**	RUNX2	miRecords

**hsa-mir-133a (seed UUGGUCC)**	SRF	TarBase,miRecords
**hsa-mir-133a (seed UUGGUCC)**	TAGLN2	TargetScan Human
**hsa-mir-133a (seed UUGGUCC)**	WHSC2	TargetScan Human,miRecords
**hsa-mir-196a (seed AGGUAGU)**	ANXA1	miRecords
**hsa-mir-196a (seed AGGUAGU)**	HOXA7	TarBase,TargetScan Human,miRecords
**hsa-mir-196a (seed AGGUAGU)**	HOXB8	TarBase,TargetScan Human,miRecords
**hsa-mir-196a (seed AGGUAGU)**	HOXC8	TarBase,TargetScan Human,miRecords
**hsa-mir-196a (seed AGGUAGU)**	HOXD8	TarBase,miRecords
**hsa-mir-196a (seed AGGUAGU)**	IKBKB	miRecords
**hsa-mir-196a (seed AGGUAGU)**	KRT5	TargetScan Human,miRecords
**hsa-mir-196a (seed AGGUAGU)**	S100A9	miRecords
**hsa-mir-196a (seed AGGUAGU)**	SPRR2C	miRecords
**hsa-mir-196a (seed AGGUAGU)**	ALOX5AP	miRecords
**hsa-mir-196a (seed AGGUAGU)**	HIF1A	TargetScan Human,miRecords
**hsa-mir-196a (seed AGGUAGU)**	LAMC2	TarBase,miRecords
**hsa-mir-196a (seed AGGUAGU)**	SET	miRecords
**hsa-mir-196a (seed AGGUAGU)**	SIRT1	TargetScan Human,miRecords
**hsa-mir-511 (seed UGUCUUU)**	TLR4	TargetScan Human
**hsa-mir-7 (seed GGAAGAC)**	EGFR	TargetScan Human,miRecords
**hsa-mir-7 (seed GGAAGAC)**	FOS	TarBase,miRecords
**hsa-mir-7 (seed GGAAGAC)**	IRS1	TargetScan Human,miRecords
**hsa-mir-7 (seed GGAAGAC)**	IRS2	TargetScan Human,miRecords
**hsa-mir-7 (seed GGAAGAC)**	PAK1	TargetScan Human,miRecords
**hsa-mir-7 (seed GGAAGAC)**	RAF1	TargetScan Human,miRecords
**hsa-mir-7 (seed GGAAGAC)**	SLC17A7	miRecords
**hsa-mir-7 (seed GGAAGAC)**	SLC3A2	TargetScan Human,miRecords
**hsa-mir-7 (seed GGAAGAC)**	SNCA	TargetScan Human,miRecords

**hsa-mir-7 (seed GGAAGAC)**	SYNE1	miRecords

Additional File 4B

Gene Ontology Term Enrichment Analysis		
**Term**	Genes	FDR Corrected p value
**GO:0042981 regulation of apoptosis**	MCL1, ERBB3, PREX1, ERBB2, SNCA, BCL2L2, TLR4, HSPA1A, TIMP3, GCH1, IGF1R, CASP6, BAK1, PLEKHG2, BDNF, CDKN2A, CASP9, BCL2, DDX19B, CHST11, RHOA, THBS1, BMF, IP6K2, EGFR, PTPRF, CEBPG, ARHGEF18, PIM1, ANXA1, TP53, ESR1, IGF1, SIRT1, NOTCH2, SMO, F2, ID3, HSPD1, IKBKB, PERP, IGFBP3	2.79E-05
**GO:0043066 negative regulation of apoptosis**	EGFR, MCL1, ERBB3, ERBB2, SNCA, PIM1, TP53, ESR1, ANXA1, IGF1, BCL2L2, HSPA1A, NOTCH2, SMO, IGF1R, BDNF, BCL2, CHST11, RHOA, HSPD1, THBS1, IKBKB	0.01
**GO:0045597 positive regulation of cell differentiation**	SH3PXD2B, PTPRF, PDLIM7, SRF, PNP, SMO, BDNF, HIF1A, ID2, BCL2, RHOA, AP3D1, BMPR1B, IGFBP3, RUNX2, AP3B1	0.02
