# Supplementary material for: High-throughput sequencing of small RNA transcriptomes reveals critical biological features targeted by microRNAs in cell models used for squamous cell cancer research
Source: BMC Genomics. 2013 Oct 26;14:735. doi: 10.1186/1471-2164-14-735 (PMC3870990; doi:10.1186/1471-2164-14-735)
Supplement: Additional file 5 — Differential gene expression (mRNA) between the cell line and keratinocytes. [file 1471-2164-14-735-S5.pdf]

Additional File 5

Gene Symbol	p-value (FDR adjusted)	FoldChange SCC25 down vs Keratinocyte	Gene Symbol	p-value (FDR adjusted)	FoldChange SCC25 up vs Keratinocyte
**KLK12**	9.06E-04	1147	C1S	3.90E-03	926
**SPRR2D**	1.65E-04	883	IGFBP3	1.17E-03	708
**CYB5A**	4.77E-03	744	LUM	4.52E-04	236
**SPRR1B**	3.10E-03	616	IFIT1	1.36E-03	212
**RPS4Y1**	6.40E-03	540	LCP1	1.08E-03	203
**KRT6B**	2.55E-03	527	GFPT2	5.25E-03	182
**SCEL**	1.73E-03	489	BST2	1.57E-03	157
**MFAP5**	2.21E-05	451	MUC4	1.31E-05	124
**RHCG**	4.96E-03	318	CTHRC1	4.93E-03	122
**KRT4**	3.67E-03	250	C1R	3.17E-03	114
**SPRR3**	3.87E-03	203	CACNG6	3.36E-03	108
**TMPRSS11D**	2.16E-03	193	IFIT2	3.43E-03	108
**CDH16**	4.77E-04	147	RSAD2	3.85E-03	106
**LEMD1**	2.01E-03	141	AIM2	2.84E-03	97
**ALOX15B**	2.60E-03	123	NID2	1.20E-04	96
**KLK11**	5.00E-03	116	ZNF114	4.61E-03	86
**M14087**	6.34E-03	112	HOXA9	1.99E-03	85
**CRNN**	1.25E-03	106	GBP5	6.30E-03	84
**COX7A1**	8.57E-04	80	KAL1	6.54E-06	76
**SPRR1A**	1.74E-03	78	HTRA3	1.68E-04	72
**SPINK5**	1.43E-03	71	INDO	1.93E-03	70
**SPRR2C**	5.70E-03	71	CXCL10	3.55E-04	70
**FNDC4**	1.07E-04	70	GBP2	3.13E-03	67
**UNQ467**	2.12E-03	69	FOXF2	7.26E-04	65
**WNT5A**	3.48E-03	64	ETV7	1.87E-04	59
**MMP10**	2.07E-05	61	IL4I1	4.94E-03	57
**IL1R2**	2.53E-03	57	OAS1	2.38E-03	55
**PI3**	5.23E-03	55	IFITM1	2.73E-03	53
**TNFRSF6B**	1.76E-03	52	FLI1	3.38E-05	51
**ATAD4**	3.17E-03	46	UCHL1	2.75E-03	50
**ZNF447**	6.27E-08	41	LYPD1	5.39E-03	50
**CD521096**	1.47E-03	40	OASL	9.88E-05	48
**ZNF83**	9.79E-04	40	IFI44L	2.00E-03	46
**PLD5**	3.99E-03	37	GBP1	2.30E-03	45
**S100A8**	4.60E-03	37	RHOBTB3	1.70E-06	45
**TFPI2**	2.01E-03	36	DLX4	5.17E-05	45
**AREG**	5.65E-03	35	CSAG2	3.09E-03	44
**PPP1R3C**	2.63E-04	34	MME	2.78E-03	43

**SLPI**	1.15E-03	33	DGKG	2.45E-05	41
**HES5**	1.69E-04	33	CMKOR1	7.79E-04	41
**HS6ST2**	8.71E-04	33	GDA	3.83E-03	40
**TMPRSS11E**	6.54E-04	32	CXCL11	3.89E-03	36
**KRT16**	3.85E-03	31	KAZALD1	3.53E-03	36
**TMEM45B**	9.46E-04	30	WISP3	2.14E-03	34
**TGM1**	1.46E-05	30	IFI44	4.22E-03	32
**ARRDC4**	3.20E-04	28	IL6	3.91E-03	31
**ZNF185**	7.45E-06	28	OLFML2A	9.59E-04	31
**NDN**	3.28E-03	27	BMF	6.42E-03	31
**CUGBP2**	2.31E-04	26	GPNMB	9.31E-04	30
**PGLYRP4**	6.09E-03	25	FLJ14167	5.54E-03	30
**ANXA9**	1.40E-04	24	BIRC4BP	1.30E-03	30
**CAPNS2**	1.97E-04	24	EGR3	2.51E-03	29
**LXN**	3.86E-03	24	CTSS	8.91E-04	28
**CSTA**	4.15E-04	23	CCL5	5.74E-03	27
**CLMN**	7.32E-04	22	TNFAIP6	5.88E-03	27
**CYP4B1**	3.89E-03	22	XCL1	6.07E-04	27
**CDKN2A**	3.90E-04	22	HIST1H1A	1.14E-03	25
**KRT13**	6.28E-03	22	WNT4	6.79E-04	24
**COL1A2**	2.75E-03	22	CRIP1	3.12E-03	24
**GRHL3**	6.32E-04	22	ISG15	3.49E-03	24
**AKR1B10**	2.22E-03	21	PCDHB2	1.05E-03	24
**EPS8L1**	7.18E-05	20	APOL3	1.83E-03	23
**FEZ1**	2.58E-03	20	FLJ20035	2.57E-03	23
**FMO2**	8.00E-05	20	PODXL	2.64E-03	23
**EMP1**	2.67E-05	20	CSAG3A	8.27E-04	23
**LOH11CR2A**	1.34E-04	19	NALP7	7.30E-04	22
**FAM84A**	1.51E-04	19	NR2E1	4.23E-03	22
**TFCP2L1**	3.46E-03	19	XCL2	6.88E-04	22
**OSBPL6**	2.32E-03	18	UGT1A6	2.44E-03	21
**PYGB**	8.15E-05	18	SIX1	9.65E-04	21
**AIM1L**	3.82E-03	16	FLJ32255	1.60E-03	20
**MAL**	4.62E-04	16	DUSP2	2.08E-03	20
**CDSN**	2.52E-03	16	SEPP1	3.91E-04	20
**TMEM139**	3.38E-03	15	DMN	3.67E-03	20
**LCE3D**	2.79E-03	15	MYLIP	4.16E-03	20
**LGALS7**	3.29E-05	14	HOXC13	4.75E-03	20
**TMTC1**	2.93E-04	14	CSF2	5.69E-04	19
**HSPC159**	3.90E-03	14	SOD2	3.19E-05	19
**FLJ10916**	1.50E-04	14	EGLN3	4.80E-03	18
**FLJ36868**	2.76E-03	14	DPYD	5.70E-03	18
**HSD17B2**	3.93E-03	14	FAM69B	1.63E-03	18

**FLJ38020**	3.52E-03	14	PDGFRL	2.82E-04	18
**MGC102966**	3.74E-05	14	SECTM1	1.38E-03	18
**MIG7**	5.38E-03	13	MEF2C	5.88E-04	17
**FABP5**	1.72E-03	13	TCERG1L	2.70E-03	17
**KRT6L**	6.19E-06	13	RUNX3	2.72E-03	17
**SBEM**	2.59E-03	12	USP18	5.16E-04	17
**SPRR2B**	8.91E-04	12	SYTL3	1.53E-03	17
**MTAC2D1**	1.62E-03	11	BTN3A3	5.90E-03	17
**LMCD1**	2.79E-03	11	IRF7	1.57E-04	17
**TP53I3**	2.07E-03	11	MMP7	1.46E-03	16
**VILL**	2.06E-03	11	SERTAD4	3.14E-03	16
**RHOV**	4.16E-03	11	TFRC	1.46E-04	16
**KRT33A**	3.79E-03	10	FLJ42709	1.21E-03	15
**KRT1**	4.92E-03	10	S73202	3.08E-03	15
**ARMCX1**	2.30E-03	10	ICAM1	2.98E-03	15
**PKP1**	2.45E-03	10	TNS3	2.27E-03	15
**ZFP42**	2.09E-03	10	SPOCK1	5.08E-04	14
**KRT24**	1.90E-03	10	TDO2	2.59E-03	13
**VLDLR**	5.70E-03	10	OBSL1	2.22E-03	13
**DIAPH2**	6.23E-05	9	FBN1	2.24E-03	13
**ERCC1**	6.18E-05	9	CFI	1.21E-05	13
**OSBPL10**	3.10E-03	9	IL8	5.17E-03	13
**SMOC1**	3.50E-04	9	SPP1	4.94E-03	13
**DHRS1**	4.42E-03	9	TNFSF10	5.83E-04	13
**TGFBR2**	4.88E-04	9	SPON2	6.09E-03	13
**ANKRD15**	2.31E-04	9	TNFAIP2	4.17E-06	13
**RARRES1**	8.49E-04	9	KYNU	3.30E-04	13
**SLC2A11**	3.71E-04	8	CSAG1	1.88E-05	13
**CSF3**	7.57E-05	8	EGR1	3.80E-03	12
**MYO5B**	4.57E-03	8	PREX1	3.29E-03	12
**FAM19A4**	2.54E-03	8	CFHR3	2.62E-03	12
**S100A4**	6.18E-03	8	CTSH	1.78E-04	12
**ZFP2**	4.07E-03	8	PROC	4.12E-03	12
**ELL3**	6.31E-04	8	CCDC3	4.21E-03	12
**RGS2**	1.72E-04	8	SLC22A4	2.23E-04	12
**MGC16121**	9.08E-04	8	PDE4A	1.29E-03	12
**CDH11**	2.72E-04	8	GPR98	5.85E-03	12
**ASAM**	1.04E-04	8	CCL28	6.97E-04	12
**ALDH1L2**	8.43E-04	8	WDR72	6.28E-03	12
**KIAA1305**	1.88E-03	8	NNMT	2.49E-03	12
**ETHE1**	2.71E-03	8	OVOS2	2.53E-03	12
**PRKAG2**	2.29E-03	8	FAM26B	3.00E-03	12
**SH2D5**	3.12E-03	8	HOXA13	1.88E-05	11

**RAB11FIP1**	3.32E-03	7	GAS1	1.46E-03	11
**DDX3Y**	4.06E-05	7	HOXA2	1.11E-03	11
**KCNK12**	1.50E-04	7	TIGD7	6.38E-05	11
**APOE**	8.47E-05	7	IGFBP1	5.92E-04	11
**FRAS1**	8.20E-04	7	MAGEA11	7.25E-05	11
**ASL**	4.96E-06	7	CDC7	5.00E-04	11
**LZTS1**	5.90E-03	7	ECM2	6.35E-03	11
**S100A14**	1.43E-03	7	FLJ22675	3.56E-03	11
**PSCA**	1.05E-03	7	MLLT11	5.14E-04	11
**PLEKHG5**	3.55E-03	7	SLC1A3	6.05E-03	11
**KRT14**	1.99E-03	7	MAGED4	2.23E-04	11
**SERPINE1**	9.92E-04	7	P2RY6	1.04E-03	10
**BTBD11**	4.10E-05	7	ID4	1.79E-03	10
**SESN3**	2.79E-03	7	FGD3	1.79E-04	10
**FUT3**	2.90E-03	7	TNFRSF9	6.56E-04	10
**MRGPRX3**	9.06E-04	7	SLITRK6	6.18E-03	10
**PTK6**	4.08E-03	7	BTN3A2	5.69E-04	10
**TMEM40**	4.39E-03	7	LRRC38	1.30E-04	10
**THBS2**	4.83E-05	7	TTK	2.74E-04	10
**NEBL**	4.01E-03	7	CFH	3.17E-03	10
**KHDRBS3**	5.56E-03	7	APOBEC3G	6.39E-04	10
**FRG1**	7.81E-04	7	SAMD11	3.03E-03	10
**ADARB1**	1.22E-03	7	TOP2A	1.05E-03	10
**FAM101A**	8.01E-04	6	SASS6	3.04E-03	9
**SOX9**	1.52E-03	6	ATF3	8.49E-04	9
**CSTB**	2.85E-05	6	CCL26	4.62E-03	9
**ACCN2**	1.29E-06	6	KRT25	4.47E-03	9
**TM4SF1**	4.93E-03	6	FLJ30064	1.83E-03	9
**STEAP2**	1.64E-04	6	KIFC1	5.58E-03	9
**QPCT**	6.17E-03	6	FAM70A	2.35E-03	9
**LRIG1**	3.50E-04	6	GPR143	9.53E-05	9
**TMEM28**	2.59E-04	6	CLSPN	2.51E-04	9
**DPYSL4**	8.96E-04	6	HHEX	3.29E-03	9
**DOCK8**	6.26E-03	6	H1F0	5.69E-05	9
**ECHDC3**	1.24E-03	6	FLJ41747	4.78E-03	9
**TMPRSS13**	6.22E-03	6	MGC16291	1.86E-03	9
**CAMK2D**	8.68E-04	6	MICB	5.37E-04	9
**FADS2**	6.23E-03	6	AMID	3.49E-04	8
**CRYAB**	1.90E-04	6	FADD	2.79E-03	8
**CTSL2**	1.36E-03	6	REC8L1	2.94E-03	8
**MGC4172**	1.64E-04	6	TAP1	2.83E-03	8
**FGF7**	3.86E-03	6	ARID5A	5.52E-04	8
**STEAP1**	3.89E-03	6	GIMAP2	4.15E-03	8

**WFDC12**	9.46E-04	5	PTN	3.23E-03	8
**DNASE1L3**	5.68E-07	5	SLC7A8	1.04E-03	8
**TMEM80**	4.98E-03	5	NEK2	5.96E-04	8
**PTPLAD2**	1.68E-04	5	MAP3K8	1.64E-03	8
**VKORC1L1**	6.36E-03	5	HLA-B	6.20E-05	8
**SERPINB1**	4.02E-03	5	KCNQ1	4.55E-03	8
**SRPX**	2.08E-03	5	USP13	1.20E-03	8
**VNN1**	3.58E-04	5	MTBP	1.24E-03	8
**RBPMS2**	6.14E-03	5	PCDH7	8.60E-04	8
**KRT2**	2.63E-07	5	CD69	8.39E-05	8
**LRAT**	8.29E-04	5	CPT1B	1.40E-04	8
**LPIN1**	2.48E-06	5	TLR2	1.36E-03	8
**SH3PXD2A**	4.54E-04	5	DKK1	1.69E-06	8
**SNCA**	6.18E-03	5	TOX	1.23E-03	8
**YIPF7**	9.09E-05	5	BHLHB3	4.97E-04	8
**ANXA2P3**	1.20E-03	5	PCDHB11	5.67E-04	8
**FSTL1**	2.61E-03	5	CD83	3.14E-06	8
**ZNF295**	5.77E-03	5	PRTFDC1	1.26E-03	8
**GSN**	4.85E-04	5	AKAP12	3.22E-05	8
**SERPINF1**	7.41E-04	5	CGI-38	4.52E-03	8
**ZNF545**	1.60E-03	5	CLDN23	2.92E-03	8
**JAM2**	1.45E-03	5	KIF18A	3.39E-03	8
**NEO1**	3.91E-03	5	TMEM45A	4.42E-04	8
**CCDC8**	6.12E-03	5	ABHD3	7.86E-05	8
**LSS**	2.34E-04	5	VEGFC	9.08E-04	7
**WIPI1**	1.46E-03	5	MEIS2	1.79E-04	7
**AP1S3**	9.66E-04	5	SNCAIP	4.29E-03	7
**TM7SF3**	2.12E-03	5	IFIH1	9.59E-04	7
**NRCAM**	5.93E-04	5	SLC1A1	1.43E-03	7
**MTL5**	9.22E-06	5	HIST1H2AL	2.59E-04	7
**CRYBB2**	4.33E-04	5	WNT10A	1.93E-04	7
**PDDC1**	4.83E-05	4	CDC25C	5.79E-04	7
**FLJ46385**	3.52E-03	4	DLG7	4.01E-03	7
**STAR**	6.48E-04	4	DHRS2	1.62E-06	7
**CMTM7**	2.82E-08	4	LMNB1	1.81E-03	7
**PLEKHG1**	1.94E-04	4	BAG2	1.49E-03	7
**TM7SF2**	1.40E-04	4	AURKA	2.06E-04	7
**TMEM154**	3.29E-03	4	MANBA	3.43E-03	7
**EPB41L4B**	6.13E-03	4	KIAA0703	5.12E-09	7
**SLC7A4**	2.22E-03	4	KRT86	2.43E-03	7
**SPEG**	4.65E-04	4	SPAG5	2.94E-03	7
**EVPL**	5.12E-04	4	WFDC2	4.71E-03	7
**ANKRD29**	2.91E-03	4	ZNF618	7.49E-05	7

**CST6**	4.74E-03	4	PLSCR4	6.40E-03	7
**KCNG1**	1.20E-06	4	DUOX2	6.33E-03	7
**TMEM16J**	4.25E-03	4	SKIL	6.22E-04	7
**ROPN1B**	4.18E-04	4	FLT3LG	6.00E-03	7
**RGS14**	4.28E-04	4	IL15RA	2.60E-04	7
**GLDC**	5.24E-03	4	CDC6	5.07E-03	7
**TGFB3**	3.77E-04	4	KIF2C	1.80E-04	7
**QPRT**	1.00E-03	4	ALDH2	1.67E-03	7
**WRB**	3.24E-03	4	FLJ45187	2.35E-04	7
**GPD1L**	1.93E-03	4	SMC2	4.18E-04	7
**CXCL5**	3.98E-03	4	MCM10	1.01E-03	7
**BSPRY**	6.04E-04	4	CENPF	5.00E-03	7
**GNG12**	1.21E-03	4	EVI1	4.60E-05	6
**GSTK1**	2.49E-03	4	KIF11	2.83E-03	6
**SATB1**	3.26E-04	4	TNIK	6.26E-03	6
**TMEM98**	3.88E-05	4	CLDN1	3.37E-03	6
**OVOL2**	3.49E-03	4	PKN3	5.93E-03	6
**KRT34**	3.16E-03	4	TTC30B	2.50E-03	6
**PSG4**	2.88E-03	4	REL	7.28E-04	6
**IDS**	1.09E-05	4	CXorf57	4.01E-04	6
**DQX1**	2.08E-03	4	CDCA5	3.46E-03	6
**CYB5R1**	5.72E-03	4	IGF2	1.84E-03	6
**TMEM160**	2.86E-03	4	MID1	9.93E-04	6
**FSTL3**	1.19E-03	4	MASTL	3.66E-05	6
**STYXL1**	2.71E-03	4	FAM46A	8.71E-04	6
**EMP3**	4.43E-04	4	WDR64	3.00E-03	6
**TGM2**	5.90E-03	4	ATG4D	3.13E-03	6
**DECR2**	3.99E-03	4	ZNF594	4.73E-03	6
**MRAS**	1.86E-03	4	EXTL2	8.50E-05	6
**SCD**	2.35E-04	4	DTX3L	1.13E-03	6
**CLTB**	1.20E-04	4	ACOT4	2.08E-04	6
**ECHDC2**	1.98E-04	4	HLA-E	5.25E-03	6
**NPR2**	3.97E-03	4	FOXA1	3.32E-03	6
**PSG1**	1.01E-04	4	ADM	3.85E-03	6
**KGFLP1**	2.46E-03	4	CCNL1	3.89E-04	6
**LEPR**	2.25E-03	4	KIF14	9.80E-04	6
**PDE4B**	3.95E-03	4	PTGS2	4.28E-04	6
**CITED4**	3.30E-03	4	PSRC1	3.89E-03	6
**GSG1**	9.21E-04	4	AURKB	5.13E-03	6
**FLJ41603**	5.38E-03	4	RDH16	1.24E-03	6
**ANKRD38**	1.97E-03	4	RAB3IL1	9.16E-04	6
**WNT5B**	5.85E-03	4	MEGF6	2.31E-03	6
**PPM1F**	5.30E-03	4	FOXL2	1.67E-03	6

**FLJ43339**	1.60E-03	4	ATAD2	1.18E-06	6
**HR**	6.43E-04	4	PHC3	2.68E-03	6
**TMEM132B**	9.05E-04	4	SMC4	1.97E-03	6
**CRELD1**	7.58E-05	4	PLK1	3.55E-04	6
**X15667**	1.09E-04	4	KLHL24	1.23E-06	6
**CHKB**	9.36E-04	3	CDCA3	8.30E-04	6
**PHYHD1**	2.13E-05	3	ZNF334	1.10E-03	6
**FGFR3**	5.21E-03	3	TMEM116	3.17E-03	6
**LLGL2**	2.00E-03	3	CCDC87	2.44E-05	6
**ETNK1**	6.80E-04	3	PRKAR2B	3.64E-03	6
**U50537**	2.82E-03	3	IFIT5	3.87E-04	6
**TP53I11**	5.92E-03	3	KLHDC7B	4.78E-04	6
**TJP3**	3.88E-04	3	SYT12	7.37E-04	6
**SLC39A2**	1.43E-04	3	KIAA0286	2.11E-04	6
**BDH1**	2.11E-03	3	UBE2C	3.07E-05	6
**TNNI3**	1.67E-04	3	PSMB8	1.19E-05	6
**MRPS25**	3.01E-03	3	FLJ20152	4.46E-03	6
**STARD4**	1.94E-06	3	CENPM	1.49E-03	6
**EPB41L5**	8.22E-04	3	NT5C3	5.61E-03	6
**WDR4**	3.77E-03	3	H2AFX	6.06E-04	6
**CACNA2D2**	5.77E-06	3	FBLN1	2.25E-03	6
**ATPBD4**	8.13E-04	3	NUSAP1	6.97E-04	5
**PLXNB1**	9.44E-04	3	NFKBIA	5.96E-03	5
**MKRN2**	5.84E-06	3	VCX	3.94E-03	5
**MYH10**	8.88E-04	3	KIAA1324L	7.43E-04	5
**TMTC4**	7.76E-04	3	C1QTNF6	5.91E-03	5
**SACS**	2.62E-04	3	SGK	6.34E-03	5
**CDH13**	1.59E-03	3	CHMP4C	3.25E-03	5
**KCNK6**	6.31E-04	3	KIAA1009	2.53E-03	5
**TMPIT**	4.99E-03	3	SALL4	5.81E-03	5
**EFHA2**	1.36E-04	3	EPSTI1	2.66E-03	5
**GDAP1**	1.11E-04	3	CG018	1.21E-03	5
**TTLL7**	3.11E-05	3	K03200	4.96E-05	5
**GADD45A**	2.01E-03	3	NRL	7.84E-04	5
**CD24**	4.13E-04	3	BIRC5	4.27E-03	5
**RHOBTB2**	2.68E-05	3	HIAT1	2.03E-03	5
**NUDT2**	5.09E-03	3	HIST1H1B	5.83E-03	5
**CCL27**	1.95E-04	3	FLJ31033	1.21E-03	5
**FBXO3**	1.88E-04	3	TMEM16A	2.32E-03	5
**FIS1**	3.18E-03	3	ASS	1.56E-05	5
**DIXDC1**	3.61E-03	3	CCDC68	6.71E-06	5
**ASAH1**	4.21E-03	3	STC2	4.07E-04	5
**PER1**	4.56E-03	3	ZNF644	3.99E-03	5

**CAPN12**	2.75E-03	3	PLXND1	1.19E-04	5
**NAV1**	6.05E-05	3	CLU	2.26E-03	5
**ATG9B**	1.22E-03	3	BRRN1	1.23E-03	5
**SERHL**	3.54E-03	3	TNNT3	4.05E-05	5
**ZFYVE21**	3.56E-04	3	MCM4	1.29E-03	5
**FLJ35024**	9.10E-04	3	LIFR	5.80E-05	5
**TUBB2A**	1.40E-04	3	TK1	1.82E-03	5
**PRRT3**	1.15E-03	3	SAA4	1.93E-03	5
**MAL2**	3.06E-03	3	LAMA4	4.05E-03	5
**DECR1**	4.29E-05	3	PTPRJ	3.11E-03	5
**FLJ20273**	3.37E-03	3	ARHGAP11A	1.93E-05	5
**PFN4**	6.30E-03	3	MRPL48	4.22E-03	5
**TRIM4**	4.37E-04	3	EBF3	4.53E-03	5
**ERCC6**	4.12E-03	3	E2F7	1.61E-03	5
**SLC35F5**	1.61E-03	3	FAM83D	1.80E-04	5
**CYBRD1**	9.47E-05	3	RECQL4	1.01E-03	5
**ZNF589**	9.26E-05	3	ATOH8	9.86E-04	5
**TBC1D14**	1.95E-03	3	NFKBIE	3.67E-03	5
**CLNS1A**	2.98E-03	3	NLC1-B	4.27E-03	5
**TCEAL5**	5.75E-03	3	PSMA5	2.77E-03	5
**PDLIM2**	2.33E-03	3	PPAP2B	4.41E-03	5
**NOL6**	1.77E-04	3	JUNB	2.55E-04	5
**OR7E24**	5.88E-03	3	NFIL3	2.56E-03	5
**PIGF**	2.48E-03	3	NINJ1	4.78E-03	5
**MTHFD2L**	4.52E-03	3	SYNJ2	2.50E-03	5
**ULBP2**	1.18E-03	3	DHRS3	4.01E-04	5
**CCDC109B**	6.04E-03	3	FAM107B	5.63E-04	5
**TCF7L1**	1.16E-03	3	RAPGEF5	1.60E-04	5
**GTF2E2**	1.80E-03	3	TCF19	5.31E-03	5
**FLJ20186**	1.90E-05	3	RBMXL1	2.01E-03	5
**NDUFA3**	1.03E-03	3	B2M	1.67E-03	5
**ADA**	1.99E-03	3	CCDC102A	3.66E-04	5
**MYO5A**	3.00E-03	3	TRMT5	5.25E-04	5
**LAMA2**	1.33E-03	3	FTHL12	5.07E-03	5
**NKX6-2**	3.17E-05	3	HSH2D	1.28E-03	5
**EVL**	1.06E-03	3	IFI16	7.11E-05	5
**DOCK7**	5.94E-03	3	UCK1	5.54E-03	5
**APEH**	1.10E-03	3	DTL	1.54E-03	5
**CAPG**	2.35E-03	3	MNS1	1.68E-04	5
**POLR2L**	4.90E-03	3	MRPL42P5	3.30E-04	5
**GCSH**	1.50E-04	3	HORMAD1	5.89E-03	5
**GRHPR**	1.52E-03	3	KCTD11	1.28E-04	5
**DLK1**	5.60E-03	3	C1RL	4.17E-04	5

**B3GNT8**	5.67E-06	3	FLJ10357	2.18E-05	5
**FAM63A**	3.65E-03	3	MYOM2	9.13E-04	5
**FGF1**	1.62E-03	3	FLJ11259	5.16E-03	5
**ACRV1**	4.72E-03	3	CEP152	1.19E-03	5
**GPR111**	3.26E-03	3	UST	1.35E-03	5
**TIMP2**	1.61E-03	3	EIF2AK2	1.61E-03	5
**SESTD1**	1.30E-03	3	RXRA	3.01E-03	5
**AK3L1**	8.75E-04	3	CDKN3	1.28E-04	5
**GUK1**	4.61E-04	3	KBTBD11	2.92E-03	5
**GM2A**	5.74E-03	3	PARP14	1.42E-03	5
**GPR125**	4.33E-05	3	BMX	3.85E-04	5
**APH1B**	1.21E-04	3	NT5C2	7.72E-07	4
**MAFB**	9.58E-04	3	LYN	3.69E-03	4
**FAM62B**	2.30E-05	3	SLCO4A1	1.85E-06	4
**FOXE1**	1.93E-03	3	CKAP2L	4.02E-03	4
**NIBP**	1.55E-03	3	HNRPAB	4.40E-03	4
**USP2**	3.89E-05	3	FAM26A	2.76E-04	4
**SOCS2**	2.51E-05	3	GDF15	1.14E-04	4
**EME2**	2.73E-04	3	ASGR1	6.33E-03	4
**EEF2**	5.37E-03	3	FEN1	1.96E-03	4
**ZAK**	2.68E-04	3	ZBTB43	3.11E-03	4
**RGS20**	1.24E-04	3	ALG10B	1.61E-04	4
**COG5**	1.75E-03	3	ZNF559	5.24E-03	4
**RUTBC3**	5.60E-03	3	GCLM	5.04E-05	4
**FAM98A**	2.54E-04	3	ADAM23	6.13E-03	4
**COTL1**	4.89E-04	3	SLC41A2	1.02E-03	4
**MAN2A1**	4.06E-03	3	SPIN	4.18E-04	4
**HYAL3**	4.30E-04	3	AMIGO2	2.44E-05	4
**PTPN18**	8.41E-04	3	KRR1	5.49E-03	4
**FLJ36748**	6.12E-04	3	CTDSP2	3.05E-03	4
**GNA15**	5.09E-04	3	ZNF484	4.28E-03	4
**Z25424**	3.37E-04	3	TMPO	1.79E-03	4
**ARPC5**	1.30E-04	3	APITD1	5.62E-03	4
**RBMS2**	2.39E-04	3	PMAIP1	4.40E-04	4
**CDC34**	1.29E-03	3	hCAP-D3	6.08E-03	4
**DUSP7**	1.67E-03	3	GTSE1	6.23E-03	4
**GALNT11**	2.85E-05	3	BBS5	4.39E-03	4
**MTMR1**	5.14E-03	3	LMO4	4.16E-05	4
**GJB6**	4.74E-03	3	MPHOSPH1	2.50E-03	4
**VPS36**	4.36E-03	3	CCNB1	6.28E-03	4
**NOLA2**	8.08E-04	3	RAB32	5.38E-04	4
**MGC5370**	4.13E-03	3	SNIP1	5.55E-03	4
**SH3GLB1**	2.30E-03	2	CBX5	1.36E-03	4

**KLF6**	4.20E-04	2	CACYBP	7.06E-05	4
**ACAT2**	1.07E-04	2	APOLD1	5.26E-03	4
**FBXL16**	4.93E-03	2	UBE2T	4.00E-03	4
**EDEM3**	6.18E-03	2	EDNRB	7.56E-04	4
**RPS27L**	2.60E-03	2	DENND3	4.61E-03	4
**GSTO2**	1.11E-03	2	CXCL2	2.99E-04	4
**MULK**	3.66E-03	2	ZNF623	1.73E-05	4
**H12329**	1.11E-06	2	SLC12A7	2.14E-03	4
**VDP**	1.91E-04	2	BIRC2	2.57E-03	4
**MTMR9**	1.35E-03	2	CENPA	8.00E-05	4
**NEIL2**	1.47E-04	2	LY75	2.71E-06	4
**CXX1**	5.32E-03	2	CAPS2	4.54E-03	4
**KLHL18**	1.54E-04	2	SHCBP1	2.93E-04	4
**RRAS**	6.38E-03	2	SLC30A7	2.88E-04	4
**ENTPD6**	2.92E-03	2	MSX2P	4.69E-04	4
**RAGE**	6.08E-03	2	SOX4	1.58E-03	4
**CDC20B**	2.78E-03	2	TMIE	1.34E-03	4
**CIB2**	5.37E-03	2	PSME1	9.19E-05	4
**STIM1**	4.08E-03	2	HPS3	1.39E-03	4
**HPCA**	2.58E-03	2	CCR7	4.14E-03	4
**SLC2A9**	4.75E-03	2	SAA2	5.01E-04	4
**OPRS1**	7.62E-04	2	KIF23	1.58E-04	4
**ELOVL1**	3.03E-03	2	CD47	1.79E-03	4
**MSRB3**	2.96E-04	2	TOPBP1	2.30E-05	4
**LYSMD1**	1.67E-04	2	HIST1H1D	3.93E-04	4
**SLC30A9**	6.07E-05	2	MT1F	3.31E-03	4
**S100A10**	2.52E-03	2	ESPL1	2.58E-03	4
**CDCP1**	3.49E-03	2	HIRIP3	1.60E-06	4
**ATP7B**	1.40E-03	2	GALNT12	2.65E-03	4
**SLC37A2**	5.55E-03	2	NAT11	1.09E-04	4
**ACO1**	1.22E-03	2	ASPM	4.11E-04	4
**FAM86B1**	3.40E-03	2	SURF1	2.79E-03	4
**RHOA**	3.45E-03	2	MAST1	2.32E-03	4
**CIRBP**	3.03E-03	2	KIAA1344	5.19E-03	4
**PGCP**	1.77E-03	2	CP110	4.76E-03	4
**ATOX1**	7.73E-04	2	SP110	3.64E-04	4
**AMPD3**	1.15E-03	2	ZWINT	8.29E-04	4
**UBXD1**	1.11E-03	2	DNTTIP2	4.56E-03	4
**AP3S1**	1.51E-03	2	RBL1	1.17E-03	4
**WNK1**	3.47E-03	2	PIGA	2.17E-03	4
**GSTA4**	7.34E-04	2	SENP5	6.06E-03	4
**RASSF1**	3.36E-03	2	POFUT1	4.88E-03	4
**KIAA1737**	1.51E-06	2	HOXD9	1.70E-04	4

**TSGA2**	3.23E-03	2	PHF14	1.99E-03	4
**PKD2**	1.15E-03	2	RAB17	2.73E-04	4
**ARHGAP8**	9.23E-04	2	CCNF	2.26E-03	4
**TSPO**	3.27E-03	2	POPDC3	3.42E-03	4
**TNFSF12**	5.87E-04	2	COL8A2	3.48E-03	4
**GJB5**	1.76E-05	2	HLA-G	8.05E-05	4
**SMAD2**	1.67E-03	2	WDR8	3.27E-03	4
**LRRC32**	5.75E-03	2	QTRT1	8.37E-05	4
**SIN3B**	1.44E-04	2	ADRB2	1.43E-03	4
**ARNTL2**	9.74E-05	2	TOR1B	2.35E-03	4
**NTN1**	5.13E-03	2	GPRC5C	7.81E-04	4
**FLJ37228**	1.79E-03	2	IFI27	5.65E-06	4
**RUFY3**	4.74E-03	2	BLVRB	3.64E-03	4
**TRAPPC5**	6.36E-03	2	WNK2	4.31E-03	4
**TMSB10**	8.19E-04	2	GBA	6.96E-05	4
**P53AIP1**	3.70E-03	2	PSMB2	1.62E-03	4
**F12**	1.05E-03	2	HELLS	1.94E-03	4
**ZNFN1A3**	5.80E-03	2	HLA-C	5.60E-05	4
**SMARCD2**	4.22E-03	2	PSMB10	1.93E-03	4
**LETM2**	2.38E-04	2	ITGA1	3.07E-03	4
**SPRED2**	1.03E-07	2	ALG10	3.17E-04	4
**SEMA4G**	6.72E-04	2	TRAM2	6.08E-03	4
**D2HGDH**	6.21E-05	2	CHST3	2.33E-03	4
**GOLGA2LY1**	2.77E-03	2	DNAJB11	2.66E-04	4
**EXOC4**	2.09E-04	2	EFEMP1	4.12E-03	4
**SALL2**	6.99E-04	2	DOLPP1	3.11E-03	4
**GIPC2**	1.32E-03	2	CDCA8	1.08E-08	4
**EIF1AY**	3.68E-05	2	KIAA0649	1.75E-04	4
**KIAA1609**	1.66E-03	2	DAPK2	1.47E-03	4
**CTSK**	1.58E-03	2	CXCL1	3.81E-03	4
**CCNG2**	4.04E-05	2	NUAK1	2.72E-03	4
**TMCO3**	2.80E-03	2	ADAM12	2.98E-04	4
**HSPB1**	6.95E-04	2	BRD3	4.71E-03	4
**ISG20L1**	3.93E-03	2	COQ10A	6.12E-03	4
**GALK1**	3.98E-03	2	CENPE	1.75E-04	4
**TNFAIP1**	2.46E-03	2	PRDM1	4.49E-03	4
**ME1**	7.13E-05	2	HIST1H4L	1.99E-06	4
**A1BG**	6.22E-03	2	MSX2	3.80E-03	4
**FBXO22**	5.32E-03	2	DCLRE1B	9.45E-04	4
**RAB5C**	5.20E-03	2	OVOL1	5.58E-05	4
**SRA1**	4.71E-04	2	CCNB2	5.47E-03	4
**CASD1**	1.89E-03	2	GTF2B	9.09E-05	4
**DNASE1L1**	2.93E-06	2	DCLRE1C	3.09E-04	4

**TNFRSF10B**	1.31E-04	2	ABCA1	5.60E-03	4
**S100A11**	6.25E-03	2	ECT2	2.56E-03	4
**LYPLA1**	5.44E-03	2	ZNF653	5.32E-03	4
**NOTCH2NL**	3.32E-03	2	IFI30	5.71E-03	4
**PLAC2**	8.90E-04	2	MAP3K2	1.06E-03	4
**RPL10L**	4.46E-03	2	ADAR	4.99E-03	4
**FZD1**	1.67E-03	2	INPP1	1.17E-03	4
**POLD4**	4.58E-03	2	TMEM118	1.69E-06	4
**POLR1E**	1.03E-04	2	CITED2	5.28E-03	4
**LAYN**	3.23E-04	2	CASP1	3.89E-03	4
**MGAT4A**	5.38E-03	2	PDCL	1.48E-03	4
**KIAA1430**	4.18E-03	2	OLFM1	5.45E-04	4
**RABGGTB**	1.07E-03	2	ZNF281	1.66E-04	4
**MGLL**	2.07E-03	2	CNNM2	3.35E-03	4
**SH2D3A**	5.26E-03	2	SGOL2	4.92E-05	4
**POLH**	2.30E-03	2	PAQR9	8.47E-04	4
**PMS2L2**	4.53E-03	2	ITPKA	6.09E-03	4
**GBA2**	2.95E-04	2	NFKB1	1.22E-03	4
**CENTD1**	7.94E-05	2	FLJ32312	5.69E-03	3
**PPIC**	2.29E-03	2	PRPF19	3.12E-03	3
**CD1A**	3.01E-03	2	FLJ11286	5.04E-05	3
**TKT**	1.82E-03	2	DDX39	2.87E-03	3
**CINP**	6.38E-03	2	HMGN2	1.06E-03	3
**PIGN**	5.71E-03	2	FAM40A	1.99E-03	3
**PQLC3**	1.23E-03	2	HBE1	3.81E-03	3
**SPIN-2**	1.02E-03	2	TUBD1	1.62E-03	3
**TEKT4**	5.65E-06	2	TROAP	6.14E-04	3
**SCFD2**	1.18E-03	2	MB	3.93E-03	3
**TAS2R3**	1.47E-03	2	KNTC1	1.75E-04	3
**SMYD2**	3.75E-03	2	RNF152	1.32E-03	3
**EPHA1**	1.74E-03	2	WHSC1L1	2.02E-05	3
**SMAP1**	1.29E-03	2	ZNF266	4.77E-04	3
**ATP6V1B2**	1.77E-03	2	MGC3032	2.50E-03	3
**ENC1**	2.65E-04	2	APOL2	5.14E-06	3
**STUB1**	4.19E-07	2	POU5F1	4.35E-03	3
**SNCG**	1.04E-04	2	ANP32B	2.56E-03	3
**SPCS1**	2.50E-03	2	SRCRB4D	1.67E-04	3
**MYO6**	2.42E-03	2	BUB1	7.19E-05	3
**ZYG11B**	2.53E-03	2	HBEGF	9.07E-09	3
**JMJD2C**	1.05E-03	2	HIST1H4J	3.86E-03	3
**ZP3**	1.93E-04	2	SQSTM1	5.14E-03	3
**EFNB3**	5.43E-03	2	KIF22	4.87E-03	3
**SRI**	3.93E-03	2	KBTBD2	2.18E-03	3

**SNX24**	1.02E-03	2	ZNF528	1.14E-03	3
**PMS2L3**	3.11E-04	2	ZBTB25	6.31E-03	3
**RECK**	3.73E-03	2	GIYD1	3.02E-03	3
**SQLE**	1.71E-04	2	DIP2C	2.09E-03	3
**HMGCR**	2.62E-03	2	RRM2	2.25E-03	3
**TEPP**	1.22E-04	2	ARHGAP21	3.68E-05	3
**KIAA1804**	2.28E-04	2	KIAA0280	9.05E-06	3
**HARS2**	2.14E-03	2	HIST1H4E	5.97E-03	3
**CCDC120**	6.07E-04	2	AADAC	1.43E-03	3
**PRSS8**	1.00E-03	2	NUPR1	7.82E-05	3
**STX12**	1.91E-03	2	PRRT2	2.67E-03	3
**CD40**	1.32E-06	2	CDC45L	5.63E-03	3
			CEP55	5.75E-03	3
			HIST1H3I	2.11E-06	3
			RHBDL3	3.46E-04	3
			FZD7	7.97E-04	3
			CRSP8	6.40E-04	3
			LANCL2	4.11E-03	3
			PLEKHK1	9.75E-05	3
			FCMD	5.60E-05	3
			FIGNL1	3.26E-03	3
			LAPTM4B	2.45E-04	3
			HIST1H4F	1.54E-03	3
			IER2	4.93E-05	3
			NFIX	1.35E-05	3
			ZSCAN5	2.70E-04	3
			PSME2	3.56E-05	3
			LMX1B	1.65E-04	3
			CNAP1	1.60E-03	3
			CABP1	3.82E-03	3
			FLJ14397	4.15E-03	3
			KIAA1450	1.50E-04	3
			MAZ	6.28E-03	3
			ZSWIM1	2.71E-03	3
			CCDC74B	6.41E-04	3
			QKI	3.52E-04	3
			PHF19	1.92E-05	3
			CASC5	5.78E-03	3
			TRIM5	1.44E-03	3
			GINS1	7.48E-06	3
			GCA	3.59E-04	3
			SPIRE1	2.32E-03	3
			WDR34	2.25E-03	3

	DNTTIP1	1.87E-05	3
	IQCC	1.48E-04	3
	EPB41L1	1.42E-04	3
	TMED1	2.93E-03	3
	PHF2	1.14E-03	3
	NPNT	4.10E-03	3
	MDC1	7.87E-04	3
	ZFAND2A	4.03E-04	3
	IL27RA	4.00E-03	3
	TMED5	5.22E-03	3
	RNF36	8.77E-06	3
	SLC31A1	3.53E-03	3
	MPHOSPH9	7.15E-07	3
	TMEM37	7.21E-05	3
	PDGFA	1.78E-03	3
	KIAA1571	2.17E-04	3
	ZNF248	3.26E-03	3
	TRAF2	2.14E-03	3
	RFP	3.31E-03	3
	IQCK	5.06E-03	3
	RAD54L	1.62E-03	3
	FTH1	7.55E-04	3
	KIF26A	2.13E-03	3
	GPR172B	3.66E-04	3
	NLC1-C	4.83E-03	3
	HNRPA0	4.41E-03	3
	SOAT1	6.37E-04	3
	RPA2	5.32E-03	3
	GTF3C5	4.58E-03	3
	DAND5	1.75E-04	3
	ROD1	3.09E-04	3
	PPM2C	1.42E-03	3
	XKR3	5.12E-04	3
	DCC1	3.15E-03	3
	ARRDC2	1.78E-04	3
	BRF2	5.42E-03	3
	FLJ39582	1.70E-03	3
	CD302	7.98E-04	3
	PARD6B	3.03E-03	3
	APOL4	4.60E-04	3
	INTS7	9.63E-04	3
	ZNF227	3.59E-03	3
	ZBED1	4.98E-04	3

	KIF15	2.88E-04	3
	ZNF175	2.57E-03	3
	SLC9A8	3.32E-04	3
	DOC2A	5.88E-03	3
	PPP6C	5.86E-03	3
	ZCD2	2.21E-03	3
	NPAT	4.41E-06	3
	PRC1	2.36E-03	3
	HIST1H4B	5.07E-03	3
	GOLPH2	3.99E-03	3
	INHBA	5.58E-03	3
	POLA	1.46E-03	3
	TDRD7	2.00E-03	3
	CDT1	1.08E-05	3
	HIST1H4D	5.52E-04	3
	NFKBIL1	6.03E-03	3
	TRPV1	2.72E-05	3
	POLA2	5.35E-04	3
	ABCD3	3.01E-03	3
	KCNMB3	1.51E-03	3
	SFPQ	4.15E-03	3
	SMPD1	5.46E-03	3
	MATK	8.43E-04	3
	KIF27	2.69E-06	3
	KIF7	9.51E-04	3
	TRIP13	1.01E-03	3
	HKR3	4.87E-03	3
	PRMT6	3.40E-03	3
	HYLS1	8.33E-04	3
	MMP23B	5.72E-03	3
	AMPD2	1.00E-03	3
	GMNN	4.24E-03	3
	HIST1H2BD	1.38E-06	3
	ZNF189	6.10E-05	3
	IER3	3.68E-04	3
	LOXL4	3.66E-03	3
	ACTL6A	5.54E-03	3
	CYP2B6	2.22E-03	3
	GALNS	4.74E-03	3
	PHF21B	3.51E-03	3
	TRIM22	5.03E-03	3
	BARD1	8.75E-07	3
	CELSR3	1.72E-04	3

	TP53BP2	7.50E-05	3
	RASIP1	7.27E-05	3
	RAD51AP1	2.73E-03	3
	GLOXD1	1.47E-04	3
	ZNF469	2.40E-04	3
	EXT1	1.99E-03	3
	PDLIM1	3.05E-03	3
	HLA-DMB	1.04E-03	3
	POLE3	3.55E-05	3
	ZNF20	5.54E-04	3
	CRSP2	4.14E-03	3
	HLXB9	1.81E-04	3
	HIST1H3F	1.84E-03	3
	GPR56	8.41E-05	3
	RNASEH2A	2.98E-03	3
	UBXD5	3.14E-03	3
	SCIN	4.46E-03	3
	TMEM15	1.18E-03	3
	RGC32	2.88E-03	3
	NNT	2.40E-03	3
	ANKRD10	2.66E-04	3
	RND3	1.68E-04	3
	ZBTB6	2.59E-03	3
	PLAT	6.42E-03	3
	CHN1	2.84E-03	3
	RAB30	2.90E-04	3
	CREB3L4	4.30E-04	3
	BRD8	1.16E-04	3
	NDUFB9	6.34E-03	3
	DNAPTP6	6.28E-03	3
	ZNF76	1.49E-03	3
	FAM55C	5.28E-03	3
	ZNF232	3.31E-06	3
	CYB5B	9.80E-06	3
	HSPA1A	2.26E-04	3
	TBC1D15	6.75E-04	3
	TESK2	6.17E-11	3
	SEPX1	9.77E-05	3
	CEI	1.92E-04	3
	TMEPAI	3.06E-04	3
	LY6K	1.11E-03	3
	USP31	4.04E-03	3
	B3GALT4	4.39E-03	3

	TG	4.93E-03	3
	RAE1	4.30E-03	3
	PTTG2	2.35E-05	3
	DNA2L	4.21E-03	3
	KCNS3	3.23E-03	3
	LEFTY1	3.00E-04	3
	GTF3C4	9.04E-05	3
	UGCG	2.46E-03	3
	FCHSD2	2.81E-03	3
	FAM102A	5.62E-04	3
	MGC14327	1.28E-04	3
	GCDH	3.40E-03	3
	ZNF292	3.20E-08	3
	TMEM39A	2.41E-03	3
	HUS1B	3.69E-03	3
	ABTB2	2.29E-04	3
	HNRPA2B1	7.14E-04	3
	GINS2	1.31E-03	3
	E2F8	4.92E-04	3
	CBFA2T2	1.40E-03	3
	MAN1B1	5.33E-04	3
	FANCM	5.84E-03	3
	MAP3K13	4.20E-03	3
	SULT4A1	6.90E-04	3
	FLJ13744	2.10E-03	3
	EDN1	9.04E-05	3
	LDB2	1.80E-03	3
	WNT3	2.78E-03	3
	INCA	2.56E-03	3
	SSNA1	5.58E-06	3
	CCL20	3.19E-03	3
	PTTG3	3.08E-04	3
	RCC2	2.52E-06	3
	TBC1D13	3.44E-04	3
	ZCCHC3	4.56E-04	3
	FLJ45248	3.19E-04	3
	TMSB4X	1.18E-03	3
	DLX2	3.16E-03	3
	CRY1	2.87E-04	3
	SP2	6.79E-04	3
	MRPL47	2.82E-03	3
	RCC1	1.13E-04	3
	LHX6	2.57E-04	3

	VAX2	3.09E-03	3
	CWF19L2	3.76E-03	3
	IL23A	5.71E-04	3
	IQCE	1.91E-03	3
	RIOK3	1.44E-04	3
	GBAP	7.73E-04	3
	CBR3	9.42E-04	3
	ZNF251	6.03E-03	3
	WDR53	2.70E-03	3
	NALP12	1.46E-03	3
	PNPT1	1.40E-03	3
	AHR	3.84E-03	3
	SNRPD1	3.79E-03	3
	KIAA1600	2.37E-04	3
	MED10	1.21E-03	3
	DZIP1	3.47E-04	3
	MGC11271	3.06E-03	3
	RASEF	1.37E-03	3
	SET	1.80E-04	3
	FAM13A1	1.01E-05	3
	RAB6A	1.46E-03	3
	PPGB	6.31E-03	3
	METTL1	8.13E-05	3
	LGP2	3.10E-04	3
	ITGAV	2.53E-03	3
	MOCOS	8.96E-05	3
	ZDHHC6	3.78E-04	3
	DERL2	9.70E-08	3
	KIAA1217	6.92E-05	2
	MGC12982	3.44E-03	2
	UBQLN1	1.75E-04	2
	NUP62CL	1.61E-03	2
	H2AFV	2.13E-03	2
	PSCD2L	4.45E-05	2
	RAD9A	1.23E-03	2
	GRIPAP1	1.54E-04	2
	HIST3H2BB	1.27E-03	2
	HBLD2	1.57E-03	2
	TRIM32	2.58E-03	2
	PAIP2	3.20E-03	2
	KIAA1706	2.60E-04	2
	ASNA1	2.62E-03	2
	WDR71	1.09E-04	2

	KIAA0495	1.83E-03	2
	CAMK1D	5.84E-03	2
	CD164	8.40E-05	2
	UCN	3.49E-03	2
	CSTF2	8.34E-04	2
	PELI2	7.23E-05	2
	SNAI1	3.50E-04	2
	SLC2A5	1.47E-04	2
	BCL3	3.07E-03	2
	CD048206	8.74E-04	2
	RFK	4.40E-03	2
	D4ST1	3.36E-03	2
	SLC39A7	3.36E-03	2
	LSG1	6.00E-04	2
	PDZD8	1.92E-03	2
	HNRPH3	4.19E-03	2
	LGMN	2.95E-03	2
	NCOA3	4.87E-04	2
	TRPC4	3.67E-03	2
	PDE10A	6.14E-03	2
	TMEM22	3.46E-03	2
	GADD45GIP1	2.54E-05	2
	HIVEP2	3.47E-03	2
	PARP1	2.43E-04	2
	PHYH	6.00E-03	2
	RWDD2	8.63E-04	2
	CASP5	5.07E-04	2
	WAC	3.33E-03	2
	MINPP1	8.09E-04	2
	HCLS1	3.25E-03	2
	PSAT1	8.60E-05	2
	MRPL11	3.44E-04	2
	RABEPK	9.82E-04	2
	HIST1H2AD	2.16E-03	2
	STARD5	5.96E-03	2
	BTN2A2	1.02E-03	2
	RNF144	2.97E-03	2
	GNS	1.80E-03	2
	SEPHS2	3.38E-03	2
	PASD1	1.79E-03	2
	KLF10	4.59E-04	2
	ACADS	1.23E-04	2
	CLPB	1.83E-03	2

	APRIN	3.32E-07	2
	ZCCHC14	5.82E-03	2
	DDOST	2.94E-03	2
	ECOP	1.70E-03	2
	ZNF12	5.88E-04	2
	RIC8B	4.69E-06	2
	NSMCE2	4.89E-03	2
	DACT1	2.65E-03	2
	BVES	4.44E-04	2
	TMEM121	2.42E-03	2
	GATM	5.72E-03	2
	FAM50B	3.24E-04	2
	KLF11	4.04E-03	2
	CDK9	5.04E-10	2
	SORD	2.73E-05	2
	BACH1	4.33E-03	2
	PSMC2	6.21E-03	2
	TRAF1	1.28E-03	2
	MPO	6.22E-03	2
	TRAPPC6A	2.79E-03	2
	DBT	1.19E-06	2
	HCFC1R1	2.34E-03	2
	ZFHX4	4.34E-03	2
	CCDC75	5.55E-03	2
	SHRM	1.96E-03	2
	TBPL1	1.30E-04	2
	JOSD1	1.12E-03	2
	RFWD2	5.53E-04	2
	FAM91A1	4.86E-03	2
	COL27A1	7.32E-04	2
	PFKFB4	2.66E-04	2
	RFC3	1.68E-03	2
	BCL2L11	4.84E-05	2
	FGFR1OP	2.27E-03	2
	CENPP	2.93E-03	2
	XKR4	4.19E-03	2
	COL4A2	3.65E-03	2
	EID-3	1.85E-05	2
	STARD13	3.41E-04	2
	CCPG1	5.10E-03	2
	RAD51	2.42E-03	2
	RBBP7	2.44E-05	2
	PPAPDC1B	7.18E-04	2

	SURF2	3.99E-04	2
	FOXP4	3.17E-04	2
	NEU1	5.14E-03	2
	NOL8	4.14E-04	2
	SLC2A6	3.49E-05	2
	ODF2	2.48E-03	2
	SLC12A8	4.73E-03	2
	MYST3	3.34E-05	2
	RBM4B	2.17E-05	2
	ANKMY2	2.09E-03	2
	CTCF	8.56E-06	2
	HSD17B6	2.21E-04	2
	ABCA2	5.70E-03	2
	SMC1A	2.20E-03	2
	MTG1	4.06E-03	2
	ROM1	6.22E-03	2
	FGF19	5.70E-05	2
	TMSL8	4.75E-06	2
	RGS3	3.10E-03	2
	RLF	4.47E-04	2
	SUV39H2	3.17E-03	2
	ALG2	1.46E-03	2
	ZNF564	7.88E-04	2
	DNAI2	2.56E-03	2
	SITPEC	1.20E-04	2
	FLJ90396	4.77E-03	2
	CHD6	5.69E-03	2
	MGA	3.77E-03	2
	FLJ20105	2.54E-03	2
	VPS11	1.63E-03	2
	GSG2	1.81E-03	2
	ELF2	3.03E-03	2
	NHN1	4.07E-03	2
	SGCZ	9.04E-04	2
	CCNJL	2.09E-03	2
	BCL2L12	4.84E-04	2
	SIVA	1.57E-06	2
	ZCCHC8	3.58E-04	2
	SFRS10	1.76E-03	2
	METTL7A	5.58E-04	2
	FBXO34	1.53E-04	2
	SLC1A5	2.29E-05	2
	ZNF271	1.90E-04	2

	PAIP1	4.23E-03	2
	PLEKHO1	6.41E-03	2
	CCDC77	1.53E-03	2
	FLJ25715	4.73E-03	2
	AK1	2.90E-05	2
	PPIL6	4.20E-03	2
	ATP1A1	5.32E-03	2
	KIAA0101	7.48E-05	2
	S79672	8.43E-05	2
	HAT1	2.74E-03	2
	LIMD2	1.61E-03	2
	ALDH3B1	4.72E-03	2
	OR2C1	3.96E-03	2
	BUB3	1.14E-06	2
	MIS12	3.18E-03	2
	SUSD1	1.35E-04	2
	TIMP4	5.36E-03	2
	CYB5R4	2.63E-03	2
	CHD3	6.64E-04	2
	CD511705	1.84E-03	2
	ZNF148	5.36E-03	2
	BCAN	1.00E-03	2
	IBRDC1	7.40E-04	2
	APCDD1L	2.64E-03	2
	CCDC99	1.12E-03	2
	NCOA5	3.72E-03	2
	KRT18	2.51E-03	2
	LSM5	1.07E-03	2
	DHX36	1.90E-03	2
	PSMD11	5.72E-03	2
	NUP107	4.41E-03	2
	ETV6	1.52E-03	2
	ESRRA	3.46E-03	2
	POLD1	2.99E-03	2
	EPHA2	1.47E-03	2
	ERP29	6.18E-03	2
	HSPC152	6.06E-05	2
	SNAPC4	3.93E-05	2
	WDHD1	5.32E-03	2
	ST14	1.94E-03	2
	BMP2	2.26E-04	2
	SLC22A3	2.63E-05	2
	PHF3	9.51E-04	2

	WDSUB1	1.07E-03	2
	DGKD	2.51E-03	2
	UNQ501	4.38E-06	2
	IFT80	3.71E-03	2
	RBM22	1.43E-03	2
	PARP6	2.87E-03	2
	FBXL6	2.85E-03	2
	PRKAA2	2.89E-03	2
	KIF24	1.86E-03	2
	MYST4	2.12E-03	2
	LFNG	1.08E-03	2
	COBRA1	1.37E-03	2
	ORC3L	5.48E-05	2
	NUDT1	5.06E-03	2
	ALG14	3.17E-03	2
	PLP2	7.47E-04	2
	LRP16	3.17E-03	2
	ADCY1	4.60E-03	2
	HRSP12	9.50E-04	2
	TRMT1	2.43E-05	2
	TAF11	2.17E-04	2
	P2RX5	1.57E-03	2
	RIF1	1.67E-03	2
	HDAC1	5.82E-03	2
	HSPA5	4.81E-03	2
	KIAA1505	4.34E-03	2
	MGC4562	6.14E-03	2
	RAPGEF1	5.10E-03	2
	IPMK	1.85E-05	2
	KIAA0841	3.50E-03	2
	AGPAT2	2.91E-03	2
	DHX16	6.21E-03	2
	FTSJ2	1.32E-05	2
	DNAH17	8.02E-04	2
	PIGX	2.63E-03	2
	DMRT3	6.36E-03	2
	GOLGA2L1	3.84E-03	2
