# Supplementary material for: High-throughput sequencing of small RNA transcriptomes reveals critical biological features targeted by microRNAs in cell models used for squamous cell cancer research
Source: BMC Genomics. 2013 Oct 26;14:735. doi: 10.1186/1471-2164-14-735 (PMC3870990; doi:10.1186/1471-2164-14-735)
Supplement: Additional file 6 — Gene Ontology term enrichment analysis for differentially expressed genes between the cell line and keratinocytes. A: Functional analysis of genes up-regulated in SCC25. B: Functional analysis of genes up-regulated in keratinocytes. Gene Ontology term enrichment analysis was performed using DAVID Bioinformatics Resources ( http://david.abcc.ncifcrf.gov/home.jsp). [file 1471-2164-14-735-S6.pdf]

Additional File 6A

Gene Ontology Term Enrichment Analysis		
**Term**	Genes	FDR Corrected p value
**GO:0000278 mitotic cell cycle**	KIF23, KIF22, KIFC1, PRC1, KNTC1, TTK, PTTG2, AURKA, AURKB, GTSE1, FAM83D, KIF2C, CDCA8, MTBP, CDCA5, ASPM, CDCA3, CDC7, CDC6, KIF11, CCNF, KIF15, NUSAP1, ESPL1, UBE2C, INHBA, PSME1, RCC2, PSMA5, PSME2, SPAG5, ZWINT, HORMAD1, PSMB10, PPP6C, CCDC99, NEK2, CEP55, RCC1, KIAA1009, CENPA, PSMB2, NPAT, BUB1, BUB3, HELLS, KIF18A, CENPF, BIRC5, CENPE, CDKN3, CDC25C, SMC2, MIS12, PSMB8, SMC4, CCNB1, MPHOSPH9, CCNB2, PSMD11, PLK1, POLD1, PSMC2, ID4, SMC1A	4.09E-15
**GO:0007049 cell cycle**	KIFC1, PRC1, KNTC1, TTK, AURKA, PTTG2, AURKB, CDCA8, MAP3K8, H2AFX, CDCA5, ASPM, CDCA3, SGOL2, RBL1, ESPL1, AHR, HHEX, KRT18, RCC2, PSMA5, SPAG5, ZWINT, CCDC99, NEK2, RCC1, KIAA1009, GADD45GIP1, OVOL1, PSMB2, MNS1, LFNG, HELLS, PARD6B, CCPG1, TP53BP2, AK1, GAS1, RAD54L, PSMB8, SUV39H2, PLK1, PSMC2, POLD1, SMC1A, KCTD11, KIF23, KIF22, CLSPN, E2F7, E2F8, SENP5, GTSE1, CDT1, FAM83D, KIF2C, MTBP, CDC7, CDC6, KIF11, CCNF, KIF15, NUSAP1, UBE2C, RAD51, INHBA, SASS6, PSME1, RIF1, PSME2, HORMAD1, PSMB10, PPP6C, CEP55, CENPA, MDC1, NPAT, BUB1, BUB3, TRIP13, IL8, GMNN, PSRC1, KIF18A, CENPF, BIRC5, CENPE, CP110, CDKN3, CDC25C, SMC2, MIS12, GSG2, SMC4, CCNB1, MPHOSPH9, CCNB2, PSMD11, ID4, BARD1	8.22E-15
**GO:0000279 M phase**	KIF23, KIF22, KIFC1, PRC1, KNTC1, TTK, PTTG2, AURKA, AURKB, FAM83D, KIF2C, CDCA8, H2AFX, CDCA5, ASPM, CDCA3, CDC6, KIF11, SGOL2, CCNF, KIF15, NUSAP1, ESPL1, UBE2C, RAD51, RCC2, SPAG5, ZWINT, HORMAD1, CCDC99, NEK2, CEP55, RCC1, KIAA1009, OVOL1, BUB1, MNS1, LFNG, BUB3, HELLS, TRIP13, KIF18A, CENPF, BIRC5, CENPE, CDC25C, RAD54L, SMC2, MIS12, SUV39H2, SMC4, CCNB1, MPHOSPH9, CCNB2, PLK1, SMC1A	6.59E-12
**GO:0007067 mitosis**	KIF23, KIF22, KIFC1, CCDC99, NEK2, KNTC1, PTTG2, AURKA, AURKB, CEP55, RCC1, KIAA1009, FAM83D, KIF2C, CDCA8, BUB1, CDCA5, HELLS, ASPM, BUB3, CDCA3, CDC6, KIF11, KIF15, CCNF, KIF18A, CENPF, NUSAP1, BIRC5, CENPE, ESPL1, CDC25C, UBE2C, SMC2, MIS12, SMC4, CCNB1, CCNB2, RCC2, SPAG5, PLK1, ZWINT, HORMAD1, SMC1A	2.74E-11
**GO:0007059 chromosome segregation**	KIFC1, CCDC99, NEK2, SGOL2, KIF18A, CENPF, NUSAP1, CTCF, PTTG2, CENPE, BIRC5, ESPL1, SMC2, MIS12, SMC4, RIOK3, ZWINT, SMC1A, CDCA5, TOP2A, BUB3	4.72E-06
**GO:0051301 cell division**	KIF23, KIFC1, PRC1, NEK2, KNTC1, PTTG2, CEP55, AURKB, SENP5, RCC1, KIAA1009, FAM83D, CDCA8, BUB1, CDCA5, ASPM, HELLS, CDCA3, CDC7, PARD6B, CDC6, KIF11, SGOL2, CCNF, CENPF, NUSAP1, BIRC5, CENPE, ESPL1, CDC25C, UBE2C, SMC2, MIS12, SMC4, CCNB1, CCNB2, RCC2, SPAG5, PLK1, ZWINT, SMC1A	2.43E-05

**GO:0051276 chromosome organization**	KIFC1, HMGN2, PTTG2, CTCF, CBX5, CDCA8, H2AFV, PRMT6, HIRIP3, H2AFX, HIST3H2BB, TOP2A, CDCA5, MYST4, MYST3, BRD8, H1F0, HIST1H1D, HIST1H1B, SGOL2, HIST1H1A, RBL1, NUSAP1, ESPL1, RBBP7, DCLRE1C, ZWINT, CCDC99, HIST1H4L, NEK2, HIST1H2AD, HAT1, SET, CENPA, HIST1H4B, HIST1H4E, HIST1H4F, ACTL6A, HIST1H4D, CHD6, HIST1H4J, BUB3, HELLS, CHD3, HIST1H2BD, KIF18A, CENPF, CENPE, RAD54L, SMC2, GSG2, MIS12, SUV39H2, SMC4, HDAC1, WHSC1L1, HIST1H3F, VCX, SMC1A, PARP1, HIST1H2AL, HIST1H3I	8.30E-05
**GO:0006323 DNA packaging**	HIST1H4L, HIST1H2AD, HAT1, CTCF, SET, H2AFV, HIST1H4B, CENPA, HIST1H4E, HIST1H4F, H2AFX, HIST1H4D, HIST3H2BB, CDCA5, TOP2A, HIST1H4J, MYST4, HELLS, MYST3, H1F0, HIST1H1D, HIST1H2BD, HIST1H1B, HIST1H1A, NUSAP1, SMC2, SMC4, HIST1H3F, HIST1H2AL, HIST1H3I	1.62E-04
**GO:0000070 mitotic sister chromatid segregation**	KIFC1, CCDC99, NEK2, KIF18A, NUSAP1, CENPE, ESPL1, SMC2, SMC4, ZWINT, SMC1A, CDCA5, BUB3	2.02E-04
**GO:0006259 DNA metabolic process**	KIF22, CLSPN, ZNF12, PTTG2, CTCF, MCM10, CDT1, FANCM, PRMT6, NSMCE2, H2AFX, CRY1, TOP2A, CDC7, CDC6, NUDT1, DTL, NOL8, GIYD1, TOPBP1, RAD9A, RBBP7, RNASEH2A, MCM4, RAD51, DCLRE1C, RFC3, DCLRE1B, RRM2, NFIX, POLA2, TK1, PRPF19, RPA2, SET, MDC1, POLE3, HELLS, FEN1, TRIP13, RECQL4, GINS1, GINS2, FOXL2, RAD51AP1, CENPF, CDC25C, RAD54L, SOD2, ORC3L, POLD1, SFPQ, SMC1A, PARP1, BARD1	3.39E-04
**GO:0007017 microtubule-based process**	KIF23, KIFC1, KIF22, CCDC99, KIF24, PRC1, KIF27, NEK2, DNAH17, UCHL1, TTK, AURKA, RCC1, GTSE1, KIF2C, KIF7, CENPA, KIF14, KIF11, KIF15, PSRC1, KIF18A, NUSAP1, CENPE, ESPL1, CP110, MID1, UBE2C, SASS6, SPAG5, ZWINT, FGFR1OP, TUBD1, KIF26A, SMC1A	3.75E-04

Additional File 6B

Gene Ontology Term Enrichment Analysis		
Term	Genes	FDR Corrected p value
**GO:0008544 epidermis development**	FRAS1, FGF7, LCE3D, SOX9, CDSN, SPINK5, CDKN2A, SPRR2C, SPRR2D, SPRR2B, TGM1, KRT1, KRT2, KRT13, GJB5, GRHL3, KRT34, SCEL, EVPL, CST6, KRT16, SPRR1A, SPRR1B, KRT14, COL1A2, FOXE1, SPRR3, CSTA, KGFLP1, EMP1, FABP5	3.10E-12
**GO:0030855 epithelial cell differentiation**	TFCP2L1, LCE3D, FZD1, SPINK5, CDSN, SCEL, EVPL, RHCG, SPRR2C, SPRR1A, SPRR2D, SPRR1B, KRT14, SPRR2B, TGM1, SPRR3, KRT2, KRT4, CSTA, EMP1	8.53E-06

**GO:0030216 keratinocyte differentiation**	LCE3D, CDSN, SCEL, EVPL, SPRR2C, SPRR2D, SPRR1A, SPRR1B, SPRR2B, TGM1, SPRR3, KRT2, CSTA	2.95E-04
**GO:0060429 epithelium development**	FRAS1, TFCP2L1, LCE3D, FZD1, SPINK5, CDSN, SCEL, EVPL, RHCG, SPRR2C, OVOL2, SPRR1A, SPRR2D, SPRR1B, KRT14, SPRR2B, TGM1, SPRR3, KRT2, CSTA, KRT4, EMP1	0.001629085
**GO:0031424 keratinization**	EVPL, SPRR2C, SPRR2D, SPRR1A, SPRR1B, LCE3D, TGM1, SPRR2B, SPRR3, KRT2	0.00306369
