# Supplementary figures and images for: High-throughput sequencing of small RNA transcriptomes reveals critical biological features targeted by microRNAs in cell models used for squamous cell cancer research
Source: BMC Genomics. 2013 Oct 26;14:735. doi: 10.1186/1471-2164-14-735 (PMC3870990; doi:10.1186/1471-2164-14-735)

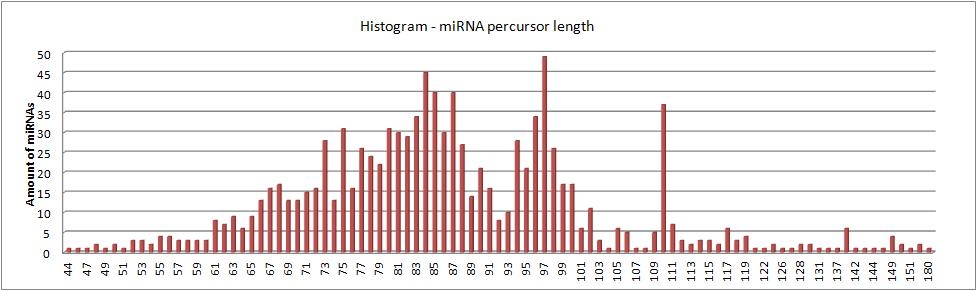

Supplement: Additional file 7 — Average lengths of known human precursor miRNAs. The bar chart shows the length of precursor human miRNAs deposited in miRBase v. 16. [file 1471-2164-14-735-S7.jpeg]

Cand1

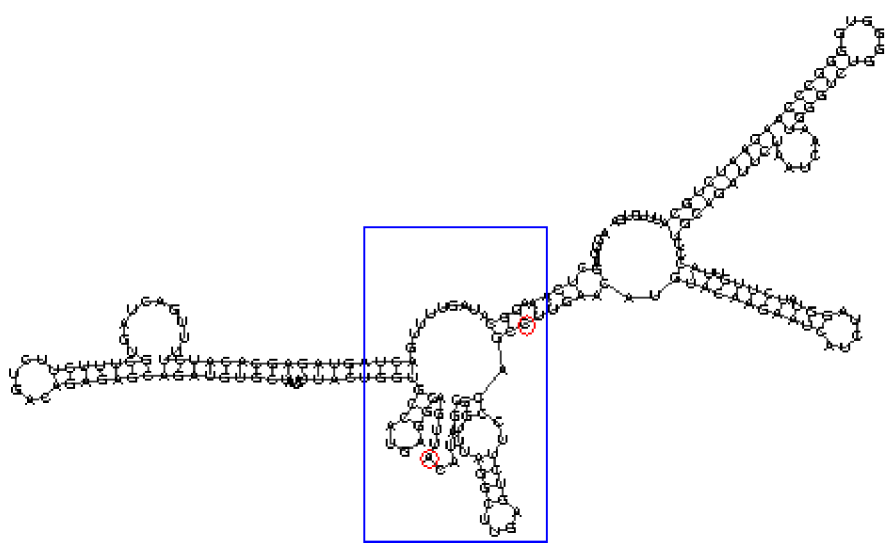

Cand2

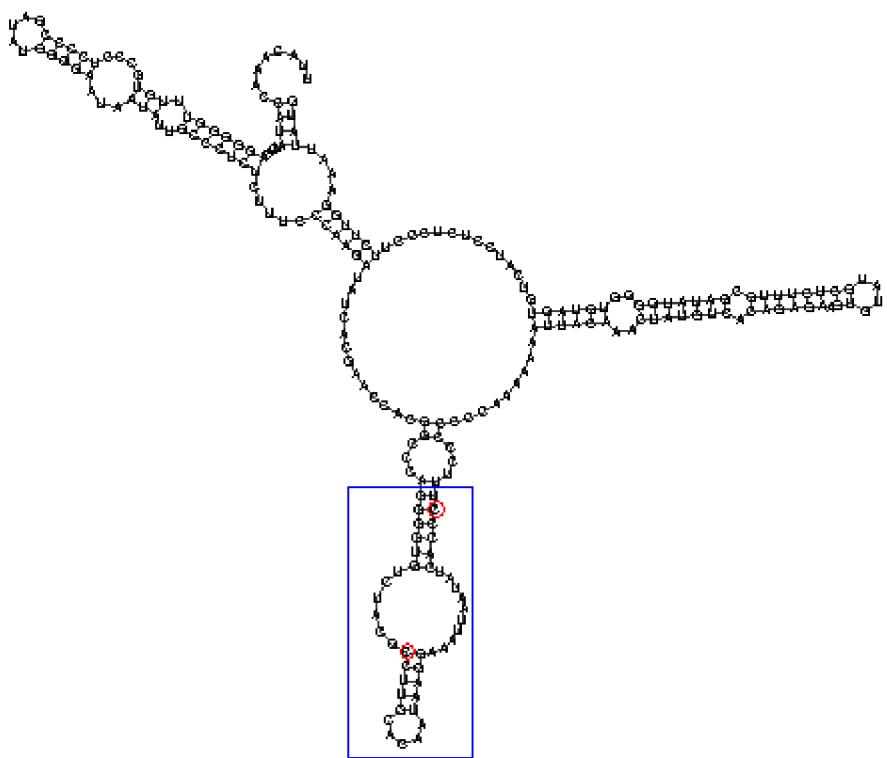

Cand3

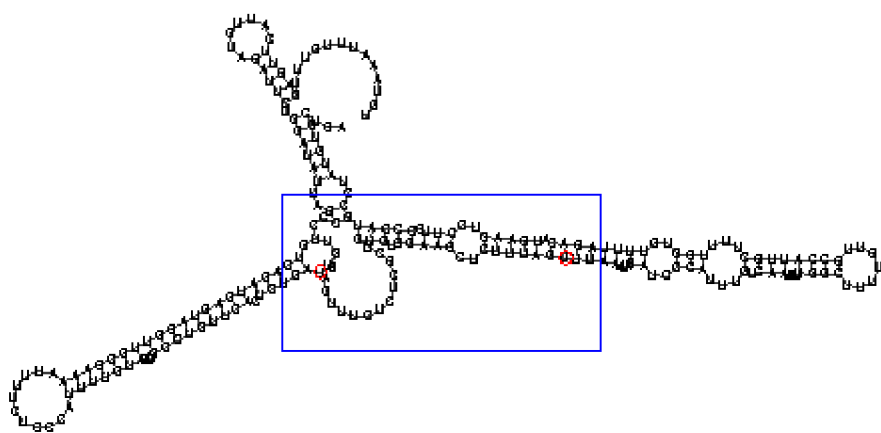

Cand4

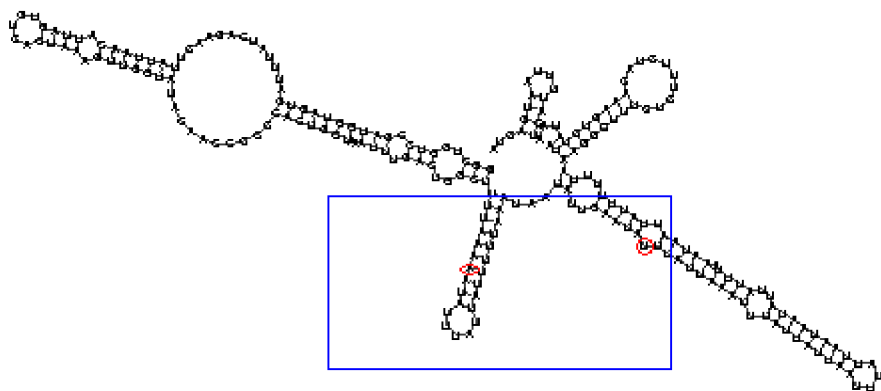

Cand5

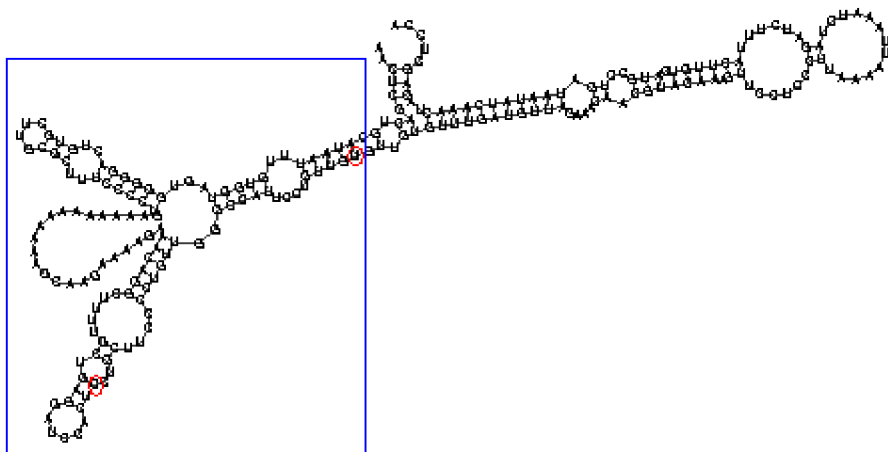

Cand6

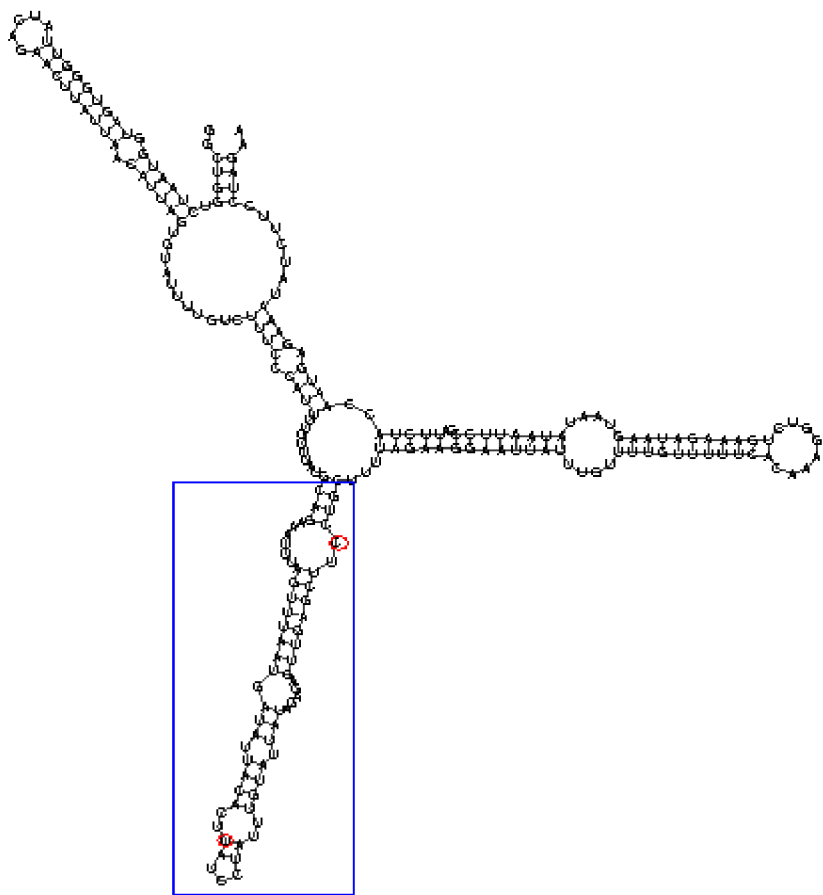

Cand7

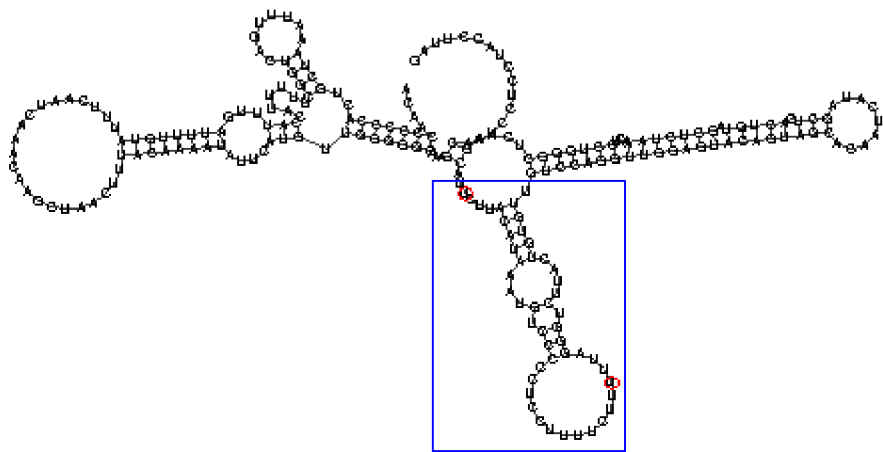

Cand8

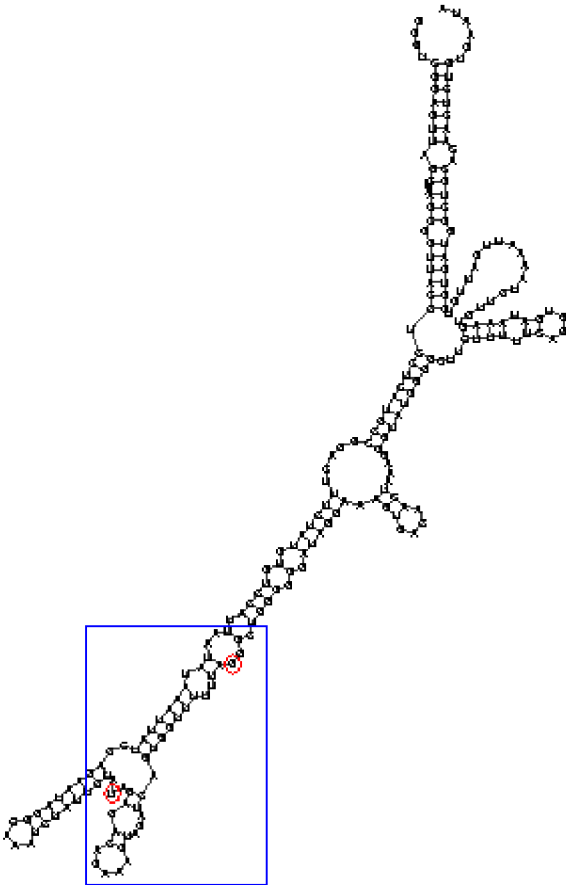

Cand9

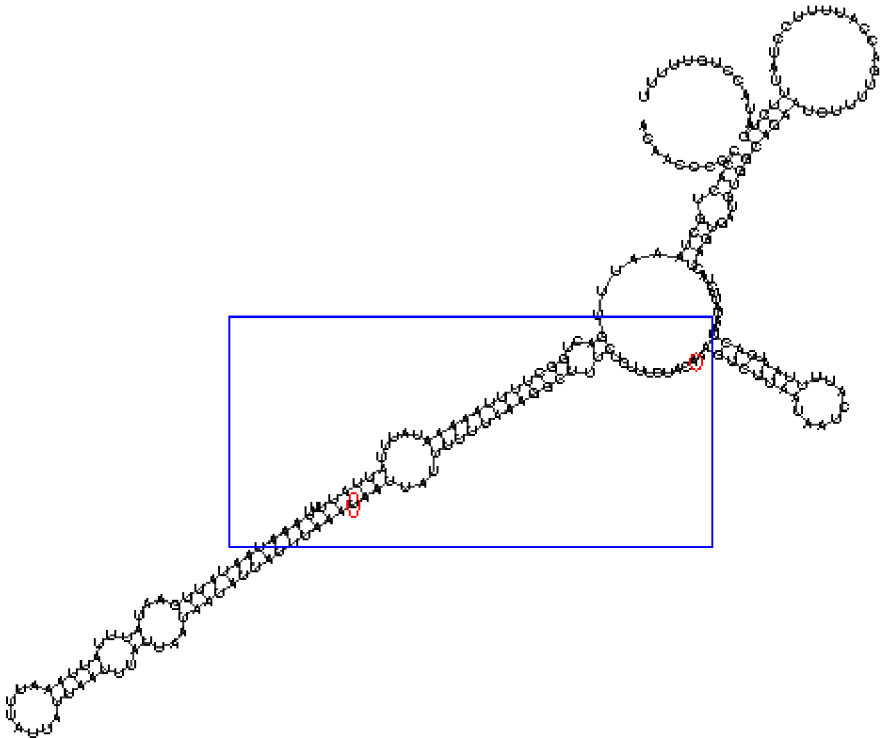

Cand10

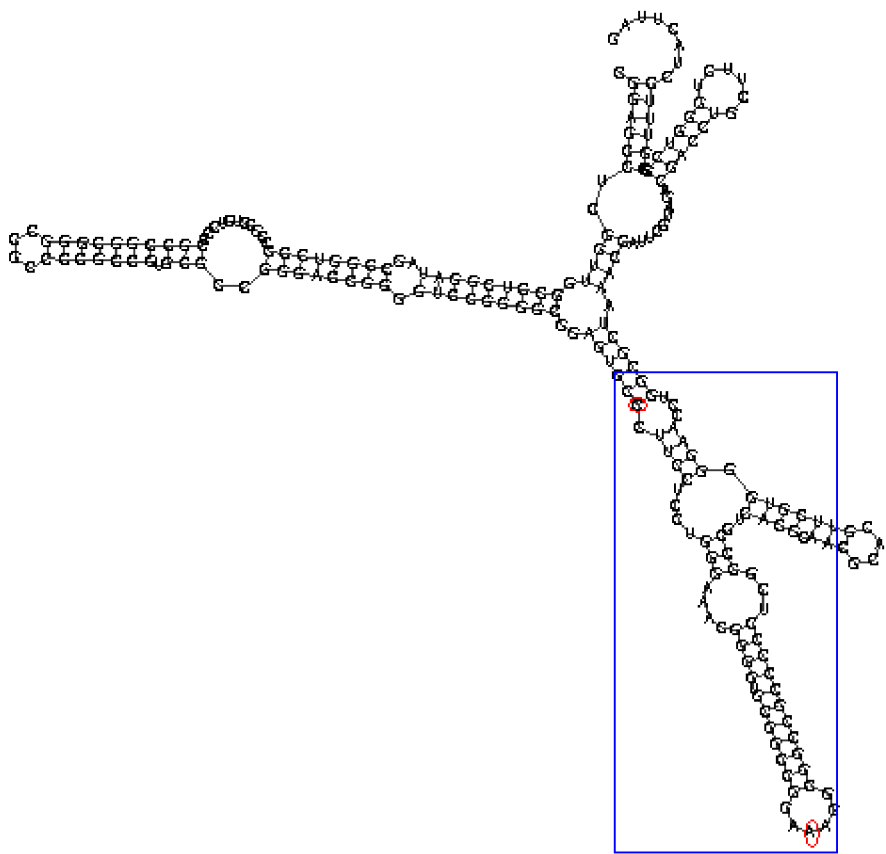

Cand11

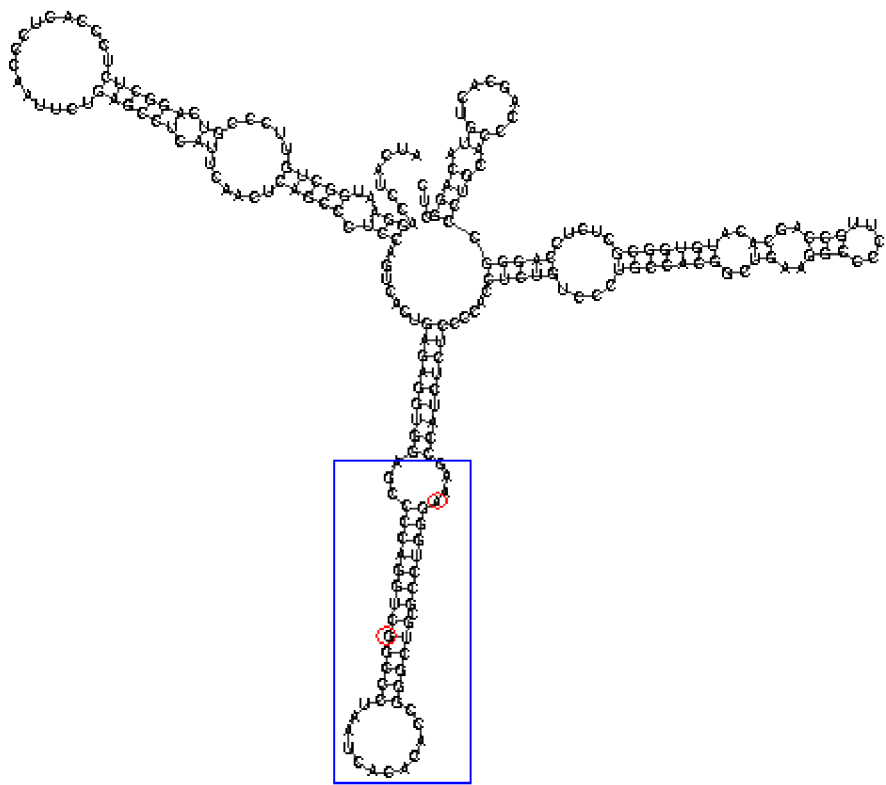

Cand12

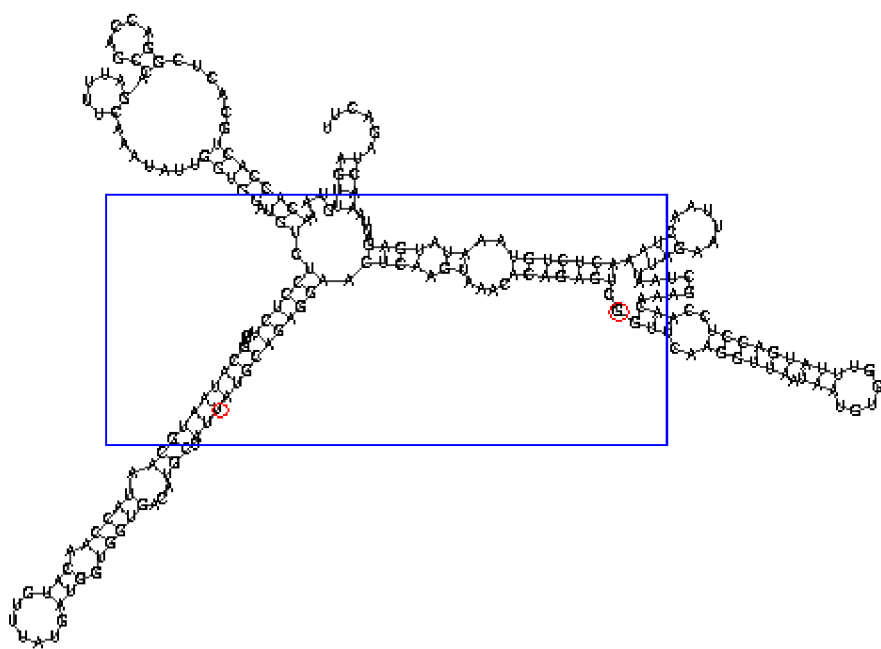

Cand13

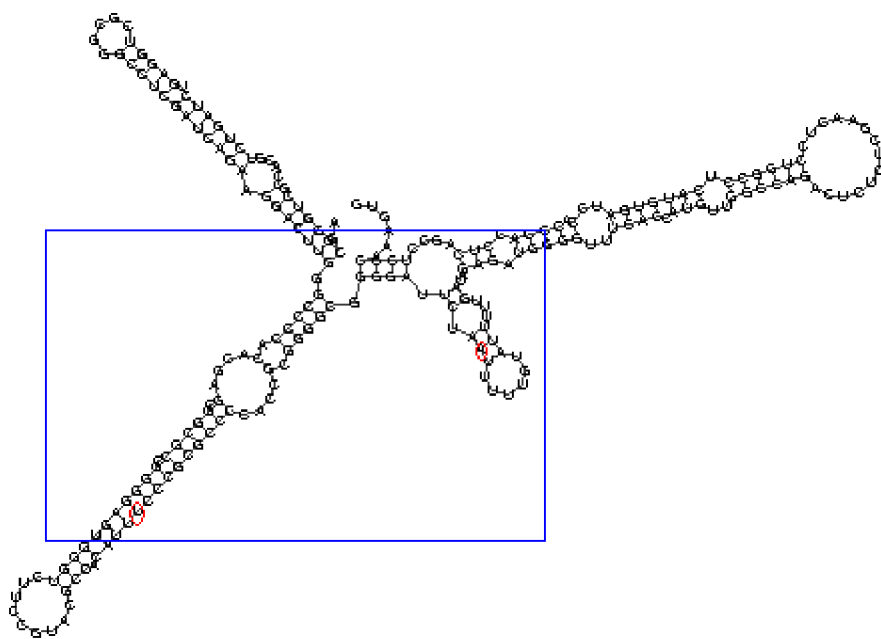

Supplement: Additional file 8 — Secondary structures of miRNA putative precursors as predicted by RNAfold. The structures were predicted using default parameters of the computational tool. The RNAFold webserver can be found at http://rna.tbi.univie.ac.at/cgi-bin/RNAfold.cgi. [file 1471-2164-14-735-S8.pdf]
