# Supplementary material for: High-throughput sequencing of small RNA transcriptomes reveals critical biological features targeted by microRNAs in cell models used for squamous cell cancer research
Source: BMC Genomics. 2013 Oct 26;14:735. doi: 10.1186/1471-2164-14-735 (PMC3870990; doi:10.1186/1471-2164-14-735)
Supplement: Additional file 9 — Identification of functional parts (mature miRNAs) within putative precursor sequences using the computational tool MatureBayes. On the precursor sequences defined in this paper as possible miRNA candidates we highlight the sequenced read (green), mature miRNA prediction at the 3’ stem (red) and mature miRNA prediction at the 5′ stem (blue). The identification of mature sequences followed the procedures in the site http://mirna.imbb.forth.gr/MatureBayes.html [file 1471-2164-14-735-S9.pdf]

Cand3

UUGUCAGAUGAGUAGGUUGCGA

UGUAAAUUUGUUUGAGUUCAUUGUAGAUUCUGGAUAUUAGCCCUUUGUCAGAUGAGUAGGUUGCGAAAAUUUUCUCCCAUUUUG

GGAAGCUCUUUAGUUUAAUUAG

UAGGUUGCCUGUUCACUCUGAUGGUAGUUUCUCUCGCUGUGUGGAAGCUCUUUAGUUUAAUUAGAUCCCAUUUGUCAAUUUUGGC

UUUUGUUGCCAUUGCUUUUGGUGUUUUAGACAUGAAGUCCUUGCCCAUGCCUAUGUCCUGA

Cand4

AACAUUAGUGUCACUAAAGUUG

GGCUGGUCCGAUGGUAGUGAUUUAUCAGAACUUAUUAACAUUAGUGUCACUAAAGUUGGUAUACAACCCCCCACUGCUAAAUUUG

ACUGGCUUUUAAAAAUAUUUAUUAUUUUUAAAUAAUAUUGAAUAUUUAUUAAAUUUAUUAUUAAUUUAUUAAUAAUAUUAUUUA

AAAGUCUUAAUAAUCAUUUUAA

AAUAAUUAUUUUUUAAAGGCUUUCUCUUUCUACAAAGUCUUAAUAAUCAUUUUAAUGACUA

Cand9

UGCUAAAUUUGACUGGCUUUUA

ACAACCCCCCACUGCUAAAUUUGACUGGCUUUUAAAAAUAUUUAUUAUUUUUAAAUAAUAUUGAAUAUUUAUUAAAUUUAUUAU

AAUUUAUUAAUAAUAUUAUUUA

UAAUUUAUUAAUAAUAUUAUUUAAAUAAUUAUUUUUUAAAGGCUUUCUCUUUCUACAAAGUCUUAAUAAUCAUUUUAAUGACUA

AUAUUCUACUAGUGAUGUGGCAGAAUCUUUUGACCAUUUUCCUAUUUCUGAUACCUGUUUUU

Cand11

AUGGCUGUUCCCGUCAGGCUCU

AUCAUCCAGGAAUGGCUGUUCCCGUCAGGCUCUCCCACUCCCAAUUCUGAGCCUCAUUCAACUCAGCCCUCCAGUCACUGAGAGGU

AAGG

GGAGCCCCAGGUCGGCCCUAAUCACACACCGGGCUGCGCCUGGGAAAGCCAUCUCUCCCCACCUCUGUCCCUGCCACGGCUGAAGG

CCCCUUGCCAGCACAUGU

CCCCUUGCCAGCACAUGUGGCGCUCUCCAGGGCCCUGCACCCAGCACUGUACAGGCUC

Cand12

GGACCAGCCAGAUUUUCAAAUA

AGUUGUAAACACCACUGCACUCGGACCAGCCAGAUUUUCAAAUAUUGGUGGAUGUCUCCUCUAGAAGCAUAAUGCAAUACCAACA

UGGUGACAUGCUAUUAUGCAGA

UGUUUAUGAUGGUGGUGACAUGCUAUUAUGCAGAGGAACUCAAGUAAACACAGAGUCGGUUCAAGGUUAAUAAUGUGGUUUAUG

ACCUCCAACAAAGCUAAUUAGAAUUAACUAAACUCUGUAAAUAUGAGAUUAAACUAGACUU
