# Supplementary material for: High-throughput sequencing of small RNA transcriptomes reveals critical biological features targeted by microRNAs in cell models used for squamous cell cancer research
Source: BMC Genomics. 2013 Oct 26;14:735. doi: 10.1186/1471-2164-14-735 (PMC3870990; doi:10.1186/1471-2164-14-735)
Supplement: Additional file 10 — Sequencing results from clinical samples. [file 1471-2164-14-735-S10.pdf]

	196M	196T	240M	240T	277M	277T	296M	296T
**Filtered reads**	2.301.939	4.396.489	7.436.013	2.523.693	17.263.580	36.845.055	11.354.744	4.760.347
**Reads matching mirBase v18**	140.004	129.499	1.085.799	327.542	1.639.635	141.612	885.503	178.876
**Reads matching mature miRNAs**	111.889	91.870	716.375	249.910	1.196.691	112.315	688.186	141.920
**Reads mapped to genome and not matching v18**	87.045	89.426	1.425.989	283.404	1.225.542	342.974	914.833	185.889
**Total of reads mapped to genome**	2.528.988	4.615.414	9.947.801	3.134.639	20.128.757	37.329.641	13.155.080	5.125.112

	306M	306T	321M	321T	333M	333T	349M	349T
**Filtered reads**	3.742.828	6.031.268	5.899.872	5.307.854	4.630.998	3.117.103	2.820.070	1.201.678
**Reads matching mirBase v18**	724.222	473.759	495.901	384.204	83.782	566.968	685.464	183.167
**Reads matching mature miRNAs**	181.942	368.185	368.778	305.118	62.899	441.013	464.219	136.228
**Reads mapped to genome and not matching v18**	948.421	641.973	456.454	295.902	136.927	694.410	506.981	292.061
**Total of reads mapped to genome**	5.415.471	7.147.000	6.852.227	5.987.960	4.851.707	4.378.481	4.012.515	1.676.906
