# Supplementary material for: High-throughput sequencing of small RNA transcriptomes reveals critical biological features targeted by microRNAs in cell models used for squamous cell cancer research
Source: BMC Genomics. 2013 Oct 26;14:735. doi: 10.1186/1471-2164-14-735 (PMC3870990; doi:10.1186/1471-2164-14-735)
Supplement: Additional file 11 — Fold-change between cells (SCC25 vs keratinocytes) and Clinical Samples (tumor vs tumor-free samples). Fold-change in blue indicates overexpression in keratinocytes or tumor-free sample. Fold-change in red indicates overexpression in the cell line or in tumor sample. [file 1471-2164-14-735-S11.pdf]

Additional File 11: Fold-change between cells (SCC25 vs keratinocytes) and Clinical Samples (tumor vs tumor-free samples)

MiRNAs	Cells	196
hsa-mir-1306	5.82	2.87
hsa-mir-130a	3.00	2.49
hsa-mir-193b	2.08	21.03
hsa-mir-296	14.24	8.03
hsa-mir-29b	2.22	2.18
hsa-mir-409	22.00	2.19
hsa-mir-4315	3.45	25.55
hsa-mir-548ar	3.24	2.74
hsa-mir-708	12.08	4.43
hsa-mir-92b	2.16	9.61
hsa-mir-628	-4.64	-5.48

MiRNAs	Cells	240
hsa-mir-144	3.88	5.19
hsa-mir-193a	2.79	2.70
hsa-mir-199a	5.89	2.16
hsa-mir-495	17.47	3.65
hsa-mir-132	-2.43	-4.23
hsa-mir-181a	-5.69	-2.56
hsa-mir-181b	-3.24	-3.65
hsa-mir-196a	-2.65	-19.04
hsa-mir-619	-2.51	-15.87
hsa-mir-628	-4.64	-10.16
hsa-mir-99b	-2.01	-2.31

MiRNAs	Cells	296
hsa-let-7b	2.03	4.35
hsa-let-7i	3.67	4.38
hsa-mir-125b	4.88	4.30
hsa-mir-133a	2.05	9.54
hsa-mir-144	3.88	37.66
hsa-mir-199a	5.89	3.25
hsa-mir-29b	2.22	3.42
hsa-mir-376a	14.56	7.89
hsa-mir-376c	15.64	5.22
hsa-mir-378i	2.36	7.83
hsa-mir-382	13.59	5.63
hsa-mir-409	22.00	2.47
hsa-mir-4302	2.72	12.86
hsa-mir-495	17.47	4.78
hsa-mir-103a	-2.50	-2.42
hsa-mir-196a	-2.65	-4.99

MiRNAs	Cells	277
hsa-let-7b	2.03	21.79
hsa-let-7i	3.67	33.88
hsa-mir-125b	4.88	48.95
hsa-mir-1306	5.82	7.28
hsa-mir-130a	3.00	14.48
hsa-mir-133a	2.05	91.47
hsa-mir-144	3.88	33.59
hsa-mir-193a	2.79	67.23
hsa-mir-193b	2.08	22.77
hsa-mir-199a	5.89	42.86
hsa-mir-22	2.15	34.63
hsa-mir-27a	2.44	24.85
hsa-mir-296	14.24	6.20
hsa-mir-29b	2.22	68.71
hsa-mir-29c	3.06	78.48
hsa-mir-34a	2.10	10.20
hsa-mir-376a	14.56	71.88
hsa-mir-376c	15.64	94.88
hsa-mir-378i	2.36	52.62
hsa-mir-382	13.59	86.90
hsa-mir-409	22.00	12.82
hsa-mir-4302	2.72	94.58
hsa-mir-495	17.47	619.42
hsa-mir-708	12.08	12.74
hsa-mir-769	2.26	3.18
hsa-mir-92b	2.16	15.90
hsa-mir-196a	-2.65	-2.07

MiRNAs	Cells	306
hsa-mir-125b	4.88	20.81
hsa-mir-133a	2.05	465.99
hsa-mir-199a	5.89	5.70
hsa-mir-376a	14.56	24.72
hsa-mir-376c	15.64	2.27
hsa-mir-409	22.00	2.44
hsa-mir-4302	2.72	9.13
hsa-mir-4315	3.45	3.63
hsa-mir-495	17.47	23.54
hsa-mir-548ar	3.24	3.96
hsa-mir-103a	-2.50	-233.50
hsa-mir-132	-2.43	-9.01
hsa-mir-15a	-2.58	-3.98

hsa-mir-205	-2.23	-2.57		hsa-mir-17	-2.34	-9.34
hsa-mir-301a	-3.84	-4.18		hsa-mir-181a	-5.69	-3.27
hsa-mir-940	-3.35	-17.20		hsa-mir-18a	-2.15	-59.25
hsa-mir-99b	-2.01	-3.61		hsa-mir-196a	-2.65	-12.98
				hsa-mir-205	-2.23	-61.01
MiRNAs	Cells	321		hsa-mir-20a	-3.38	-49.94
hsa-mir-125b	4.88	4.17		hsa-mir-301a	-3.84	-13.40
hsa-mir-144	3.88	6.75		hsa-mir-940	-3.35	-8.16
hsa-mir-193a	2.79	2.05		hsa-mir-99b	-2.01	-7.61
hsa-mir-199a	5.89	2.21				
hsa-mir-34a	2.10	2.23		MiRNAs	Cells	333
hsa-mir-4302	2.72	2.90		hsa-mir-100	-2.84	-39.00
hsa-mir-708	12.08	3.73		hsa-mir-103a	-2.50	-9.48
hsa-mir-18a	-2.15	-3.33		hsa-mir-10a	-7.73	-13.81
hsa-mir-196a	-2.65	-46.73		hsa-mir-132	-2.43	-9.25
hsa-mir-301a	-3.84	-2.96		hsa-mir-15a	-2.58	-17.13
hsa-mir-940	-3.35	-2.50		hsa-mir-17	-2.34	-5.39
				hsa-mir-181a	-5.69	-8.08
MiRNAs	Cells	349		hsa-mir-181b	-3.24	-17.37
hsa-mir-125b	4.88	6.14		hsa-mir-181c	-10.24	-6.28
hsa-mir-133a	2.05	29.35		hsa-mir-18a	-2.15	-3.66
hsa-mir-144	3.88	3.08		hsa-mir-196a	-2.65	-5.46
hsa-mir-29c	3.06	2.71		hsa-mir-20a	-3.38	-2.30
hsa-mir-376a	14.56	22.99		hsa-mir-301a	-3.84	-5.81
hsa-mir-376c	15.64	47.95		hsa-mir-30c	-3.18	-9.07
hsa-mir-378i	2.36	3.60		hsa-mir-4301	-2.32	-7.48
hsa-mir-4302	2.72	5.55		hsa-mir-619	-2.51	-4.99
hsa-mir-495	17.47	5.43		hsa-mir-625	-2.21	-6.87
hsa-mir-92b	2.16	2.17		hsa-mir-628	-4.64	-2.95
hsa-mir-103a	-2.50	-4.20		hsa-mir-940	-3.35	-9.97
hsa-mir-17	-2.34	-2.35		hsa-mir-99b	-2.01	-15.29
hsa-mir-18a	-2.15	-5.23				
hsa-mir-196a	-2.65	-72.98				
hsa-mir-301a	-3.84	-5.25				
hsa-mir-940	-3.35	-3.63				
hsa-mir-99b	-2.01	-2.83				
